# The interdependency structure in the Mexican stock exchange: A network approach

**DOI:** 10.1371/journal.pone.0238731

**Published:** 2020-10-29

**Authors:** Erick Treviño Aguilar

**Affiliations:** Unidad Cuernavaca del Instituto de Matemáticas, Universidad Nacional Autónoma de México, Cuernavaca, Mexico; Beijing University of Posts and Telecommunications, CHINA

## Abstract

Our goal in this paper is to study and characterize the interdependency structure of the Mexican Stock Exchange (mainly stocks from Bolsa Mexicana de Valores) for the period 2000-2019 which provide a one shot big-picture panorama. To this end, we estimate correlation/concentration matrices from different models and then compute centralities and modularity from network theory.

## Introduction

In this paper we investigate the interdependency structure of daily returns in the Mexican stock exchange market. To this end, we build a database of *free and publicly* available time series of main stocks for the period 2000-2019 and conduct our study in stages that are then put together to give a unified treatment to our main topic of interest here which is the interdependency structure of daily log-returns in the Mexican stock exchange.

In the first stage we focus on the estimation of partial correlations of log returns of daily prices. The reasons for focusing on partial correlations are the following. Given a collection of Gaussian series *A*_1_, …, *A*_*n*_, a zero partial correlation between *A*_1_ and *A*_2_ implies that *A*_1_ and *A*_2_ are conditionally independent, meaning that *A*_1_ and *A*_2_ could still be (unconditionally) correlated but only through a third factor adapted to the other series *A*_3_, …, *A*_*n*_. There are of course different methods of estimating a covariance/correlation/concentration matrix and we have selected a estimation based on a specific class of Markovian Random Fields (MRF) which in the statistical literature is well known under the name Gaussian Graphical models (GGm). The adjective “graphical” emphasizes the fact that attached to the probabilistic model there is a graph in which edges express conditional dependencies, from which a very convenient visual representation is obtained. There are three reasons for working with this model. First of all, the benefit of the already mentioned visual representation provided by the model. The second is that we have decided to study the period 2000-2019 on a yearly basis. There is a trade-off to this treatment. On the one hand, short periods of time reduce problems with heavy tails. On the other, the number of stocks in each year is a significant proportion of the available observations. Hence, a *lasso*-regularized estimation is useful in this context which is inbuilt in the estimation of a GGm. Third, we want an estimation that filters out a “noisy” correlation selecting only clear relationships between two series, again this is provided by the *lasso*-regularized estimation. Loosely speaking, we follow a partial correlations selection approach which conceptually is comparable to a covariance selection approach [1]. Once partial correlations matrices have been estimated we provide a list of *stylized facts* from them. Then, taking the graphs constructed from the matrix of partial correlations as its adjacency matrix, we compute eigen-, between-, and degree- centralities.

In a second stage we compute networks based on matrices of Tail-dependence coefficients ([2]) of every pair of log returns. This coefficient quantifies the relationship of lower tails and captures dependencies in the events of negative returns.

In the third stage we estimate correlation matrices of time series (estimated through a Multivariate Dynamic Conditional Correlation GARCH specification). Then, we apply a technique from network-theory based on those correlation matrices known as the maximization of a modularity objective function. This procedure will provide a community structure.

As part of our main goal of studying the interdependency structure of the Mexican stock exchange we contribute to the existing literature on financial networks concerning the following aspects. First of all, many papers focus on financial networks constructed from Pearson correlation matrices but much less papers focus on financial networks constructed from partial correlations and/or Tail-dependence matrices, as we do here. Moreover, from the few papers focusing on partial correlation matrices, we are not aware of any of them applying the Gaussian Graphical model we consider in this paper. As a consequence no paper has previously compared network-structures from the three afore-mentioned different matrices (Pearson correlation, partial correlations and Tail-dependence) as we do in the present paper. The premise is that different underlying matrices yield different network-topologies.

Many papers study aggregated financial indices and do not go into the details of analyzing at the level of stocks in the selected market, hence missing the point of analyzing interdependency at the level of individual stocks, where the network perspective could represent an advantage to support financial decisions; see e.g., [3, pp. 8, inmediately before the section “Factor models”]. For example, we find that the main index in the Mexican stock exchange denoted IPC (not to be confused with the Index of Consumer Price level.) is “influential” with respect to degree- and eigen- centrality but the intensity varies with respect to which matrix the network is based on. However, it is not influential with respect to betweenness-centrality. Thus, the index does not convey all the information in the market; compare e.g., again with [3, pp. 10, first paragraph].

Few papers focus on the case of Mexico, a representative market in the region which some studies have found to be a connecting node between Latin American and US markets, hence playing a key role; see [4]. For example, [5] is an early paper studying stock market integration between Latin American countries and the US. This includes Mexico, but only as part of the region with no particular focus.

## Materials and methods

### Background

The classical Markowitz theory of portfolio selection illustrates the relevance of asset correlation matrices for financial decisions. However, the nontriviality of correlation estimation from empirical data has been known for a long time, see e.g., [6]. Moreover, in contexts where sparse correlation (specially for partial correlation) matrices are expected, it is desirable to have a systematic method to discard “non-clear correlations” and account for a parsimonious model as motivated by [1]. As we mentioned in the introduction, in this paper we choose to apply a GGm for a parsimonious estimation of concentration/partial-correlation matrices. Estimation of Tail-dependence coefficients are based on the non-parametric estimator in [7]. Pearson-correlation matrices are estimated from a multivariate GARCH model.

Beyond the estimation problem, it is useful to have tools that, starting from matrices, are able to generate metrics providing snapshots of the market from which quick but trustable diagnosis are available. Situations in which this is desirable include, from the point of view of an investor, the decision to rebalance a portfolio, and from the point of view of a regulator, interventions in the market in order to lessen the contagion of a shock in a specific sector.

We find those tools in the theory of random graphs, specially in the form of metrics (in this paper, degree-, eigen- and betweenness- centralities) which classify the interconnectedness of stocks and a global metric (the modularity computed from correlation matrices) to detect *communities* of stocks.

The approach described integrates into the outline of [8] and is a very active research area; see e.g., the survey in [9]. However for the Mexican stock exchange there has been little research in this direction. In the next section we present related literature. Note however that we do not pretend to provide an exhaustive revision of this active topic which deserves a survey by its own, but to give a brief panorama of current research in this area.

### Stock markets from GGm, random graphs, and network-theory approaches

Gaussian graphical models, Random graphs, and Network theory approaches in a financial context is an active research area attracting more and more attention with an increasing number of papers; see e.g., the survey in [9]. The following is a non exhaustive list merely describing different approaches and applications.

Papers in finance reporting an approach related to a graphical model include [10, 11] and [12]. However, none of these papers focus on asset prices. Theoretical background on graphical models can be found in [13, 14] and [15].

Papers studying financial networks based on partial correlations include [4, 16, 17] and [3]. Papers with applications based on a network approach in a financial context include (a) spillover effects and shocks contagion, [17–20] and [21] (b) portfolio selection, [22, 23] and [24] (c) detection of stock prices manipulation [25] and (d) portfolio diversification [26]. Papers studying financial networks based on the distributions’ tails of prices/log-returns include [22, 27–30] and [31].

Some studies determine power laws for degree connectedness defined by assets correlation matrices; see [16, 26, 32–34] and [35]. Papers studying community detection in a financial context include [16, 36–38] and [39]. See [40] for a survey on methods for community detection.

Minimal Spanning Trees applied to financial market ranking include [4, 35, 41, 42] and [43]. Random matrix theory for correlation matrices has been presented in [17, 36, 44] and [45].

### Subprime crisis

**Fig 1:**
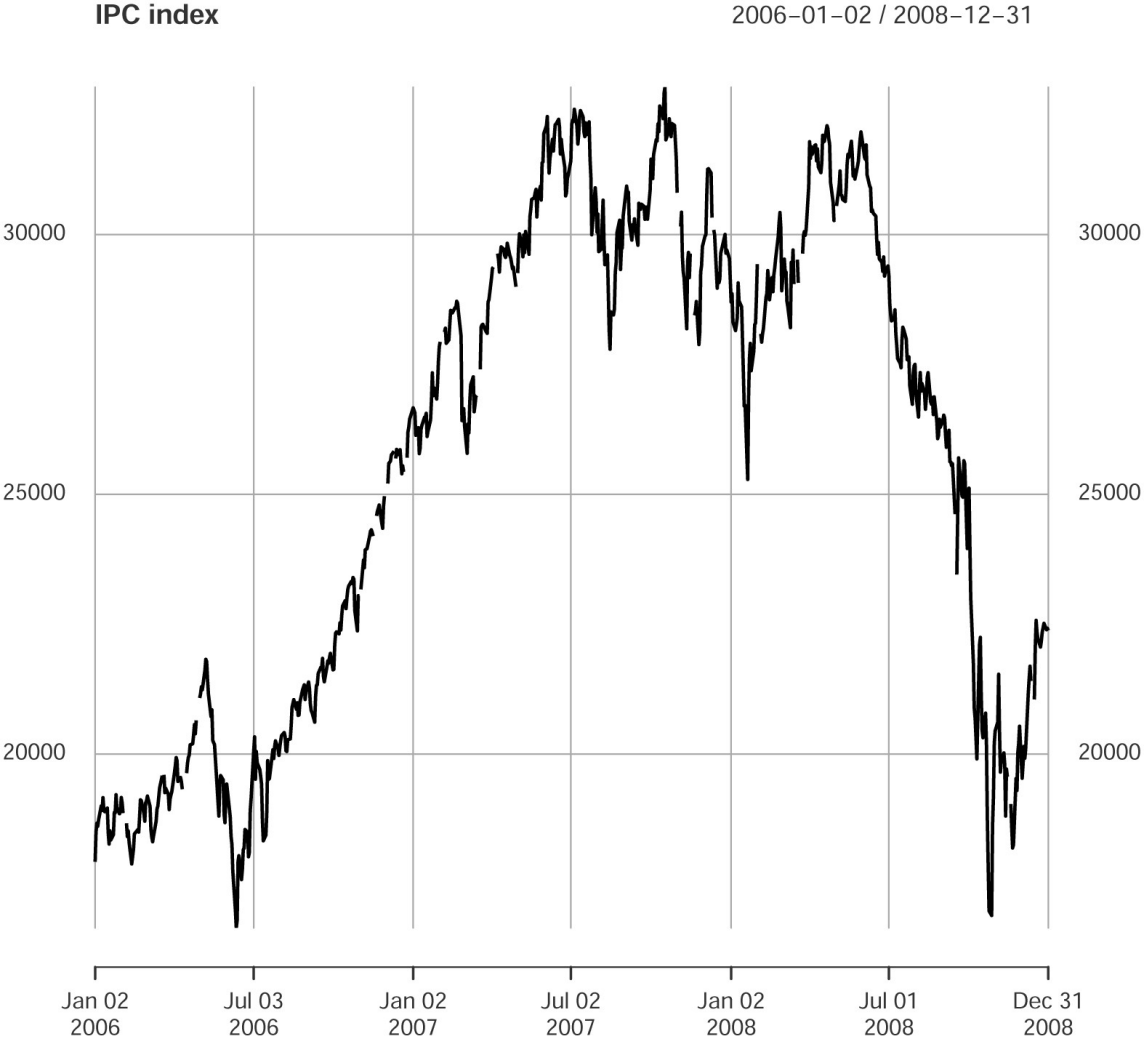
IPC index. Time series for the period 2006-2008.

According to [46] there indeed existed an impact from the 2007-2008 subprime crisis on the Mexican economy. Mainly due to two shocks, first, a decline in Mexico’s exports and second, a constrained access to international financial markets, thus evidencing an integration of the Mexican stock exchange with the US market. A phenomenon documented by some authors; see e.g., [47–49]. Fig 1 illustrates price levels for the main IPC index in the Mexican stock market for the years 2006, 2007 and 2008. It can be argued the presence of a bullish market on 2006 while on the second semester of 2008 the market turned bearish. Not surprising and reported by some authors [50] and [51]. Later we will go beyond a visual examination and confirm by a multivariate GARCH model through a shift from positive to negative intercepts on log returns of each time series of the period. However, quite interesting, we will show that the partial-correlations interdependency structure of the Mexican financial market does not exhibit a drastic change as consequence to that shock, see Fig 3.

### Data

We constructed a database of daily closing prices from *free and publicly* available information at Yahoo.Finance website which we downloaded through the R package quantmod. The complete list of analyzed stocks can be found in the S1 Table. The frequency of data is daily in a span of time comprising 01-01-2000 to 31-12-2019. We have considered throughout the paper time series of log returns: $R_{t}\left(i\right)=\log(\frac{S_{t+1}\left(i\right)}{S_{t}\left(i\right)})$ where *S*_*t*_(*i*) is the price level at time *t* of stock labeled *i*.

**Fig 2:**
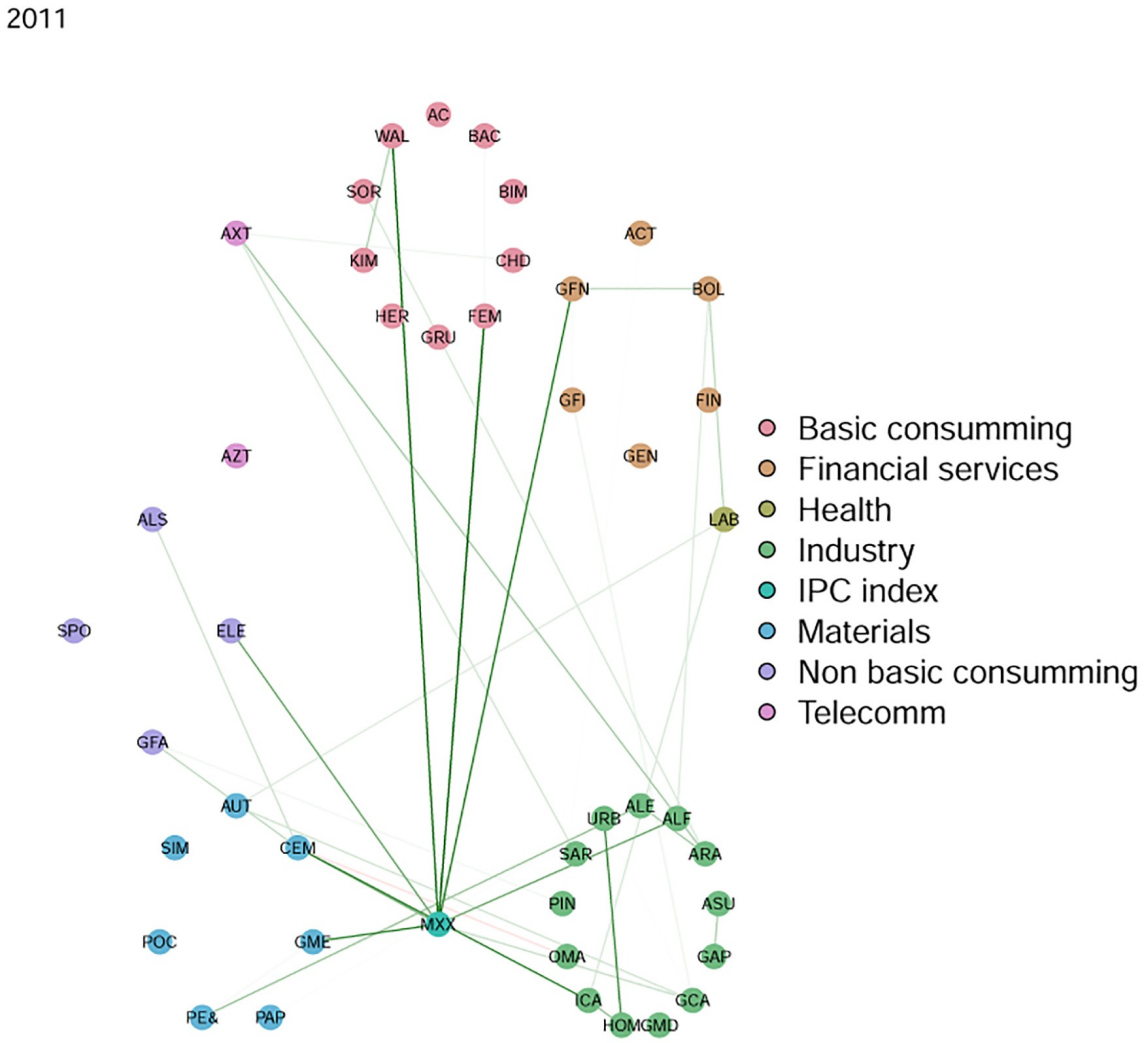
A network in which stocks can be identified in their sectors.

**Fig 3:**
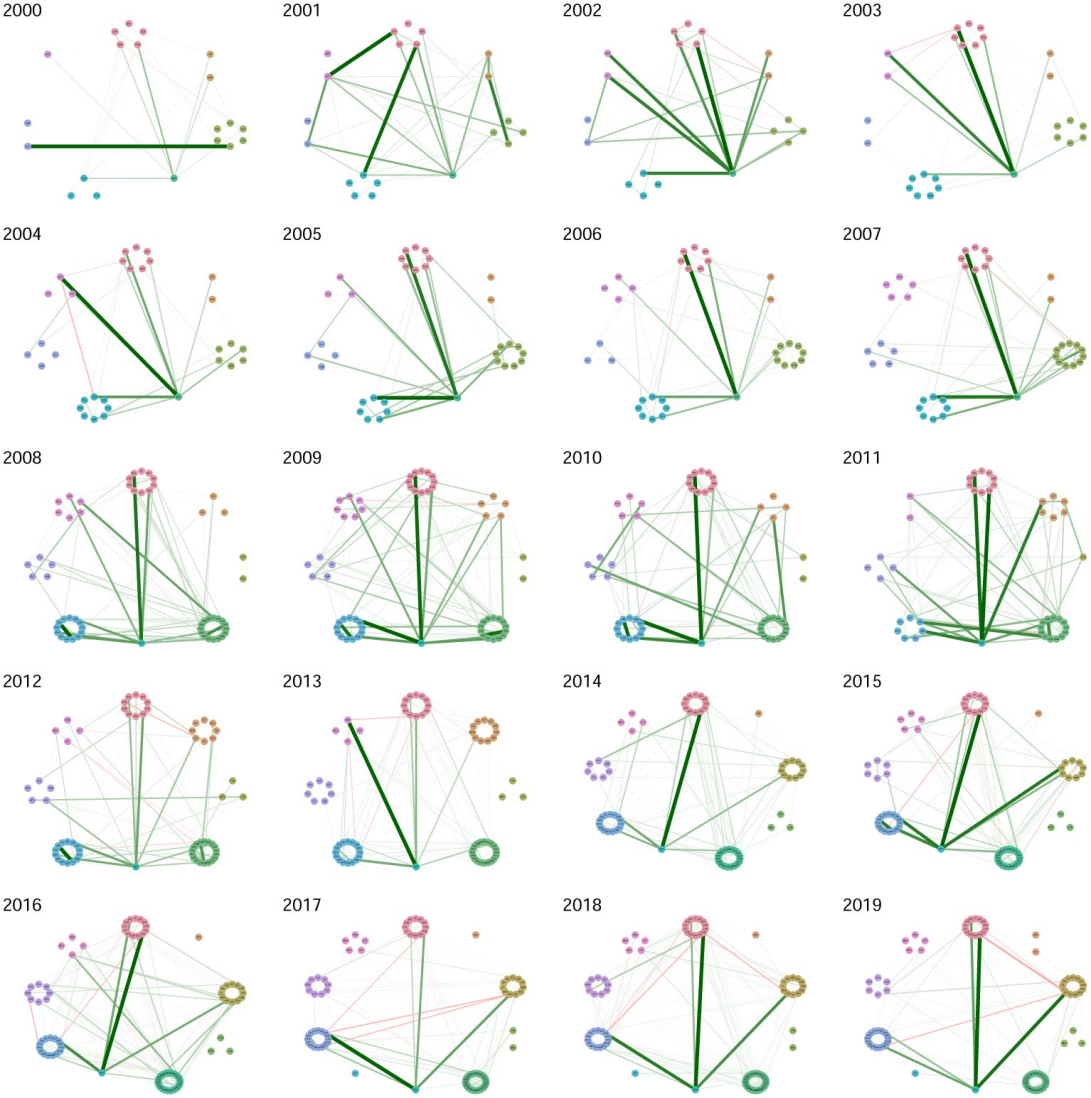
Graphs associated with partial correlation matrices, by year, in the period 2000-2019. For a given edge the green color (respectively red color) represents a positive (respectively negative) relationship. Edge’s width represents the strength of correlation. Vertexes are grouped according to industrial sector.

Data is organized in windows of one year (from january to december) and we applied a filtering process in two steps. In the first step, for each year, stocks in the market with the most complete information were selected. The criterion was that only stocks with more than 90% of all the available dates were selected. Then, in a second step, stock prices not having a minimum of variance in moving windows spanning 30 dates were discarded. This filtering process already presents the interesting fact of a positive evolution of the Mexican stock exchange in the sense of an increase in activity. Indeed, as we go forward through the years, more and more time series of stock prices satisfy the filtering process, evidencing an evolution in terms of more activity in the market with more variability of prices and more quotes. Visual evidence can be found in Fig 3. An important aspect of this work will be to consider how industrial sectors are interconnected. Here we consider a list of sectors obtained from the Bolsa Mexicana de Valores (BMV) classification. These are listed in Table 1. Fig 2 presents an estimated network in which stocks can be identified in its sector.

**Table 1:** Industrial sectors.

	Sector	No. Stocks	Description
1	Basic consumming	21	Manufacturers and distributors, food and beverage companies
2	Energy	2	Energy producers, equipment, services and distribution
3	Financial services	25	Includes banks, financial and insurance firms
4	Health	4	Care providers, equipment, supplies and pharmaceuticals
5	Industry	36	Include companies providing equipment and services in the productive chain
6	IPC index	1	Main index in the Mexican stock exchange
7	IT	1	Software and Hardware for information technologies
8	Materials	22	Include companies providing input materials in the productive chain
9	Non basic consumming	19	Includes retailers, consumer service providers and consumer durables
10	Telecomm	9	Includes wireless providers, internet service providers and satellite companies

## Results and discussion

### Gaussian graph model

#### Markovian random fields

In this section we start with the basic definition of a MRF which is the fundamental probabilistic concept from which a GGm is defined. Let us introduce a graph *G* = (*V*, *E*) with a set of nodes *V* = {1, …, *n*} and edges *E*. Recall that a complete subgraph of *G* is called a *clique*. We denote by C the class of *maximal cliques* of the graph *G*. Let us start with a given a random vector $\vec{X}=\left(X_{1},\ldots ,X_{n}\right)$ with multivariate cumulative distribution function *p*. Then, the vector $\vec{X}$ has a *Gibbs distribution* compatible with the graph *G* if its distribution has a representation

$$\begin{array}{c} p\left(x_{1},\ldots ,x_{n}\right)=\frac{1}{Z}\prod_{C\in C}\psi_{C}\left(x_{C}\right), \end{array}$$

where ${\left\{\psi_{c}\right\}}_{C\in C}$ are suitable functions and *x*_*C*_ denotes a vector in which only the indexes of *C* appear. Gibbs distributions are characterized through different Markov properties. To this end, we need a notation. For *A* ⊂ *V*, $A=(A_{i_{1}},\ldots ,A_{i_{k}})$, the notation ${\vec{X}}_{A}$ denotes the vector $\left(X_{i_{1}},\ldots ,X_{i_{k}}\right)$. The following list provides the Markov properties:

1. $\vec{X}$ is a MRF with respect to *G* if it has the Markov property: For any pair *i*, *j* ∈ *V* with *i* ≠ *j* and non adjacent in the graph *G*, the random variables *X*_*i*_ and *X*_*j*_ are conditionally independent on all the other variables. We denote this conditional independency by:
  $$\begin{array}{c} X_{u}⊥X_{v}|{\vec{X}}_{V/\{u,v\}}. \end{array}$$
2. $\vec{X}$ is locally a MRF with respect to *G* if: For each *v* ∈ *V*, the random variable *X*_*v*_ is conditionally independent of all other variables which are not neighboors (they are not adjacent). We denote this by
  $$\begin{array}{c} X_{v}⊥{\vec{X}}_{V/neighborhood(v)}|{\vec{X}}_{neighborhood(v)}. \end{array}$$
3. $\vec{X}$ is globally a MRF if: For two disjoint subsets *A*, *B* ⊂ *V*, the vectors ${\vec{X}}_{A}$, ${\vec{X}}_{B}$ are conditionally independent on a separating set *S* ⊂ *V*. We denote this by:
  $$\begin{array}{c} {\vec{X}}_{A}⊥{\vec{X}}_{B}|{\vec{X}}_{S}. \end{array}$$

We continue with a fundamental equivalence result; see [52, Chapter 7].

**Theorem 1 (Hammersley-Clifford)**. *Assume that the cumulative distribution p of* $\vec{X}$ *is defined in a finite state space and is positive valued. Then p is a Gibbs distribution if and only* $\vec{X}$ *satisfies any of the above listed Markov properties*.

For a list of Gibbs distributions see [13, Section 3]. In this paper we will work with the following specific Gibbs distribution (hence, specific MRF and specific GGm)

$$\begin{array}{c} p_{\theta }\left(x\right)=\exp\{\theta \cdot x+\frac{1}{2}\sum_{i=1}^{n}\sum_{i=1}^{n}\Theta_{i,j}x_{i}x_{j}-A\left(\theta \right)\}. \end{array}$$ (1)

where *n* is the dimension of the random vector $\vec{X}$, and *A*(⋅) is a normalizing constant; see [13, Example 3.3] for more details. The MRF model in (1) also specifies the GGm we will work with. Indeed, (1) does not apriori specify any graph, but from the set of parameters $\Theta_{i,j}\in R$ we derive a partial correlation matrix which indeed can be seen as the adjacency matrix of a weighted graph.

#### Covariance selection

Let $\Sigma =(\Sigma_{i,j})$ be the covariance matrix of a random vector $\vec{R}=\left(R_{1},\ldots ,R_{n}\right)$ with multivariate Gaussian distribution. A zero component *Σ*_*i*,*j*_ = 0 expresses marginal independence between *R*_*i*_ and *R*_*j*_. The inverse matrix $J≔\Sigma^{-1}$ is the so-called concentration matrix. It has the property that a zero component *J*_*i*,*j*_ = 0 expresses conditional independence; see e.g., [53, Thm. 9.2.1] or for complex distributions [15, Thm 7.1 p. 117].

It is possible from further considerations that many components *J*_*i*,*j*_ are expected to be zero. In this case, it is desirable to have a statistical procedure to estimate the distribution taking into account such information. One such procedure is the so-called covariance selection in [1]. Grounded on maximum likelihood, it provides a framework to test for zero partial correlations. Recent research on covariance selection focuses on sparse large dimensions in which the number of variables is large but there are also many variables which are conditionally independent; see [54]. Thus, for such structures the concentration matrix is sparse and the lasso (least absolute shrinkage and selection operator; also lasso or LASSO) method introduced by [55] is fundamental for statistical estimation and variable selection. Indeed, the Gaussian Graphical model that we are going to use is nodewise estimated through a lasso procedure. For the lasso implementation we use the R package mgm that builds on the package glmnet. The estimations of this last package are based on the algorithm of [56]. Then, the collection of nodewise regressions are combined through an AND rule to give a unique estimation of a multivariate vector. This approach is naturally based on the asymptotic consistency results due to [54]. In particular, the estimation yields a concentration matrix **J**. Systematic presentations for graphical models can be found in [13, 14] and [15].

#### The GGm

Now we explain the specification of the GGm we are going to estimate. Let **Σ** be the covariance matrix of the log returns time series *R*(1), …, *R*(*n*). Denote by **J** the concentration matrix, **J** ≔ **Σ**^−1^. The components of the matrix **J** are given in terms of the coefficients Θ_*i*,*j*_ in Eq (1). Denote by *ρ*_*i*,*j*_ the partial correlation of *R*(*i*) and *R*(*j*). Consider the linear regressions defining partial correlations:

$$\begin{array}{c} R\left(i\right)-\mu \left(i\right)=\sum_{j\neq i}\beta_{i,j}\left(R\left(j\right)-\mu_{j}\right)+\epsilon \left(i\right) \end{array}$$ (2)

where *μ*_*i*_ is the unconditional mean of *R*(*i*) and *ϵ*(*i*) is a residual. Then

$$\begin{array}{c} \beta_{ij}=\frac{\rho_{i,j}}{\sqrt{var(\epsilon (i))var(\epsilon (j))}}. \end{array}$$ (3)

It is also true that

$$\begin{array}{c} \rho_{i,j}=\frac{-J_{i,j}}{\sqrt{J_{ii}J_{jj}}}. \end{array}$$

The adjacency matrix **P** = (*P*_*i,j*_) is defined by

$$\begin{array}{c} P_{i,i}=0\;\text{and}\;P_{i,j}=\rho_{i,j}. \end{array}$$ (4)

**Remark 1** *Let us emphasize now that the estimation of the GGm* (1) *will ultimately result in the matrix* **P** *and this matrix is our main input for the network based on the GGm*.

#### Results from GGm estimation: Stylized facts

In this section we report the results of estimating a GGm for each year in the period 2000-2019 using (1). From this estimation exercise, we get a partial correlation matrix **P** for each year in the period 2000-2019, hence twenty matrices in total. A graphical representation of partial correlations is displayed in the panel of Fig 3. In Table 2 we display partial correlations in absolute value above the threshold 0.3.

**Table 2:** Links in the rank (0.3, 1] for the period 2000-2009.

tick1	tick2	weight	year	tick1	tick2	weight	year
ELEKTRA	ICA	0.98	2000	ICHB	SIMECB	0.44	2012
FEMSAUBD	MXX	0.4	2000	AMXA	MXX	0.83	2013
AZTECACPO	SORIANAB	0.31	2001	FEMSAUBD	MXX	0.42	2013
AMXA	MXX	0.37	2002	GMEXICOB	MXX	0.48	2013
AZTECACPO	MXX	0.38	2002	ICHB	SIMECB	0.34	2013
CEMEXCPO	MXX	0.38	2002	MXX	WALMEX	0.31	2013
FEMSAUBD	MXX	0.45	2002	CEMEXCPO	MXX	0.33	2014
MXX	SORIANAB	0.32	2002	FEMSAUBD	MXX	0.56	2014
AMXA	MXX	0.59	2003	ASURB	GAPB	0.36	2015
AZTECACPO	MXX	0.31	2003	CEMEXCPO	MXX	0.36	2015
CEMEXCPO	MXX	0.36	2003	FEMSAUBD	MXX	0.42	2015
FEMSAUBD	MXX	0.35	2003	GFNORTEO	MXX	0.37	2015
MXX	WALMEX	0.73	2003	GMEXICOB	MXX	0.33	2015
AMXA	MXX	0.72	2004	ICHB	SIMECB	0.36	2015
CEMEXCPO	MXX	0.49	2004	CEMEXCPO	MXX	0.45	2016
MXX	WALMEX	0.43	2004	FEMSAUBD	MXX	0.59	2016
CEMEXCPO	MXX	0.44	2005	GFNORTEO	MXX	0.34	2016
MXX	WALMEX	0.4	2005	MXX	WALMEX	0.35	2016
CEMEXCPO	MXX	0.49	2006	ASURB	GAPB	0.33	2017
FEMSAUBD	MXX	0.41	2006	CEMEXCPO	MXX	0.75	2017
GMEXICOB	MXX	0.34	2006	FEMSAUBD	MXX	0.42	2017
MXX	WALMEX	0.77	2006	GEOB	HOMEX	0.36	2017
CEMEXCPO	MXX	0.5	2007	GFNORTEO	MXX	0.47	2017
GAPB	OMAB	0.35	2007	GMEXICOB	MXX	0.3	2017
GMEXICOB	MXX	0.31	2007	ICHB	SIMECB	0.37	2017
MXX	WALMEX	0.56	2007	ASURB	GAPB	0.32	2018
GAPB	OMAB	0.31	2008	CEMEXCPO	MXX	0.74	2018
ICHB	SIMECB	0.41	2008	FEMSAUBD	MXX	0.87	2018
MXX	WALMEX	0.37	2008	GFNORTEO	MXX	0.65	2018
CEMEXCPO	MXX	0.37	2009	GIGANTE	LIVEPOL1	0.32	2018
GAPB	OMAB	0.34	2009	MXX	WALMEX	0.41	2018
ICHB	SIMECB	0.34	2009	ASURB	GAPB	0.31	2019
MXX	WALMEX	0.35	2009	CEMEXCPO	MXX	0.39	2019
CEMEXCPO	MXX	0.35	2010	FEMSAUBD	GFNORTEO	-0.36	2019
ICHB	SIMECB	0.34	2010	FEMSAUBD	MXX	0.89	2019
MXX	WALMEX	0.36	2010	GAPB	OMAB	0.33	2019
FEMSAUBD	MXX	0.35	2011	GFNORTEO	MXX	0.81	2019
MXX	WALMEX	0.33	2011	GMEXICOB	MXX	0.55	2019
HOMEX	URBI	0.32	2012	MXX	WALMEX	0.56	2019

Partial correlations in absolute value in the interval (0.2, 0.3] are displayed in Table 3.

**Table 3:** Links in the rank (0.2, 0.3] for the period 2000-2009.

tick1	tick2	weight	year	tick1	tick2	weight	year
CEMEXCPO	MXX	0.27	2000	ASURB	GAPB	0.24	2010
GFINBURO	MXX	0.21	2000	AXTELCPO	GFAMSAA	0.21	2010
GFNORTEO	MXX	0.23	2000	GCARSOA1	GFINBURO	0.26	2010
MXX	SORIANAB	0.26	2000	GFNORTEO	MXX	0.21	2010
ARA	GFNORTEO	0.26	2001	GMEXICOB	MXX	0.29	2010
AZTECACPO	ELEKTRA	0.2	2001	KUOA	LIVEPOL1	0.22	2010
CEMEXCPO	FEMSAUBD	0.3	2001	ALFAA	MXX	0.22	2011
ALFAA	MXX	0.2	2002	CEMEXCPO	ICA	0.28	2011
AZTECACPO	ELEKTRA	0.22	2002	CEMEXCPO	MXX	0.21	2011
GFINBURO	MXX	0.29	2002	ELEKTRA	MXX	0.22	2011
GFNORTEO	MXX	0.26	2002	GFNORTEO	MXX	0.3	2011
ARA	MXX	0.22	2003	GMEXICOB	MXX	0.29	2011
GFINBURO	MXX	0.21	2003	HOMEX	URBI	0.28	2011
MXX	SORIANAB	0.26	2003	CEMEXCPO	MXX	0.27	2012
ALFAA	MXX	0.29	2004	FEMSAUBD	MXX	0.28	2012
AMXA	CEMEXCPO	-0.23	2004	GMEXICOB	MXX	0.26	2012
AZTECACPO	MXX	0.2	2004	MXX	WALMEX	0.22	2012
BIMBOA	MXX	0.2	2004	ALFAA	MXX	0.29	2013
GFINBURO	MXX	0.23	2004	AMXA	FEMSAUBD	-0.22	2013
GMEXICOB	MXX	0.26	2004	CEMEXCPO	MXX	0.22	2013
MXX	SORIANAB	0.24	2004	GFNORTEO	MXX	0.26	2013
ALFAA	MXX	0.23	2005	GMEXICOB	PE&OLES	0.22	2013
AMXA	MXX	0.23	2005	HOMEX	SAREB	0.22	2013
ARA	URBI	0.21	2005	ALFAA	MXX	0.24	2014
FEMSAUBD	MXX	0.22	2005	ALSEA	CULTIBAB	0.24	2014
GMEXICOB	MXX	0.25	2005	ASURB	GAPB	0.2	2014
KIMBERA	MXX	0.21	2005	GFNORTEO	MXX	0.26	2014
ALFAA	MXX	0.22	2006	GMEXICOB	MXX	0.29	2014
AMXA	MXX	0.25	2006	MFRISCOA-1	PE&OLES	0.27	2014
GFINBURO	MXX	0.28	2006	ALFAA	MXX	0.22	2015
GFNORTEO	MXX	0.2	2006	GFINBURO	MXX	0.22	2015
MXX	PINFRA	0.21	2006	AC	BIMBOA	0.2	2016
BIMBOA	MXX	0.23	2007	ALFAA	AZTECACPO	0.27	2016
GFNORTEO	MXX	0.23	2007	ALFAA	GFINBURO	0.22	2016
HOMEX	MXX	0.2	2007	ASURB	GAPB	0.21	2016
ICA	MXX	0.24	2007	GENTERA	PINFRA	0.23	2016
MXX	URBI	0.25	2007	GMEXICOB	MXX	0.21	2016
ALFAA	ARA	0.3	2008	MFRISCOA-1	PE&OLES	0.27	2016
ALFAA	AXTELCPO	0.26	2008	MXX	ORBIA	0.2	2016
AMXA	MXX	0.23	2008	ALFAA	ALPEKA	0.24	2017
CEMEXCPO	MXX	0.26	2008	CEMEXCPO	GFNORTEO	-0.24	2017
FEMSAUBD	MXX	0.26	2008	GAPB	OMAB	0.2	2017
GCARSOA1	MXX	0.21	2008	GFNORTEO	GMEXICOB	-0.22	2017
GMEXICOB	MXX	0.28	2008	HOMEX	URBI	0.28	2017
HOMEX	MXX	0.2	2008	MXX	WALMEX	0.23	2017
MXX	PE&OLES	0.25	2008	CEMEXCPO	FEMSAUBD	-0.27	2018
AMXA	MXX	0.21	2009	FEMSAUBD	GFNORTEO	-0.26	2018
AMXA	RCENTROA	0.21	2009	GAPB	OMAB	0.22	2018
BIMBOA	MXX	0.22	2009	GMEXICOB	MXX	0.26	2018
FEMSAUBD	MXX	0.21	2009	ICHB	SIMECB	0.22	2018
GCARSOA1	MXX	0.22	2009	GFNORTEO	GMEXICOB	-0.26	2019
GMEXICOB	MXX	0.26	2009	HCITY	UNIFINA	0.2	2019
HOMEX	MXX	0.25	2009				

In Tables 4 and 5 we provide information concerning most persistent links in the observed period.

**Table 4:** Persistent links in the rank (0.1, 1] for the period 2000-2009 (1/2).

	2000	2001	2002	2003	2004	2005	2006	2007	2008	2009
ALFAA-MXX	0.17		0.20		0.29	0.23	0.22			
AMXA-MXX	0.16		0.37	0.59	0.72	0.23	0.25		0.23	0.21
CEMEXCPO-MXX	0.27	0.14	0.38	0.36	0.49	0.44	0.49	0.50	0.26	0.37
FEMSAUBD-MXX	0.40	0.18	0.45	0.35	0.19	0.22	0.41	0.19	0.26	0.21
GFINBURO-MXX	0.21	0.16	0.29	0.21	0.23		0.28			0.17
GFNORTEO-MXX	0.23		0.26				0.20	0.23		0.20
BIMBOA-MXX		0.11	0.17		0.20	0.17		0.23	0.14	0.22
MXX-WALMEX				0.73	0.43	0.40	0.77	0.56	0.37	0.35
GMEXICOB-MXX					0.26	0.25	0.34	0.31	0.28	0.26
ASURB-GAPB									0.17	0.13
ICHB-SIMECB									0.41	0.34

**Table 5:** Persistent links in the rank (0.1, 1] for the period 2010-2019 (2/2).

	2010	2011	2012	2013	2014	2015	2016	2017	2018	2019
ALFAA-MXX		0.22	0.20	0.29	0.24	0.22				
AMXA-MXX			0.12	0.83						
CEMEXCPO-MXX	0.35	0.21	0.27	0.22	0.33	0.36	0.45	0.75	0.74	0.39
FEMSAUBD-MXX		0.35	0.28	0.42	0.56	0.42	0.59	0.42	0.87	0.89
GFINBURO-MXX	0.14					0.22				0.13
GFNORTEO-MXX	0.21	0.30	0.19	0.26	0.26	0.37	0.34	0.47	0.65	0.81
BIMBOA-MXX	0.18				0.16	0.18	0.15	0.13	0.18	
MXX-WALMEX	0.36	0.33	0.22	0.31	0.20	0.19	0.35	0.23	0.41	0.56
GMEXICOB-MXX	0.29	0.29	0.26	0.48	0.29	0.33	0.21	0.30	0.26	0.55
ASURB-GAPB	0.24	0.17	0.16	0.16	0.20	0.36	0.21	0.33	0.32	0.31
ICHB-SIMECB	0.34		0.44	0.34	0.11	0.36		0.37	0.22	

**Fig 4:**
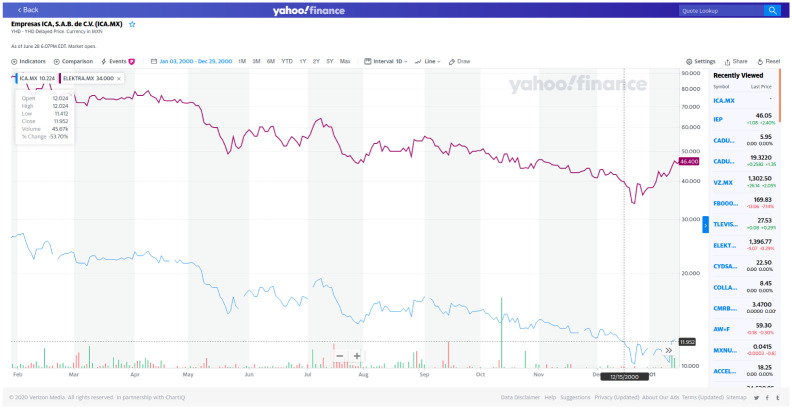
In purple the time series for ELEKTRA and in blue the time series for ICA. Prices in logarithmic scale for the year 2000. Source: Yahoo.Finance.

We obtain the following stylized facts:

- First of all we see in Fig 3 a stable continuous evolution of partial-correlations interdependency structure. At this stage of initial visual inspection, if an impact of global crisis episodes indeed existed (e.g., dot.com bubble, the subprime crisis and the European soveregin debt crisis) it doesn’t seem to produce large variations in network interdependency structures.
- There are several edges with a weight (partial-correlation) above the threshold 0.2 which frequently include the main index from the Mexican stock Exchange BMV denominated IPC (quoted as MXX in Yahoo.Finance); see the Tables 2 and 3.
- As we move forward in time, the market grows (with more nodes of stocks consistently quoted by year). However, it does not seem to be evidence that interconnectedness in the market changes drastically from one year to the next, even for the subprime crisis period.
- Connections must be due to exogenous factors to the market, but inherent to each stock, since the graph is based on partial correlations. However, for edges that include the IPC node, the other node may be a stock used in the construction of the index.
- A large number of links between stocks in different sectors wich is an empirical fact reported for other markets; see e.g., [16], and to our best knowledge, not previously documented for the Mexican stock market. Nonetheless, intrasectorial partial-correlations are also present.
- There are persistent links between pairs of stocks that appear frequently, but not systematically, over the twenty year period; see Tables 4 and 5.
- Negative partial-correlations appear only seldomly.
- For the year 2000 we see a partial correlation of 0.98 between ICA and ELEKTRA which appears to be an odd finding, but it is actually supported by data; see Fig 4.
- The strongest links above 0.3 are those typically having as one of its nodes the IPC. Also for the rank [0.2, 0.3] links with IPC as a node dominate but with a small decline in frequency in contrast with the interval (0.3, 1].
- FEMSA indeed has a persistent relationship with the IPC index with partial correlations above 0.3; see Tables 4 and 5. In this same tables we do not see an important stock such as AMX. This is an interesting confirmation of the strength of the GGm, since it captures real facts (see for example news stories from the mexican magazines expansion and el economista).

**Fig 5:**
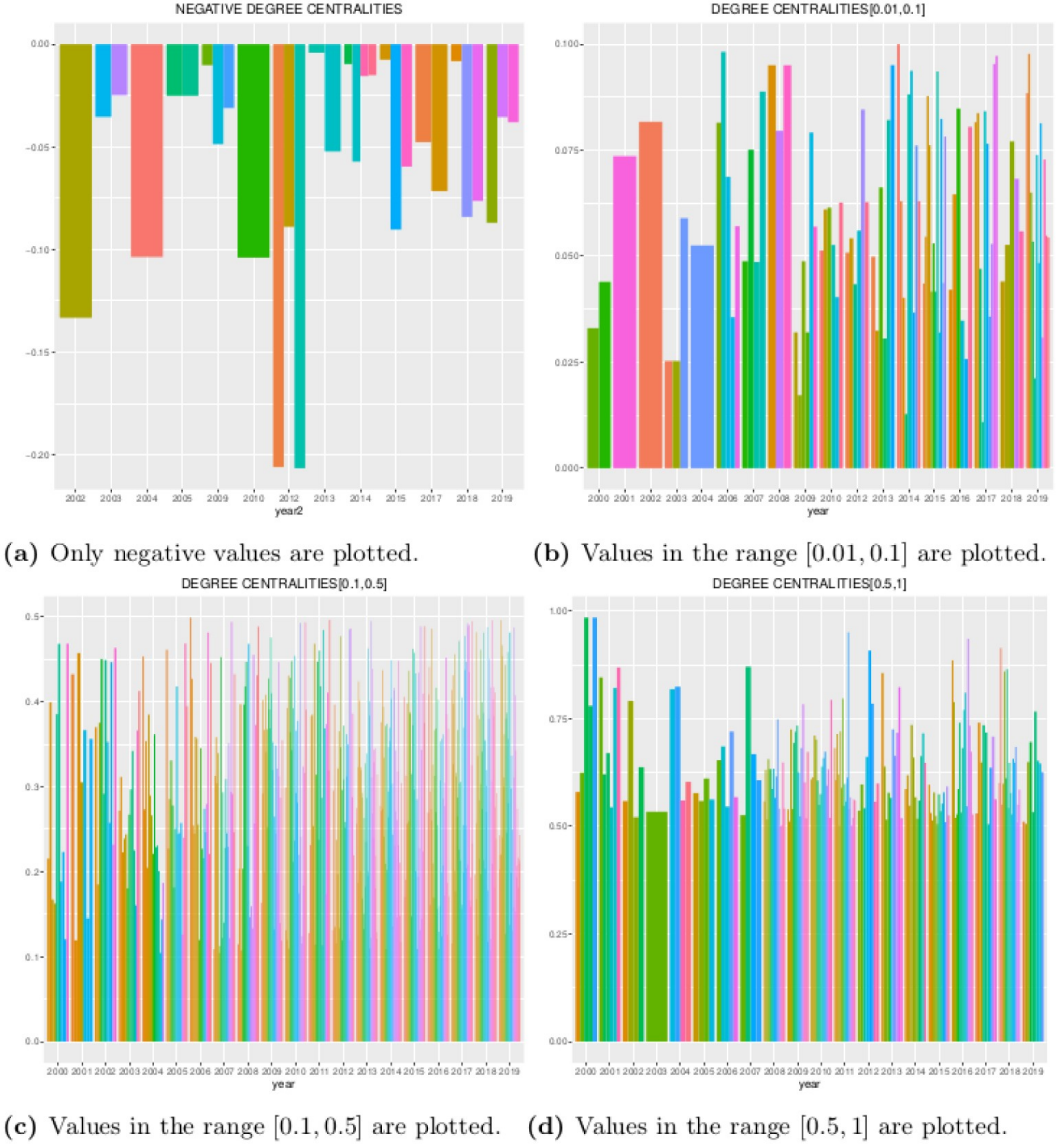
A comparison of degree centralities by year at different ranges.

In Fig 5 we see a panel of barplots for degree-centralities separated into different ranges for all stocks in their respective period. Links with negative values are few in quantity and magnitude as more precisely illustrated in Fig 5(a). In Fig 5(b) we see a quite homogenous distribution in the range [0.01, 0.1]. An analogous situation is appreciated in Fig 5(c) in the interval [0.1, 0.5]. Only in the range [0.5, 1] we see in Fig 5(d) a more heterogenous situation with some dominating stocks.

Before we continue with a discussion of results in this section, we estimate metrics (centralities) from network theory to see a possible effect of global financial crisis.

### Centralities from partial correlations

Centrality is a metric designed in such a way that a vertex with high centrality can be considered highly influential. The first concept of centrality we use is the *degree-centrality* which for a vertex in a weighted network is the sum of weights of the connecting edges. For our graphs of partial correlations, the degree centrality gives information on the pattern of a shock’s transmission. The idea is that if an influential (i.e., with high centrality) stock in the financial network is having a bad day, it will be accompanied by many other stocks in similar situations. *Note that there is no causality claimed here*. The second measure of centrality that we estimate is the *eigen-centrality*. This is a global measure in that scores for each node are assigned via a comparison of the quality of its links. For example a node with just one link to another influential node could have a highest eigen-centrality than a node with two or more links. The computation of eigencentralities transfers to a spectral analysis of the adjacency matrix and in crucial steps is substantiated by Perron-Frobenius theory (see e.g., [57, Chapter 17]). The third concept that we estimate is *betweenness-centrality* for the absolute values of weights. For each vertex, it gives the proportion of shortest paths passing through it.

#### Shock transmissions

Let us explain in more detail eigencentrality and at the same time also clarify shock transmissions. Let *V* = {1, …, *n*} index our set of stocks and recall the matrix **P** defined in (4). The eigencentrality is a function f:V→R satisfying

$$\begin{array}{c} f\left(v\right)=r\sum_{w\in N(v)}P_{v,w}f\left(w\right),v\in V, \end{array}$$ (5)

where *r* is a non negative constant and *N*(*v*) denotes the neighbors of *v*. Note that

$$\begin{array}{c} f\left(v\right)=r\sum_{w\in V}P_{v,w}f\left(w\right), \end{array}$$

since by definition *w* ∈ *N*(*v*) if and only if **P**_*v,w*_ ≠ 0. Now this can be written in matricial notation as

$$\begin{array}{c} f\left(V\right)=rPf{\left(V\right)}^{T}, \end{array}$$

where *f*(*V*) = (*f*(1), …, *f*(*n*)). Hence, *f*(*V*) is an eigenvector of **P** attached to *r* as its eigenvalue.

To continue we follow the discussion in [17], returning to the coefficients *β*_*i*,*j*_ in Eq (3). The matrix of coefficients **B** = (*β*_*i,j*_) with *β*_*ii*_ = 0 is then connected to the adjacency matrix as $B=diag{\left(J\right)}^{-\frac{1}{2}}Pdiag{\left(J\right)}^{\frac{1}{2}}$. We can write the linear regression in a compact matricial notation as

$$\begin{array}{c} \vec{R}-\mu =B\left(\vec{R}-\mu \right)+\epsilon =diag{\left(J\right)}^{-\frac{1}{2}}Pdiag{\left(J\right)}^{\frac{1}{2}}\left(\vec{R}-\mu \right)+\epsilon . \end{array}$$ (6)

Let $\tilde{R}\left(i\right)≔R\left(i\right)-\mu \left(i\right)$ and $\tilde{R}=\left(R\left(1\right)-\mu \left(1\right),\ldots ,R\left(n\right)-\mu \left(n\right)\right)$. Then,

$$\begin{array}{c} diag{\left(J\right)}^{\frac{1}{2}}\tilde{R}=Pdiag{\left(J\right)}^{\frac{1}{2}}\left(\vec{R}-\mu \right)+diag{\left(J\right)}^{\frac{1}{2}}\epsilon . \end{array}$$

Hence the vector $\vec{X}≔diag{\left(J\right)}^{\frac{1}{2}}\tilde{R}$ satisfies

$$\begin{array}{c} \vec{X}=P\vec{X}+\eta \end{array}$$

where $\eta ≔diag{\left(J\right)}^{\frac{1}{2}}\epsilon$.

Now assume that between times *t*_0_ and *t*_1_ there is a shock Δ = (0, …, 0, *δ*, 0, …, 0) affecting *X*(*i*). Then, $\vec{X}$ at time *t*_1_ is given by $P(\vec{X}+\Delta )+\eta$ and the change is then **P**Δ. Note that **P**Δ does not need to be a scalar of Δ, meaning that the shock originally affecting *X*(*i*) has also an impact on other components, indicating that the shock propagates.

The spectral decomposition of **P** helps in assessing the scale of propagation and rationalizes the definition of eigencentrality. Let *W*_1_, …, *W*_*n*_ be the set of eigenvectors of **P** and Λ = {λ_1_, …, λ_*n*_} the corresponding set of eigenvalues, which we assume is decreasingly ordered with respect to its modulus. Here is a common assumption: there is a unique eigenvalue attaining the spectral radius. This means |λ_1_|>|λ_2_|≥|λ_2_|… ≥ |λ_*n*_|. If the matrix **P** has only non-negative components, then the Perron-Frobenious theory guarantees we are in this situation and other properties besides; see e.g., [57, Chapter 17]. Represent Δ by Δ = ∑_*i*_ *α*_*i*_ *W*_*i*_. Then, for *k* ∈ **N**

$$\begin{array}{c} P^{k}\Delta =\lambda_{1}^{k}\{\alpha_{1}W_{1}+\sum_{i=2}^{n}{\left(\frac{\lambda_{i}}{\lambda_{1}}\right)}^{k}W_{i}\}. \end{array}$$

Hence

$$\begin{array}{c} \lim_{k\to \infty }\frac{1}{\lambda_{1}^{k}}P^{k}\Delta =\alpha_{1}W_{1}. \end{array}$$

Then, as time passes the leading term indicating the effect of the initial shock Δ takes the form $\lambda_{1}^{k}\alpha_{1}W_{1}$.

#### Results of estimation

**Fig 6:**
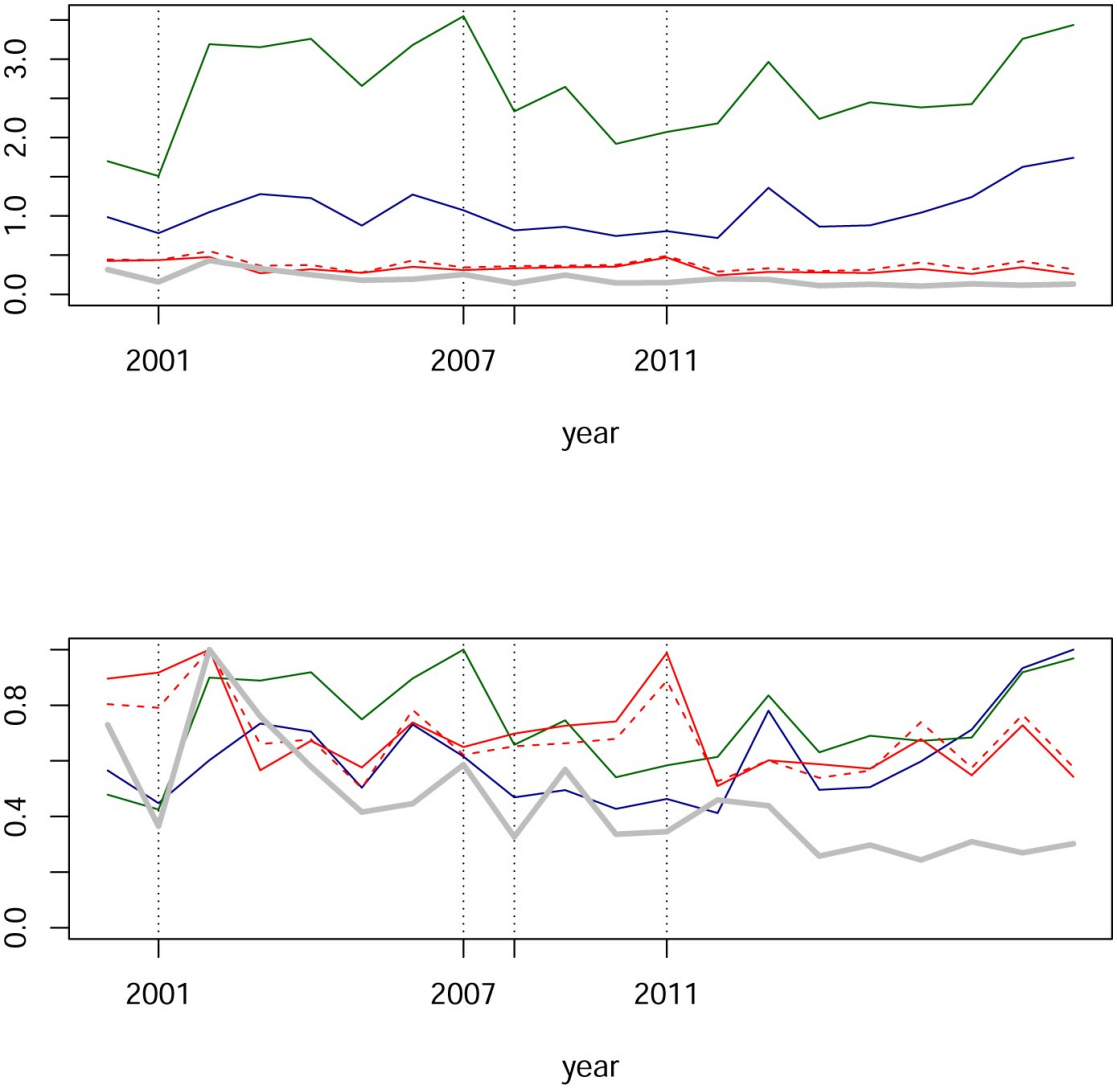
Network centralities from partial correlations. The blue line is the largest eigenvalue. The green line is the maximum degree-centrality by year, always attained by the IPC index. The red line is the average of degree-centralities, respectively the red dashed line is the average of absolute value of degree-centralities. The gray line represents the maximum betweenness-centrality of absolute values. In the upper panel, time series are shown in their original scale, while in the lower panel time series have been rescaled by its maximum.

In Fig 6 we see estimated centralities for our networks. The blue line is the largest modulus per year of eigenvalues. The green line represents the maximum degree-centrality for each year and unsurprising this maximum is always attained by the IPC index. The red (respectively red and dashed) line represents the average of each node’s degree-centrality (respectively the average of each node’s absolute value degree-centrality). The gray line represents the maximum of betweenness-centrality which has been computed for absolute values of weights with the R package igraph.

**Fig 7:**
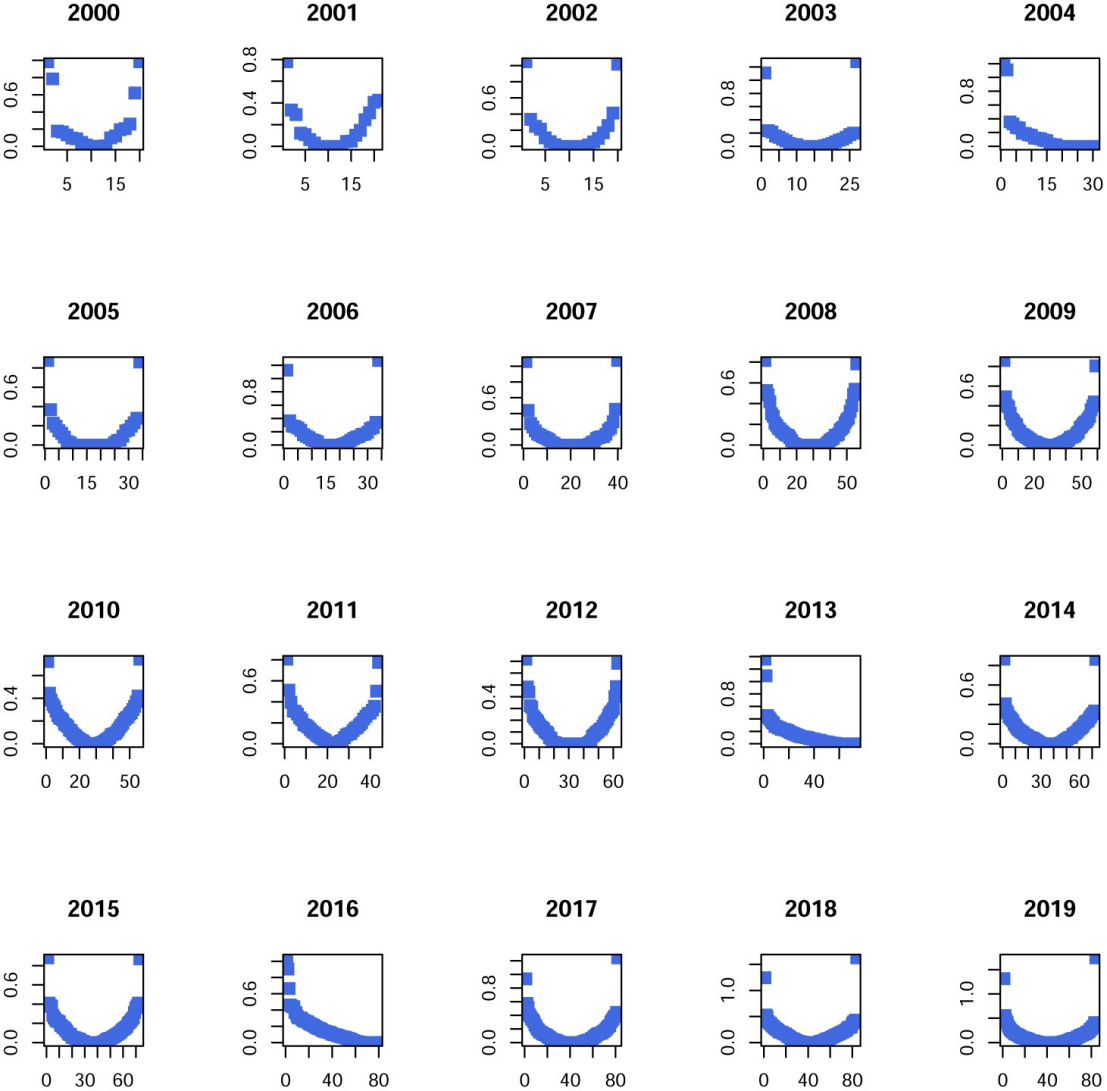
Eigenvalue modulus per year.

These are the findings we observe from Fig 6:

- The spectral radius is approximately bounded by two, which coincides with the range documented for other markets; see e.g., [16].
- The red line and the red dashed lined are almost indistinguishable. This happens as a consequence of the fact that almost all partial-correlations are non-negative. We also observe the stability on the metric represented by this line.
- The patterns of the green and blue lines are similar. As we mentioned, the green line is attained by the IPC index, so it could be expected that the blue line is also related to this index. Although we do not investigate this claim, assuming it is correct, in order to capture effects beyond the IPC index it might be necessary in this case to complement with the second eigenvalue together with its eigenvector for centrality and the analysis for a shock contagion. Indeed, Fig 7 shows that in many cases the dominant eigenvalue has a multiplicity of two or more, and in other cases that the second eigenvalue turns out to be close to the first. Certainly, the idea of considering beyond the dominant eigenvector for eigencentrality is not new; see [58]. Analysis for the Mexican case will be addressed elsewhere.
- There is indeed variability for centralities, but changes from one year to the next are indeed relatively small. Thus, changes are subtle. For example, for the subprime crisis period, we see a small upwards jump from 2005 to 2006 of around 0.4 and then from 2007 to 2008 a downwards jump of around 0.25. Small jumps are also observed for max degree-centrality on the green line.
- Continuing with the previous point, we see an abrupt upwards movement of the green line which we assume is associated with the dot.com bubble’s crisis: From the year 2001 to 2002.

#### Discussion

The financial networks of partial correlations illustrated in Fig 3 present low degree centrality and are sparse. Below we construct financial networks based on Pearson correlation matrices and we will also compute their centralities; see Fig 12. A comparison of Figs 6 and 12 yield evidence that partial-correlations generate sparser networks, understood by the fact that comparing year by year, centralities for partial correlations are significantly lower than for Pearson correlations. This was expected and agrees with the findings in [16] and [17] which also compare networks based on Pearson and partial-correlations. Interestingly, estimations of matrices are done by different methods and different data, thus, providing evidence that sparsity of partial-correlation based networks are robust with respect to statistical procedures and data. Another paper that also compares networks based on Pearson and partial correlations is [4]. The authors conclude that networks based on Pearson correlations show different structures than partial correlation matrices, and in particular a different clustering structure. However, they construct Minimum Spanning Trees and the comparison of sparsity is unclear. They also compute betweenness-centrality. Although they report differences, these are not as marked as the ones presented here. We will elaborate on betweenness-centrality further after we also analyze Tail-dependence networks.

The IPC index has been found to be a vertex where edges consistently present their highest weight (partial-correlation). Indeed, the maximum of degree- and eigen- centralities are always attained at this vertex. If the analysis of the mean variance portfolio of [23] holds true also for partial correlations, then this would mean that in such a portfolio, the IPC seen as an asset on its own would receive a lower weight. Thus, any Exchange-Traded Fund (ETF) tracking the IPC index does not diversify investments from the point of view of the classical Markowitz portfolio theory and achieve a lower proportion of portfolio value. Whether the negative relationship found in [23] also holds true for partial correlations can be the subject of future research, however we find that the IPC index also has high degree- and eigen- centralities for networks based on Tail-dependence and Pearson correlation matrices.

In the period 2000-2019 there are three important financial episodes: The dot.com bubble, the subprime crisis and the European sovereign debt crisis. The time series of centralities in Fig 6 exhibit several local maximum which may connect with those episodes. Now here is a trade-off. Partial correlations and the lasso estimation of the GGm indeed result in a stringent sieve in which only the most significant and “clear” relationships pass through and as a consequence the centrality time series are quite stable. However, the aforementioned financial episodes are captured by centralities of partial-correlations and show moderate increases. We will see that Tail-dependence networks exhibit a more sensible topology for those market conditions. This is reasonable due to the symmetric nature of distributions in GGm while on Tail-dependence networks the emphasis is on lower tails.

### Tail-dependence networks

For two random variables *X* and *Y* the Tail-dependence coefficient ([2]) is the limit

$$\begin{array}{c} \lambda_{L}=\lim_{q\to 0}P(X\leq F_{X}^{-1}\left(q\right)|Y\leq F_{Y}^{-1}\left(q\right)) \end{array}$$

where *F*_*X*_ represents the cumulative distribution of *X*, and similarly for *F*_*Y*_. It is clear that λ_*L*_ quantifies the relationship of lower tails between *X* and *Y*. In this section we focus on networks based on matrices whose components are the coefficients λ_*L*_ for pairs of stock time series. We estimate the Tail-dependence coefficient through the non-parametric estimator in [7] which is implemented in the R package FRAPO (Financial Risk Modelling and Portfolio Optimisation).

**Fig 8:**
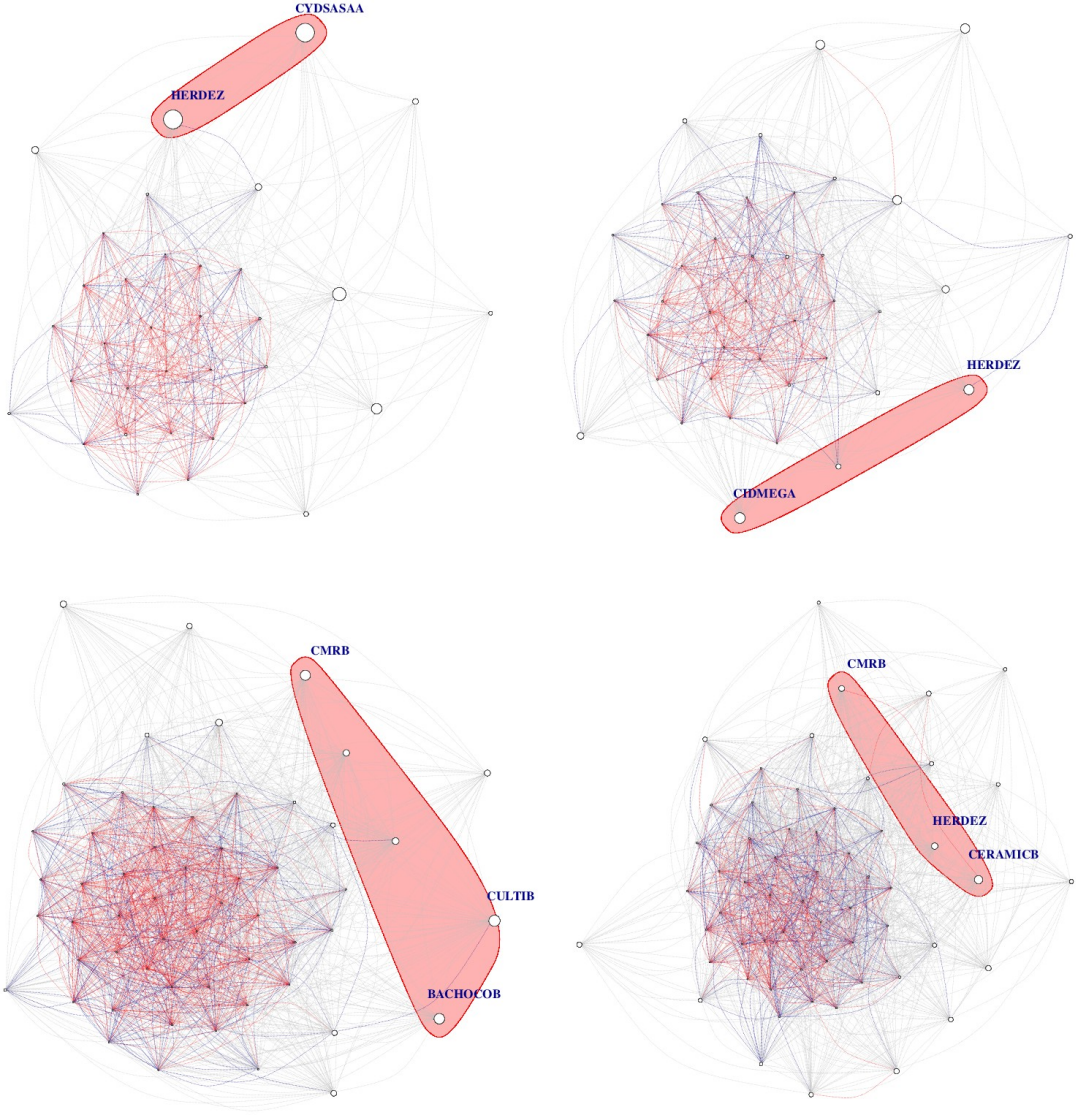
Tail-dependence networks for the years 2006-2009. Vertex size as a function of **betweenness-centrality**. The pink shaded area shows the highest 95% quantile. The colors of the edges are blue for weights in absolute value in the interval [0.2, 0.3], and red in the interval (.3, 1]. Otherwise they are gray.

**Fig 9:**
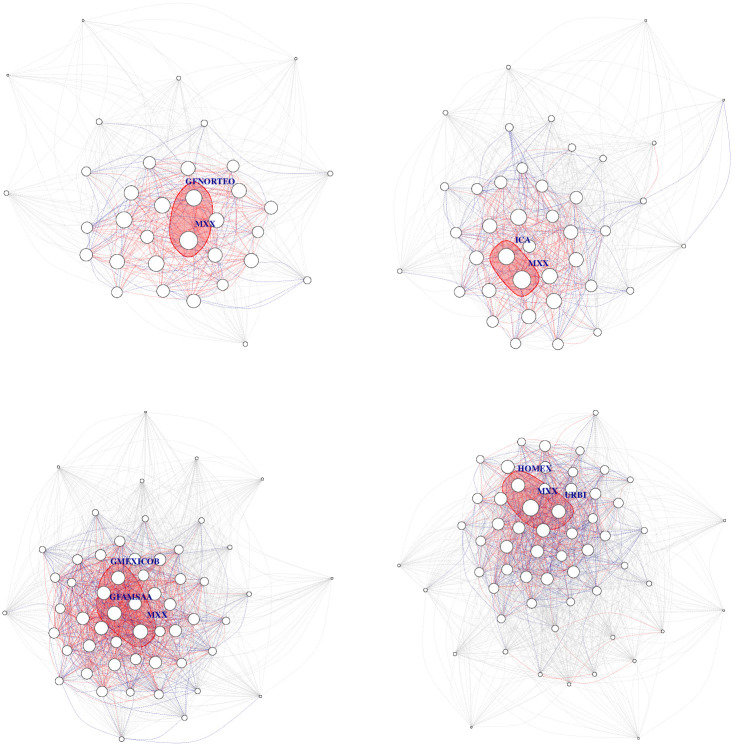
Tail-dependence networks for the years 2006-2009. Vertex size as a function of **eigen-centrality**. The colors of the edges are blue for weights in absolute value in the interval [0.2, 0.3], and red in the interval (.3, 1]. Otherwise they are gray.

**Fig 10:**
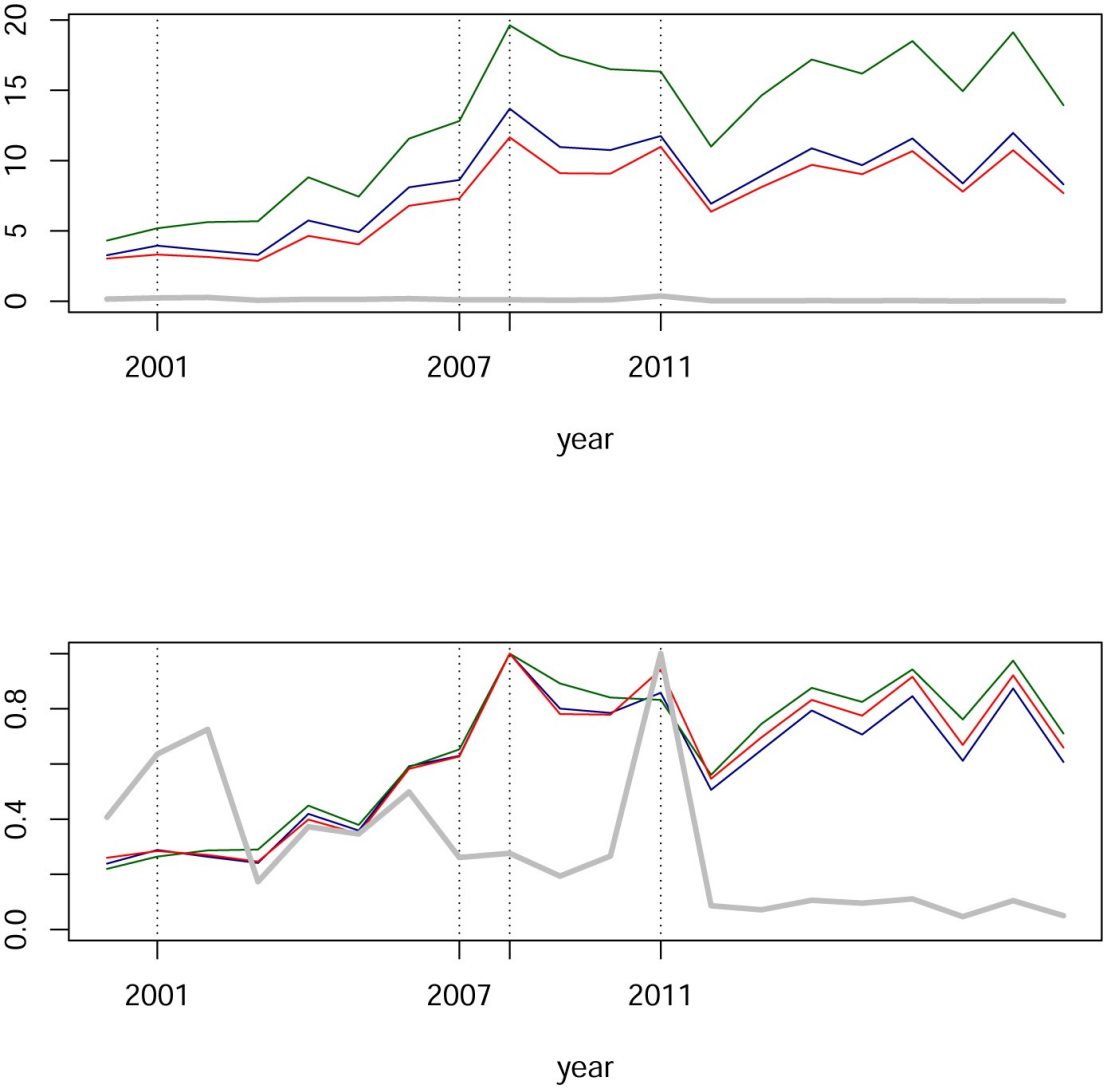
Network centralities from Tail-dependence networks. The blue line is the largest eigenvalue. The green line is the maximum degree-centrality by year. The red line is an average of degree-centrality. The gray line represents the maximum betweenness-centrality of absolute value weights. In the upper panel, time series are shown in their original scales, while in the lower panel time series have been rescaled by their maximums.

In Figs 8 and 9 the Tail-dependence networks for years during the period 2006-2009 are illustrated. In Fig 8 vertex size is a function of betweenness-centrality while on Fig 9 it is a function of eigen-centrality. The illustrated structure repeats for the years over the period 2000-2019. The time series of centralities are illustrated in Fig 10. This is a list of stylized facts:

1. The IPC index is also important with respect to eigen-centrality, just as it was for partial-correlation networks.
2. For betweenness-centrality the 95% quantile is quite dynamic concentrated in a few stocks and consistently does not include the IPC index.
3. The 95% quantile is more distributed for eigen-centrality than for betweenness-centrality.
4. Fig 10 clearly shows that time series of centralities experience an increase in activity associated with the subprime and the European sovereign debt crisis.

#### Discussion

In Fig 10 we see an illustration of financial network’s interdependency-evolution for the Mexican stock exchange. The first fact to note are the peaks in the years 2008 and 2011. It is reasonable to associate such increments in interconnectedness (as measured by degree- and eigen-centrality) to the subprime financial crisis and the European sovereign debt crisis. This is coherent with findings in the literature. It is worth emphasizing that in this literature, other data have been analyzed with different methods. For comparison, let us recall a few papers in this regard. A financial network from 100 selected stocks of financial institutions in the US is studied in [29]. Emphasis is on tail events from the point of view of systemic risk. They find that the banking sector is at the core of systemic risk between 2008 and 2010. In contrast, the insurance companies are less relevant for systemic risk. Their empirical results exhibit growing interconnectedness during the period of a financial crisis. A financial network based on Tail-distributions is studied by [30]. Data comes from a selection of 51 time series of large European banks and 17 sovereigns bonds during the period from 2006 through 2013. Their empirical results show that network densities increase from the intensity of the (subprime) financial crisis. More precisely, network densities increases from 2006 up to its (local) maximum around the peak of the global financial crisis and then decreases. A different approach based on entropy is studied by [28]. The empirical result reports that “node strengths peak in times of crisis”.

Hence, there exists an empirical fact manifest along a variety of methods applied to different data: estimated network-interconnectedness increases at some point in the development of a crisis, it might indeed affect one sector more than other, yet it is going to be globally observable. We evidence this empirical fact for the Mexican stock exchange in Fig 10. This is already interesting, and going deeper into the details, we mention two subtleties about timing and the different centralities. We have observed in partial-correlation networks that degree- and eigen-centrality show the same pattern. This similarity in patterns happens also for the Tail-dependence networks analyzed in this section; compare Figs 6 and 10. It will be observed again for networks based on (filtered) Pearson correlations; see Fig 12 below. However, betweenness-centrality exhibits a different pattern, more so for Tail-dependence networks; see [59] for a similar finding with different data and statistical estimation. A relevant difference worth emphasizing concerns the timing of local extrema. Whether this difference is indeed robust with respect to data and statistical procedures certainly is an interesting question for future research.

Differences in patterns, discernible in the time series, is also visible in the networks in Figs 8 and 9. In the former, in which node size is a function of betweenness-centrality, the most influential nodes are linked exclusively through gray edges. These nodes have a lot of links although all of them have small weights. On the latter graph, where node size is a function of eigen-centrality, the most influential nodes are connected together by red links. This indicates weights in the interval (.3, 1].

### Network theory: Community detection

#### DCC multivariate Garch model

Let $y≔{\left\{y_{t}\right\}}_{t=1}^{N}$ denote a one dimensional time series with *N* observations. A GARCH specification for its volatility usually starts with a flux of information determined by a filtration ${\left\{F_{t}\right\}}_{t=1}^{N}$ in which $F_{t}$ is a *σ*-algebra representing information at time *t* and *y* follows the dynamic

$$\begin{array}{c} y_{t}=E\left[y_{t}|F_{t-1}\right]+\epsilon_{t}\left(\theta \right). \end{array}$$

Here *θ* is a parameter vector whose definition specifies the model, while *μ*(*θ*) is the conditional mean of the time series at time *t*, usually modeled through an ARMA time series. For example an ARMA(1,1) (as we will consider here) is specified by

$$\begin{array}{c} \mu_{t}=\mu +\varepsilon_{t}+\phi \mu_{t-1}+\psi \varepsilon_{t-1}, \end{array}$$ (7)

where *ϕ*, *ψ* are parameters to be estimated and *ε* is white noise, i.e. an uncorrelated centered time series. The residual *ϵ*(*θ*) captures the conditional volatility of *y*:

$$\begin{array}{c} var\left(y_{t}|F_{t}\right)=E\left[{\left(y_{t}-\mu_{t}\left(\theta \right)\right)}^{2}|F_{t}\right]=E\left[{\left(\epsilon_{t}\left(\theta \right)\right)}^{2}|F_{t}\right]=var\left(\epsilon_{t}\left(\theta \right)\right). \end{array}$$

Its specification is the essence of a GARCH model. We will consider the standard GARCH(1,1) model:

$$\begin{array}{cc} \epsilon_{t} & =\sigma_{t}z_{t} \end{array}$$ (8)

$$\begin{array}{cc} \sigma_{t}^{2} & =\alpha_{0}+\alpha_{1}\epsilon_{t-1}^{2}+\beta_{1}\sigma_{t-1}^{2}, \end{array}$$ (9)

where ${\left\{z_{t}\right\}}_{t=1}^{N}$ is white noise.

Now consider a set of univariate time series *y*(1),…,*y*(*n*). A class of models in the *multivariate GARCH* literature known as Dynamic Conditional Correlation (DCC) was introduced by [60] and [61]. The DCC class builds upon univariate GARCH models and then specifies the dynamic of time varying conditional covariance matrix of the time series *y*(1),…,*y*(*n*). It has the general dynamics

$$\begin{array}{c} H_{t}=D_{t}R_{t}D_{t}. \end{array}$$

Here **D**_*t*_ is a diagonal matrix of time varying standard deviations from univariate GARCH models and **R**_*t*_ is a time varying correlation matrix. For estimation, the matrix **R**_*t*_ is decomposed as

$$\begin{array}{c} R_{t}={\left(Q_{t}^{*}\right)}^{-1}Q_{t}{\left(Q_{t}^{*}\right)}^{-1} \end{array}$$

where **Q** is specified in [62, Equation (2)].

#### Means for the years 2006 and 2008

In Table 6 we report the coefficient *μ* in the specification (7) for each stock in the year 2006, and analogously for the Table 7 in the year 2008. The estimation of these coefficients provides further support to the claim made after the visual evidence of Fig 1.

**Table 6:** The coefficient *μ* for the year 2006.

	Stock	mu value	Stock	mu value
1	ALFAA	0.0006	GISSAA	0.0011
2	ALSEA	0.0026	GMD	0.0036
3	AMXA	0.0017	GMEXICOB	0.0021
4	ARA	0.0027	HERDEZ	0.0016
5	AXTELCPO	0.0013	HOMEX	0.0027
6	AZTECACPO	0.0006	ICA	0.0026
7	BACHOCOB	0.0012	ICHB	0.0036
8	BIMBOA	0.0018	KIMBERA	0.0011
9	CEMEXCPO	0.0013	MXX	0.0021
10	CMOCTEZ	0.0016	PAPPEL	0.0018
11	CMRB	0.0013	PASAB	-0.0015
12	CYDSASAA	0.0016	PE&OLES	0.0027
13	ELEKTRA	0.0017	PINFRA	0.0065
14	FEMSAUBD	0.0024	RCENTROA	0.0039
15	GCC	0.0022	SORIANAB	0.0021
16	GFINBURO	0.0007	URBI	0.0018
17	GFNORTEO	0.0033	WALMEX	0.0023

**Table 7:** The coefficient *μ* for 2008 year.

	Stock	mu value	Stock	mu value	Stock	mu value
1	AC	-0.0013	ELEKTRA	0.0006	IDEALB-1	-0.0008
2	ALFAA	-0.0019	FEMSAUBD	0.0019	KIMBERA	0.0002
3	ALSEA	-0.0013	FINDEP	-0.0036	LAMOSA	-0.0016
4	AMXA	-0.0023	FRAGUAB	0.0004	MAXCOMA	-0.0028
5	ARA	-0.0018	GAPB	-0.0017	MEDICAB	-0.0001
6	ASURB	-0.0014	GCARSOA1	-0.0003	MEGACPO	-0.0028
7	AUTLANB	0.0037	GCC	-0.0032	MXX	-0.0010
8	AXTELCPO	-0.0053	GFAMSAA	-0.0023	OMAB	-0.0024
9	AZTECACPO	0.0000	GFINBURO	0.0008	PAPPEL	-0.0047
10	BACHOCOB	-0.0020	GFNORTEO	-0.0003	PASAB	-0.0030
11	BIMBOA	0.0002	GIGANTE	-0.0026	PE&OLES	-0.0010
12	CABLECPO	0.0000	GISSAA	-0.0009	PINFRA	-0.0016
13	CEMEXCPO	-0.0030	GMD	-0.0053	POCHTECB	-0.0048
14	CIEB	-0.0023	GMEXICOB	-0.0032	SAREB	-0.0033
15	CMOCTEZ	-0.0005	GRUMAB	-0.0001	SIMECB	0.0007
16	CMRB	-0.0005	HOMEX	0.0007	SORIANAB	0.0010
17	CULTIBAB	-0.0001	ICA	-0.0006	TMMA	-0.0040
18	CYDSASAA	-0.0029	ICHB	0.0010	URBI	-0.0018

#### Modularity

Assume we are given an undirected and unweighted graph *G* with vertexes *V* = {1, …, *n*} and edges *E*. Community structure in the graph means that there exists a partition of *V* in groups of vertices in such a way that within groups vertices are highly connected and more edges exist among them, while at the same time, edges between groups are less observed; see [40] for a survey of methods in community detection. The aforementioned description presents a general idea and to make it operative, it is necessary to use a more quantitative formulation. A popular approach is through the famous concept of modularity as introduced by [63] and further developed in [64]. Following the notation of [64] we introduce the following objects. Let **A** = (*A*_*i,j*_) be the adjacency matrix of *G* and let $m=\frac{1}{2}\sum_{i}k_{i}$, where *k*_*i*_ denotes the degree of vertex *i*, so *k*_*i*_ = ∑_*j*_ *A*_*i*,*j*_. Further denote by **s** ∈ {1, …, *n*}^*n*^ a vector having the same dimension as **A**, and representing an allocation of vertexes to communities. Thus, **s**_*i*_ represents the community assigned to vertex *i*. Now the idea is to compare the graph *G* with a graph *G*′ having no community structure. A group *V*_*k*_ = {*i* ∈ *V* ∣ **s**_*i*_ = *k*} possesses an accumulated weight of $\sum_{i,j\in V_{k}}A_{i,j}$. Now for *G*′, assuming it is a random instance of an Erdős-Rényi graph, the set *V*_*k*_ should have an accumulated weight of $\sum_{i,j\in V_{k}}\frac{k_{i}k_{j}}{2m}$. Hence, the difference $\sum_{i,j\in V_{k}}A_{i,j}-\frac{k_{i}k_{j}}{2m}$ quantifies how distant is the immersion of community *V*_*k*_ in the graph *G* from *G*′. The modularity function is defined as the sum of these differences over all communities:

$$\begin{array}{c} Q\left(s\right)≔\sum_{k}\sum_{i,j\in V_{k}}(A_{i,j}-\frac{k_{i}k_{j}}{2m})=\sum_{i,j\in V}(A_{i,j}-\frac{k_{i}k_{j}}{2m})\delta \left(s_{i},s_{j}\right), \end{array}$$

where *δ*(**s**_*i*_, **s**_*j*_) = 0 unless **s**_*i*_ = **s**_*j*_ in which case *δ*(**s**_*i*_, **s**_*j*_) = 1.

As such, the modularity function *Q*(⋅) is defined for unweighted, undirected graphs. In particular, for graphs obtained from a correlation matrix, which indeed is weighted, the modularity function *Q*(⋅) needs to be adjusted. Moreover, the null model (the graph *G*′) is critical for the well-functioning of modularity; see the discussion in [40]. Hence, to deal with this problem, we choose to work with the formulation of [36] where the correlation matrix is filtered and modularity is adjusted for the right “null model” *G*′. The analysis is again based on a spectral analysis as we now explain. Let **C** be a correlation matrix and consider the set of eigenvalues λ_1_, …, λ_*n*_ which we assume are displayed in increasing order. Let *v*_1_, …, *v*_*n*_ be the corresponding eigenvectors. Moreover, let *T* be the number of observations and λ_−_, λ_+_ be the critical values

$$\begin{array}{c} \lambda_{-}≔{\left(1-\sqrt{\frac{n}{T}}\right)}^{2},\;\lambda_{+}≔{\left(1+\sqrt{\frac{n}{T}}\right)}^{2}. \end{array}$$

The values λ_−_, λ_+_ are parameters for Marcenko-Pastur distribution in random matrix theory which is given by $\rho \left(\lambda \right)=\frac{T}{n}\frac{\sqrt{\left(\lambda_{+}-\lambda \right)\left(\lambda -\lambda_{+}\right)}}{2\pi \lambda }$. Let us introduce the matrices

$$\begin{array}{cc} C^{r} & ≔\sum_{\lambda_{i}\leq \lambda_{+}}\lambda_{i}v_{i}^{\text{tr}}\cdot v_{i} \end{array}$$ (10)

$$\begin{array}{cc} C^{g} & ≔\sum_{\lambda_{+}<\lambda_{i}<\lambda_{n}}\lambda_{i}v_{i}^{\text{tr}}\cdot v_{i} \end{array}$$ (11)

$$\begin{array}{cc} C^{m} & ≔\lambda_{n}v_{n}^{\text{tr}}\cdot v_{n}. \end{array}$$ (12)

We have a decomposition of the correlation matrix **C** given by

$$\begin{array}{c} C=C^{m}+C^{g}+C^{r}. \end{array}$$ (13)

From the ordering of the eigenvalues, the matrix **C**^*r*^ represents random noise, **C**^*m*^ a global signal which in our financial context is attached to the market as a whole and **C**^*g*^ represents information in a mesoscopic scale between **C**^*r*^ and **C**^*m*^. Next, we explain how the modularity function *Q*(⋅) is adjusted. Accordingly, focusing on the matrix **C**^*g*^, and taking into account the decomposition (13), the null model is **C**^*r*^ + **C**^*m*^ and the modularity function takes the form

$$\begin{array}{cc} Q_{3}\left(s\right) & ≔\frac{1}{C_{norm}}\sum_{i,j}\left[C_{i,j}-C_{i,j}^{r}-C_{i,j}^{m}\right]\delta \left(s_{i},s_{j}\right)\\ & =\frac{1}{C_{norm}}\sum_{i,j}C_{i,j}^{g}\delta \left(s_{i},s_{j}\right) \end{array}$$ (14)

for *C*_*norm*_ = ∑_*i*,*j*_ *C*_*i*,*j*_ a normalizing constant. However, the set of eigenvalues λ_*i*_ satisfying λ_+_ < λ_*i*_ < λ_*n*_ could be empty (as we will find for some years in our sample). In this case the matrix **C**^*g*^ will be undefined and it makes no sense to consider it. For those cases we will consider a decomposition **C** = **C**^*s*^ + **C**^*r*^ with $C^{s}≔\sum_{\lambda_{+}<\lambda_{i}}\lambda_{i}v_{i}^{\text{tr}}\cdot v_{i}$ and then the modularity is defined by

$$\begin{array}{cc} Q_{2}\left(s\right) & ≔\frac{1}{C_{norm}}\sum_{i,j}\left[C_{i,j}-C_{i,j}^{r}\right]\delta \left(s_{i},s_{j}\right)\\ & =\frac{1}{C_{norm}}\sum_{i,j}C_{i,j}^{s}\delta \left(s_{i},s_{j}\right). \end{array}$$ (15)

Hence, in this section we maximize the modularity functions *Q*_2_ and *Q*_3_ in order to define communities and report on them. It is known that the maximization of modularity functions is a *NP-hard* problem; see [65]. Consequently, the optimization is approached through several heuristic algorithms. We implement the popular Louvian algorithm, adjusted as described by [36] according to the modularity functions *Q*_2_ and *Q*_3_.

#### Modularity function *Q*_2_

**Fig 11:**
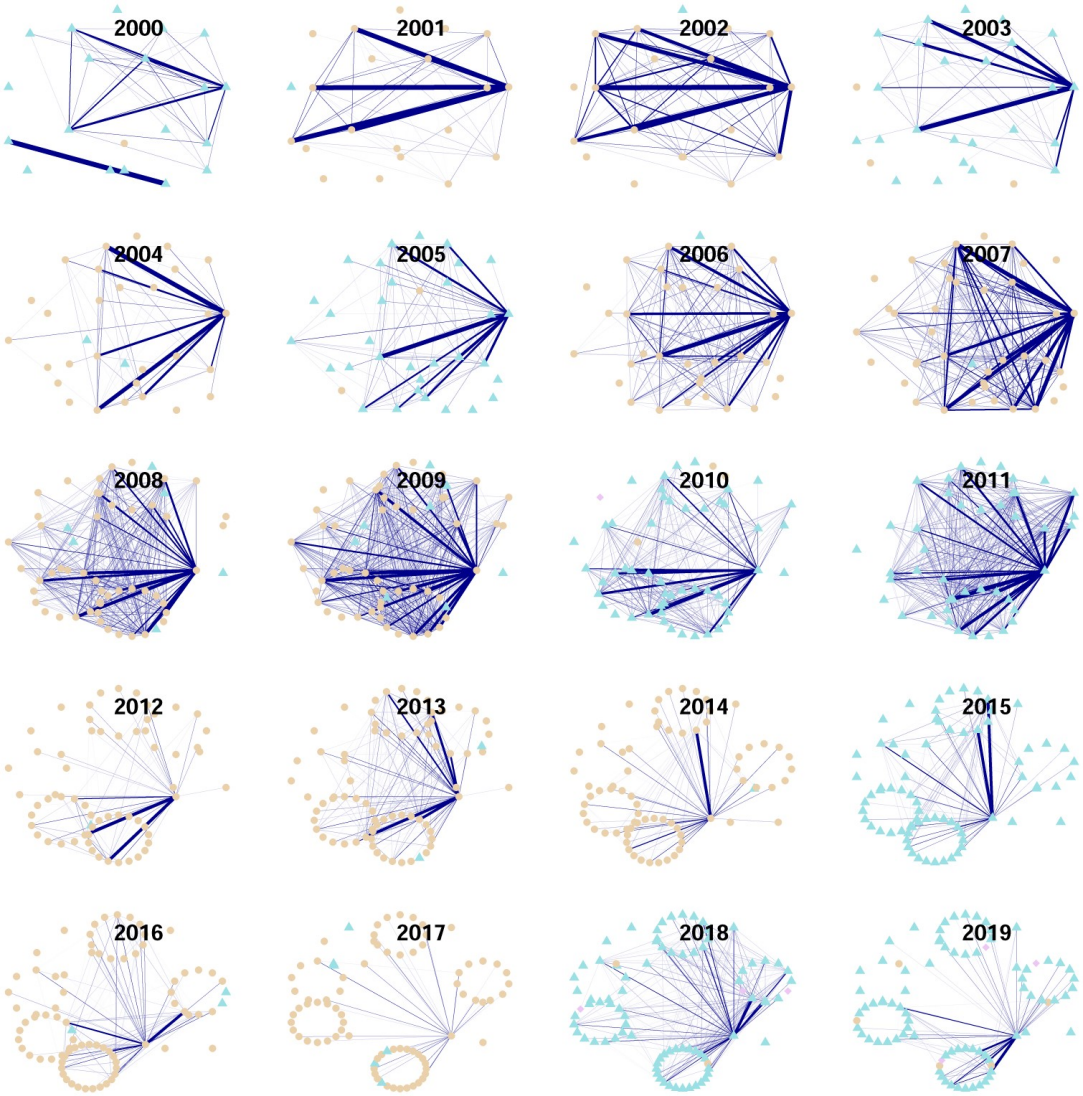
Communities obtained from modularity function *Q*_2_. The color of the nodes represent community, which is equivalently represented by the vertex’ shape. For clarity, only edges with weights in absolute value in the interval [.3, ∞) are shown. Only weights above 0.5 in absolute value are distinguished in the edge’s width.

**Fig 12:**
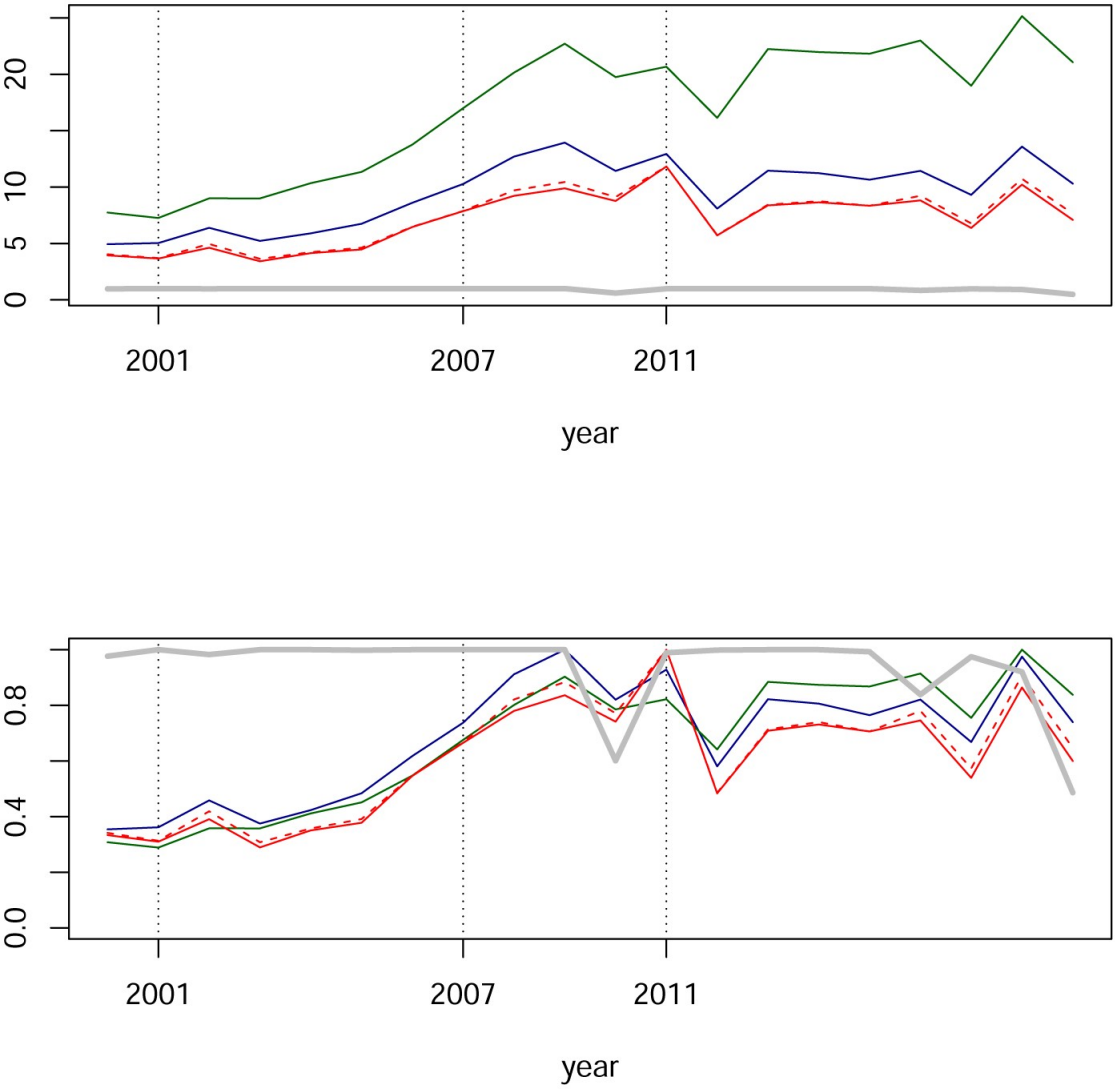
Network centralities from filtered Pearson networks based on the matrix C^*s*^. The blue line is the largest eigenvalue. The green line is the maximum degree-centrality by year. The red line is an average of degree-centrality. The gray line represents the maximum betweenness-centrality. In the upper panel, time series are shown in their original scale, while in the lower panel time series have been rescaled by its maximum.

In Fig 11 we see the resulting communities obtained with the Louvian algorithm applied to the modularity function *Q*_2_ defined in (15). During all the years of the period there are two communities. The first community is a “giant component” and the other community consists of a small number of isolated vertices. Hence, at this scale our procedure does not detect a complex community structure. This is unsurprising, since *Q*_2_ is based on the matrix **C**^*s*^ which includes the “market mode”. Note however the stylized fact:

- The turmoil at the subprime financial crisis and the European sovereign debt crisis periods are captured by a visually evident increase in interconnectedness. This can also be observed from the time series of centralities in Fig 12.

#### Modularity function *Q*_3_

**Fig 13:**
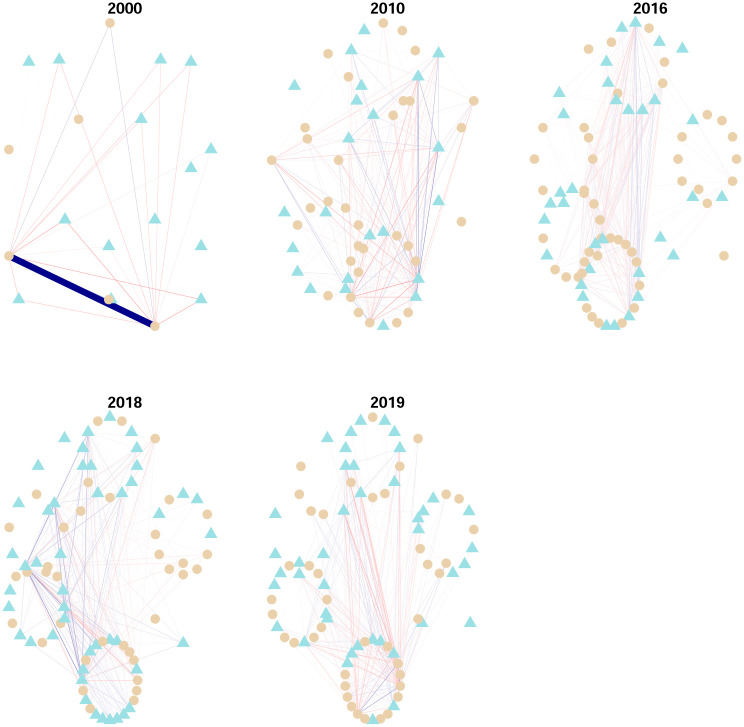
Communities from the modularity function *Q*_3_. The color of nodes identifies membership to the same community and equivalently for the vertex’ shape. For clarity, only the edges with weights in absolute value in the intervals [.05, ∞) are shown. Only weights above 0.5 in absolute value are distinguished via the edge’s width.

For the definition of the modularity function *Q*_3_ the matrix **C**^*g*^ is necessary and should not be a null matrix. For our data, this is the case for only a few years: 2000, 2010, 2016, 2018 and 2019. For them, a representation of communities can be seen from Fig 13. This is what we observe:

- First of all, in each year, there are only two communities as can be seen from the color of the vertexes, or equivalently from their shape. Interestingly, there is no clear larger community.
- Second, for our data the industrial sector is non determinant for the community assignment. More clearly, each industrial sector has vertexes in each community. This fact should be compared with the finding based on partial correlations where there also existed intersectorial links.
- This is our explanation for the years in which there existed a non-trivial matrix **C**^*g*^. First of all recall that this matrix represents structure between the scales of individual stocks and the market as a whole, while in crisis periods this last structure is what prevails since stocks tend to be highly correlated at those times. In the year 2000 the peak of the dot.com bubble is located and for the years 2001 and 2002 bearish markets prevailed. What we see from Fig 11 for the network constructed from the matrices **C**^*s*^, is an increase in interconnectedness, while in Fig 13, we see that in the period 2000:2002, there existed a mesoscopic structure for the year 2000, in which there is a “local minimum” for graph interconnectedness. The same occurs, analogously for the year 2010 in Fig 13, which coincides with a local minimum in Fig 11 for the “extended” subprime crisis period 2007-2010.
- Now we compare the years 2016, 2018 and 2019 in Figs 11 and 13. Those are years in which various global events occurred, some of them: The Brexit (starting from its referendum in june 2016), the US elections for the period 2017-2020, the China-US trade conflict starting from july 2018. However, none of these seems to be comparable to the magnitudes of the dot.com bubble and the subprime crisis. In particular for the Mexican stock market they didn’t have a sufficient impact to hide the effects of a mesoscopic structure, inducing all stocks to move as a result of a common factor.

#### Discussion

In this section we apply the methodology presented by [36]. The authors of that paper obtain the result that for stocks in the S&P500 index, the maximization of modularity without any sort of adjustment results in a unique community. This is uninteresting and also happens in our case with data from the Mexican stock exchange under the modularity function *Q*_2_. It is based on the Pearson correlation matrix after noise has been filtered out according to Random Matrix Theory. This is the matrix **C**^*s*^ which includes, as we mentioned before, the “market mode” from which the result of a unique community is unsurprising. In the paper [36] communities under *Q*_3_ are also determined. They obtain five communities for stocks in the S&P500 index. In our case there are only two communities, but it is also true that year by year we are considering on average less than one-fifth of the number of stocks in the S&P500 index. In this sense, magnitude in the number of communities seems to be coherent. The configuration of community-structure on both cases shares two properties: (a) communities are multisectorial and (b) negative links joining nodes belonging to different communities are mostly negative. This is already interesting for the objective of understanding the interdependency structure in a single trustable snapshot. It also provides information for applications. For example, the fact that inter-communities links are negative is a useful taxonomy for the tasks of portfolio-allocation and hedging.

## Conclusion

In global crisis periods, price levels of stocks in the Mexican stock exchange indeed present obvious changes which are visually evident and confirmed by econometric models. We have shown this fact here and it is also documented by other authors. However, the interdependency structure is a more complex phenomenon and much less studied. Our findings show that as long as partial-correlations are concerned, the interdependency structure is quite stable and centrality metrics from network theory have the sensibility to quantify small variations. Degree- and eigen-centralities indeed present variations, an upwards jump at the peak of the crisis and then a downwards jump when the shock of the crisis has been absorbed in the market. Another interesting finding from studying interdependency structure from partial-correlations is that only a small number of negative partial correlations which are also in magnitude small are present. We argue this is an indicator of a positive synergy of an integrated market. Reinforcing this claim, we find that industrial sectors are strongly interconnected even at the level of partial correlations. This is a less studied property, in general and in particular for the Mexican case.

Estimation of networks based on different matrices successfully captures different aspects of interdependency. Tail-dependence networks and their centralities maxima have been shown to give the most concise timing for the crisis’s heights (for the subprime and European debt crises).

Interdependency from the point of view of (“full”) correlations confirms findings from partial correlations. It also provides evidence of an integrated market for the Mexican case. Indeed, this is what we learned from the estimation of modularities which determined community structure without separating industrial sectors. From filtered matrices with noise filtered out (the matrices **C**^*s*^), a single giant component emerged. Moreover, here the effect of global episodes for interdependency structure was clear even by simple visual inspection. This is what we learned in Fig 11 and is perfect as evidence for the modeling strength. Indeed, correlations are more sensitive to trading activity than partial-correlations and capture relationships among stocks due to such activity which is even more pronounced at crisis periods. We also studied community structure from the matrices **C**^*g*^, which are the correlation matrices after noise and the global market mode have been filtered out. At this scale it happens that only a few observed years present a mesoscopic structure. For the years 2000 and 2010 in which mesoscopic structure is present, we observe a “local minimum” for interconnectedness in Fig 11. For the years 2016, 2018 and 2019 we also note a turmoil of stress periods (e.g., the Brexit,the US-China trade conflict, etc.) which nevertheless are not to be compared in severity with the episodes of the dot.com bubble and the subprime crisis. Hence they are not able to blur the presence of structure at the mesoscopic level.

## Supporting information

S1 Table List of analyzed stocks. (CSV)

S1 File (PDF)
