# Supplementary material for: The interdependency structure in the Mexican stock exchange: A network approach
Source: PLoS One. 2020 Oct 29;15(10):e0238731. doi: 10.1371/journal.pone.0238731 (PMC7595317; doi:10.1371/journal.pone.0238731)
Supplement: S1 File — (PDF) [file pone.0238731.s002.pdf]

# The interdependency structure in the Mexican stock exchange: A network approach

Erick Trevino Aguilar erick.trevino@im.unam.mx *

July 3, 2020

**Abstract**

Our goal in this paper is to study and characterize the interdependency structure of the Mexican Stock Exchange (mainly stocks from BMV) in the period 2000-2019 and provide visualizations which in a one shot provide a big-picture panorama. To this end, we estimate correlation/concentration matrices from different models and then compute centralities and modularity from network theory.

## 1 Introduction

In this paper we investigate the interdependency structure of daily returns in the Mexican stock exchange market. To this end, we build a database of free and publicly available time series of main stocks for the period 2000-2019 and conduct our study in stages that are then put together to give a unified treatment to our main topic of interest here which is the interdependency structure of daily log-returns in the Mexican stock exchange.

In the first stage we focus on the estimation of partial correlations of log returns of daily prices. The reason to focus on partial correlations is the following. Given a collection of Gaussian series $A_1, \dots, A_n$ a zero partial correlation between $A_1$ and $A_2$ implies that $A_1$ and $A_2$ are conditionally independent meaning that $A_1$ and $A_2$ could still be (unconditionally) correlated but only through a third factor adapted to the other series $A_3, \dots, A_n$. There are of course different methods to estimate a covariance/correlation/concentration matrix and we have selected a estimation based on a specific class of Markovian Random Fields (MRF) which in the statistical literature is well known under the name Gaussian Graphical models (GGm). The adjective "graphical" emphasizes the fact that attached to the probabilistic model there is a graph in which edges expresses conditional dependencies, from which a very convenient visual representation is obtained. There are three reasons to work with this model. First of all, the benefit of the already mentioned visual representation provided by the model. The second is that we have decided to study the period 2000-2019 in a yearly basis. There

*Unidad Cuernavaca del Instituto de Matemáticas, UNAM

is a trade-off to this treatment. On the one hand, short periods of time reduce problems with heavy tails. On the other, the number of stocks in each year is a significant proportion of the available observations. Hence, a *lasso*-regularized estimation is useful in this context which is inbuilt in the estimation of a GGm. Third, we want an estimation that filters out a "noisy" correlation selecting only clear relationships between two series, again this is provided by the *lasso*-regularized estimation. Loosely speaking, we follow a partial correlations selection approach which conceptually is comparable to a covariance selection approach [10]. Once partial correlations matrices have been estimated we provide a list of *stylized facts* from them. Then, taking the graphs constructed from the matrix of partial correlations as its adjacency matrix, we compute eigen-, between-, and degree- centralities.

In a second stage we study networks based on matrices whose every component is the Tail-dependence coefficient ([31]) of every pair of log returns. This coefficient quantifies the relationship of lower tails and captures dependencies in the events of negative returns.

In the third stage we estimate correlation matrices of time series (estimated through a Multivariate Dynamic Conditional Correlation GARCH specification). Then, apply a technique from network-theory based on those correlation matrices: The maximization of a modularity objective function. This procedure will provide with a partition on the stocks list for a community structure.

In our main goal of studying the interdependency structure of the Mexican stock exchange we contribute to the existing literature on financial networks in the following aspects. First of all, many papers focus on financial networks constructed from Pearson correlation matrices but much less papers focus on financial networks constructed from partial correlations and/or Tail-dependence matrices as we do here. Moreover, from the few papers focusing on partial correlation matrices, we are not aware of any of them applying the Gaussian Graphical model we consider in this paper. As a consequence no paper has previously compared network-structures from the three afore-mentioned different matrices (Pearson correlation, partial correlations and Tail-dependence) as we do in the present paper. As we demonstrate, this is interesting since depending on the underlying matrix, different network-topologies are unveiled, and through this differentiation one gains new information.

Many papers study aggregated financial indices and do not go into the details of analyzing at the level of stocks in the selected market. Hence, missing the point of analyzing interdependency at the level of individual stocks, where the network perspective could represent an advantage to support financial decisions; see e.g., [25, pp. 8, just before the section "Factor models"]. For example, we find that the main index IPC is "influential" with respect to degree- and eigen- centrality but the intensity varies with respect to which matrix is the network based on. In particular, the index does not convey every information in the market; compare e.g., again with [25, pp. 10, first paragraph].

Few papers focus in the case of Mexico, a representative market in the region which some studies have found to be a connecting node between Latin American and US markets; see [62]. Hence, playing a key role. For example, [27] is an early paper studying stock market integration between Latin American countries and the US. This includes

Mexico, but only as part of the block with no particular focus.

After this introduction the paper is organized in the following form. Section 2 gives some background on the approach of random networks in finance and economics. It also provides details on the data used to feed the models. In Section 3 we report on the estimated partial correlation matrices from GGm's. In Section 4 we report centralities of networks based on the estimated partial correlation matrices. In Section 5 we focus on networks based on Tail-dependence coeffficients. In Section 6 we report on correlation matrices computed from a multivariate GARCH specification and then maximize a modularity objective function of networks based on these matrices. This will define groups (communities) of stocks. Section 7 concludes the paper with a financial discussion based on the main findings of estimations.

# 2 Background

The classical Markowitz theory of portfolio selection illustrates the relevance of asset correlation matrices for financial decisions. However, it has been longly known the nontriviality of correlation estimation from empirical data; see e.g., [28]. Moreover, in contexts where sparse correlation (specially for partial correlation) matrices are expected, it is desirable to have a systematic method to discard "non-clear correlations" and account for a parsimonious model as motivated by [10]. As we mentioned in the introduction, in this paper we choose to apply a GGm for a parsimonious estimation of concentration/partial-correlation matrices. Estimation of Tail-dependence coefficients are based on the non-parametric estimator in [47]. Pearson-correlation matrices are estimated from a multivariate GARCH model.

Beyond the estimation problem, it is useful to have tools that starting from matrices are able to generate metrics providing snapshots of the market from which quick but trustable diagnosis are possible. Situations in which this is desirable include, from the point of view of an investor, the decision of re-balancing a portfolio, and from the point of view of a regulator, interventions in the market in order to lessen the contagion of a shock in a specific sector.

We find those tools in the theory of random graphs. Specially in the form of local metrics (in the paper, degree- , eigen- and betweenness- centralities) to classify the interconnectedness of stocks and a global metric (the modularity computed from correlation matrices) to detect *communities* of stocks.

The described approach integrates into the research agenda from the point of view of [52] and is a very active research area; see e.g., the survey [12]. However for the Mexican stock exchange there are few works in this direction. In the next section we present related literature. Note however that we do not pretend to give an exhaustive list on this active topic which deserves a survey by its own, but to give a brief panorama on activity for this research area.

## 2.1 Stock markets from GGm, random graphs, and network-theory approaches

Gaussian graphical models, Random graphs, and Network theory approaches in a financial context is an active research area attracting more and more attention with an increasing number of papers; see e.g., the survey [12]. The following is a non exhaustive list just describing different approaches and applications.

Papers in finance reporting an approach related to a graphical model i[illegible]de [17], [1], [18]. However, none of these papers focus on asset prices. Theoretical background on graphical models can be found on [61], [29], [3].

Papers studying financial networks based on partial correlations include [36], [62], [4] and [25]. Papers with applications based on a network approach in a financial context include (a) spillover effects and shocks contagion, [53], [37], [26], [4], [11] (b) portfolio selection, [9], [43], [46] (c) detection of stock prices manipulation [55] (d) portfolio diversification [6]. Papers studying financial networks based on the distributions' tails of prices/log-returns include [9], [64], [51], [21], [5], [63].

Some studies determine power laws for degree connectedness defined by assets correlation matrices; see [36], [58], [24], [6], [41], [7]. Papers studying community detection in a financial context include [36], [33], [2], [22], [44]. See [15] for a survey on methods for community detection.

Minimal Spanning Trees applied to financial market ranking include [20], [30], [62], [7], [34]. Random matrix theory for correlation matrices has been presented in [4], [33], [50], [45].

## 2.2 Subprime crisis

According to [56] there indeed existed an impact from the 2007-2008 subprime crisis in the Mexican economy. Mainly due to two shocks, first, a decline in Mexico's exports and second, a constrained access to international financial markets. Thus, evidencing an integration of the Mexican stock exchange with the US market. A phenomenon documented by some authors; see e.g., [48, 49, 60]. Figure 1 illustrates price levels for the main index IPC in the Mexican stock market for the years 2006, 2007 and 2008. It can be argued the presence of a bullish market on 2006 while on the second semester of 2008 the market turned bearish. Not surprising and reported by some authors [42, 23]. Later we will go beyond a visual examination and confirm by a multivariate GARCH model through a shift from positive to negative intercepts on log returns of each time series of the period; see Section 6.2 below. However, quite interesting, we will show that the partial-correlations interdependency structure of the Mexican financial market does not show a dramatic change as response to that shock for price levels, see Figure 3 and Section 3.4.

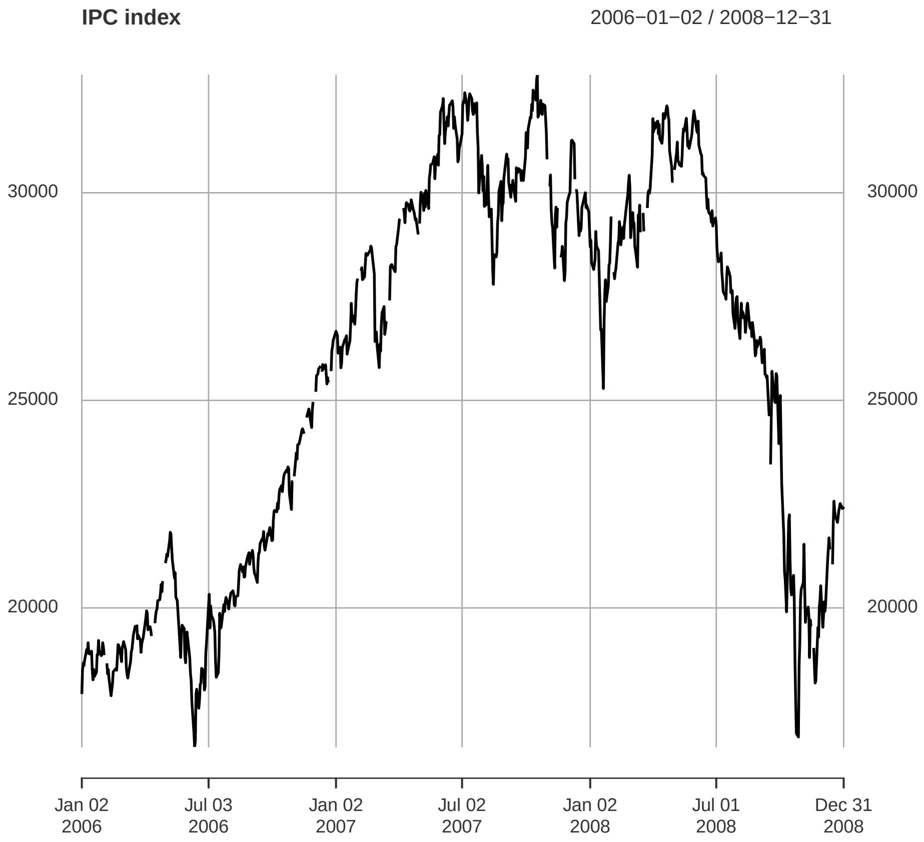


Figure 1: IPC index. Time series for the period 2006-2008.

## 2.3 Data

We constructed a database of daily closing prices from publicly available information at Yahoo.Finance website. The complete list of analyzed stocks can be found in the electronic supplementary material. The frequency of data is daily in a span of time comprising 01-01-2000 to 31-12-2019. We considered all trough the paper, time series of log returns: $R_t(i) = \log\left(\frac{S_{t+1}(i)}{S_t(i)}\right)$ where $S(i)$ is the price level of stock labeled $i$.

Data is organized in windows of one year (from january to december) and applied a filtering process in two steps. In the first step, for each year, stocks in the market with the most complete information were selected. The criterion was that only stocks with more than 90% of all the available dates were selected. Then, in a second step, stocks prices not having a minimum of variance in moving windows spanning 30 dates were discarded. This filtering process already presents the interesting fact of a positive evolution of the Mexican market for equities in the sense of an increase of activity. Indeed,

as we go forward along the years, more and more time series of stocks prices satisfy the filtering process evidencing an evolution in terms of more activity in the market with more variability of prices and more quotes. Visual evidence can be found in Figure 3. An important aspect of this work will be to consider how industrial sectors are interconnected. Here we consider a list of sectors obtained from a BMV's classification. These are listed upnext in Table 1. Figure 2 presents already an estimated network in which stocks can be identified in its sector.

Table 1: Industrial sectors

| | Sector | No. Stocks | Description |
|---|---|---|---|
| 1 | Basic consumming | 21 | Manufacturers and distributors, food and beverage companies |
| 2 | Energy | 2 | Energy producers, equipment, services and distribution |
| 3 | Financial services | 25 | Includes banks, financial and insurance firms |
| 4 | Health | 4 | Care providers, equipment, supplies and pharmaceuticals |
| 5 | Industry | 36 | Include companies providing equipment and services in the productive chain |
| 6 | IPC index | 1 | Main index in the Mexican stock exchange |
| 7 | IT | 1 | Software and Hardware for information technologies |
| 8 | Materials | 22 | Include companies providing input materials in the productive chain |
| 9 | Non basic consumming | 19 | Includes retailers, consumer service providers and consumer durables |
| 10 | Telecomm | 9 | Includes wireless providers, internet service providers and satellite companies |

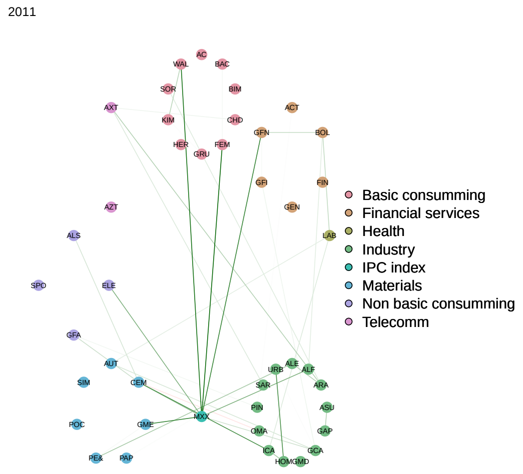


Figure 2: A network in which stocks can be identified in its sector.

# 3 Gaussian Graph model

## 3.1 Markovian Random Fields

In this section we start with the basic definition of a MRF which is the fundamental probabilistic concept from which a GGm is defined. Let us introduce a graph $G =$

$(V, E)$ with a set of nodes $V = \{1, \ldots, n\}$ and edges $E$. Recall that a complete subgraph of $G$ is called a *clique*. We denote by $C$ the class of *maximal cliques* of the graph $G$. Let be given a random vector $\vec{X} = (X_1, \ldots, X_n)$ with multivariate accumulative distribution function $p$. Then, the vector $\vec{X}$ has a *Gibbs distribution* compatible with the graph $G$ if its distribution has a representation

$$p(x_1, \ldots, x_n) = \frac{1}{Z} \prod_{C \in C} \psi_C(x_C),$$

where $\{\psi_c\}_{C \in C}$ are suitable functions and $x_C$ denotes a vector in which only the indexes of $C$ appear. Gibbs distribution are characterized through different Markov properties. To this end, we need a notation. For $A \subset V$, $A = (A_{i_1}, \ldots, A_{i_k})$, the notation $X_A$ denotes the vector $(X_{i_1}, \ldots, X_{i_k})$. The next list provides Markov properties:

1. $\vec{X}$ is a MRF with respect to $G$ if it has the Markov property: For any pair $i, j \in V$ with $i \neq j$ and non adjacent in the graph $G$, the random variables $X_i$ and $X_j$ are conditionally independent on all the other variables. We denote this conditional independency by:
$$X_u \perp\!\!\!\perp X_v \mid X_{V/\{u,v\}}.$$

2. $\vec{X}$ is locally a MRF with respect to $G$ if: For each $v \in V$, the random variable $X_v$ is conditionally independent of all other variables which are not neighboors (they are not adjacent). We denote this by
$$X_v \perp\!\!\!\perp X_{V/neighborhood(v)} \mid X_{neighborhood(v)}.$$

3. $\vec{X}$ is globally a MRF if: For two disjoint subsets $A, B \subset V$, the vectors $\vec{X}_A$, $\vec{X}_B$ are conditionally independent on a separating set $S \subset V$. We denote this by:
$$X_A \perp\!\!\!\perp X_B \mid X_S.$$

The next is a fundamental equivalence result; see [19, Chapter 7].

**Theorem 1** (Hammersley-Clifford)**.** *Assume that the distribution $p$ of $\vec{X}$ is defined in a finite state space and is positive valued. Then $p$ is a Gibbs distribution if and only $\vec{X}$ satisfies any of the Markov properties.*

For a list of Gibbs distributions see e.g., [61, Section 3]. In this paper we will work with the following specific Gibbs distribution (hence, specific MRF and specific GGm)

$$p_\theta(x) = \exp\left\{\theta \cdot x + \frac{1}{2} \sum_{i=1}^{m} \sum_{i=1}^{m} \Theta_{i,j} x_i x_j - A(\theta)\right\}. \tag{1}$$

where $A(\cdot)$ is a normalizing constant; see [61, Example 3.3] for more details. The MRF model in (1) specifies also the GGm we will work with. Indeed, (1) does not apriori specify any graph, but from the set of parameters $\Theta_{i,j} \in \mathbb{R}$ we derive a partial correlation matrix which indeed can be seen as the adjacency matrix of a weighted graph.

## 3.2 Covariance selection

Let $\Sigma$ be the covariance matrix of a random vector $(R_1, \dots, R_n)$ with multivariate Gaussian distribution. A zero component $\Sigma_{i,j} = 0$ expresses marginal independence between $R_i$ and $R_j$. On the other hand, the inverse matrix $J := \Sigma^{-1}$, the so-called concentration matrix, has the property that a zero component $J_{i,j} = 0$ expresses conditional independence; see e.g., [32, Thm. 9.2.1] or for complex distributions [3, Thm 7.1 p. 117]. This property of multivariate normal distribution is fundamental in covariance selection; see [10].

Latest developments on covariance selection focus on sparse large dimensions in which the number of variables is large but also there are many variables which are conditionally independent; see [35]. Thus, for such structures the concentration matrix is sparse and the lasso (least absolute shrinkage and selection operator; also lasso or LASSO) method introduced by [57] is fundamental for statistical estimation and variable selection. Indeed, the Gaussian Graphical model that we are going to use is "nodewise estimated" through a lasso procedure. For the lasso implementation we use R package mgm that builds on the package glmnet. The estimations of this last package are based on the algorithm of [16]. Then, the collection of nodewise regressions are combined through an AND rule to give a unique estimation of a multivariate vector. This approach is naturally based on the asymptotic consistency results due to [35]. In particular, the estimation yields a concentration matrix $J$. Systematic presentations for graphical models can be found in [61], [29], [3].

## 3.3 The GGm

Now we explain the specification of the GGm we are going to estimate. Let $\Sigma$ be the covariance matrix of the log returns time series $R(1), \dots, R(n)$. Denote by $J$ the concentration matrix, $J := \Sigma^{-1}$. Indeed, the components of the matrix $J$ are given in terms of the coefficients $\Theta_{i,j}$ in equation (1). Denote by $\rho_{i,j}$ the partial correlation of $R(i)$ and $R(j)$. Consider the linear regressions defining partial correlations:

$$R(i) - \mu(i) = \sum_{j \neq i} \beta_{i,j}(R(j) - \mu_j) + \epsilon(i) \tag{2}$$

where $\mu_i$ is the unconditional mean of $R(i)$ and $\epsilon(i)$ is a residual. Then

$$\beta_{ij} = \frac{\rho_{i,j}}{\sqrt{var(\epsilon(i))var(\epsilon(j))}}. \tag{3}$$

It is also true that

$$\rho_{i,j} = \frac{-J_{i,j}}{\sqrt{J_{ii}J_{jj}}}.$$

The adjacency matrix $\mathbf{P} = (P_{i,j})$ is defined by

$$P_{i,i} = 0 \text{ and } P_{i,j} = \rho_{i,j}. \tag{4}$$

**Remark 1.** *Let us emphasize now that the estimation of the GGm* (1) *will ultimately result in the matrix* $\mathbf{P}$ *and this matrix is our main input for this section.*

### 3.4 Results from GGm estimation: Stylized facts

In this section we report the results on estimating a GGm for each year in the period 2000-2019 with specification (1). From this estimation exercise, we get a list of partial correlation matrices **P** for the years in the period 2000-2019. These matrices are available as supplementary material in csv files with the label 30LogreturnsMGMpartialcorr. A graphical representation of partial correlations is displayed in the panel of Figure 3 and a complete list of most strong partial correlations in the interval $[0.3, 1]$ and $[0.2, 0.3]$ can be found in Appendix A. The complete list is available as supplementary material. From them, we have the following stylized facts:

- First of all we see in Figure 3 a stable continuous evolution of partial-correlations interdependence structure. At this stage of a visual inspection, if there existed an impact of global crisis episodes (e.g., dot.com bubble, the subprime crisis and the European debt crisis) it doesn't seem to produce large variations in network interdependency structures.

- There are several edges with a weight (partial-correlation) above the threshold 0.2 which frequently include the main index from the Mexican stock Exchange BMV denominated IPC (quoted as MXX in Yahoo.Finance); see the tables in Appendix A.

- As we move forward in time, the market grows (with more nodes of stocks consistently quoted by year). However, it does not seem to be evidence that interconnectedness in the market changes drastically from one year to the other, even for the subprime crisis period.

- Connections must be due to exogenous factors to the market, but inherent to each stock, since the graph is based on partial correlations. However, for edges that involve as a node the IPC, it can happen that the other node is a stock involved in the construction of the index.

- A large number of links between stocks in different sectors. An empirical fact reported for other markets; see e.g., [36]. To our best knowledge, not previously documented for the Mexican stock market. Nonetheless, intrasectorial partial-correlations are also present.

- There are persistent links between pair of stocks that along the twenty years period appear frequently but not systematically; see Apendix B.

- Negative partial-correlations appear only seldom.

- For the year 2000 we see a partial correlation of 0.98 between ICA and ELEKTRA which apriori looks as an odd finding. But this is actually supported by data; see Figure 5.

- The strongest links above 0.3 are those typically having as one of its nodes the IPC. Also for the rank $[0.2, 0.3]$ links with IPC as a node dominate but with a little decline in frequency in contrast with the interval $[0.3, 1]$.

- FEMSA indeed has a persistent relationship with IPC with partial correlations above 0.3; see the table in Appendix B. In this same table we do not see an important stock as AMX. This is an interesting confirmation for GGm model' strength, since it captures a realistic fact; see e.g., the news stories expansion and el economista, etc.

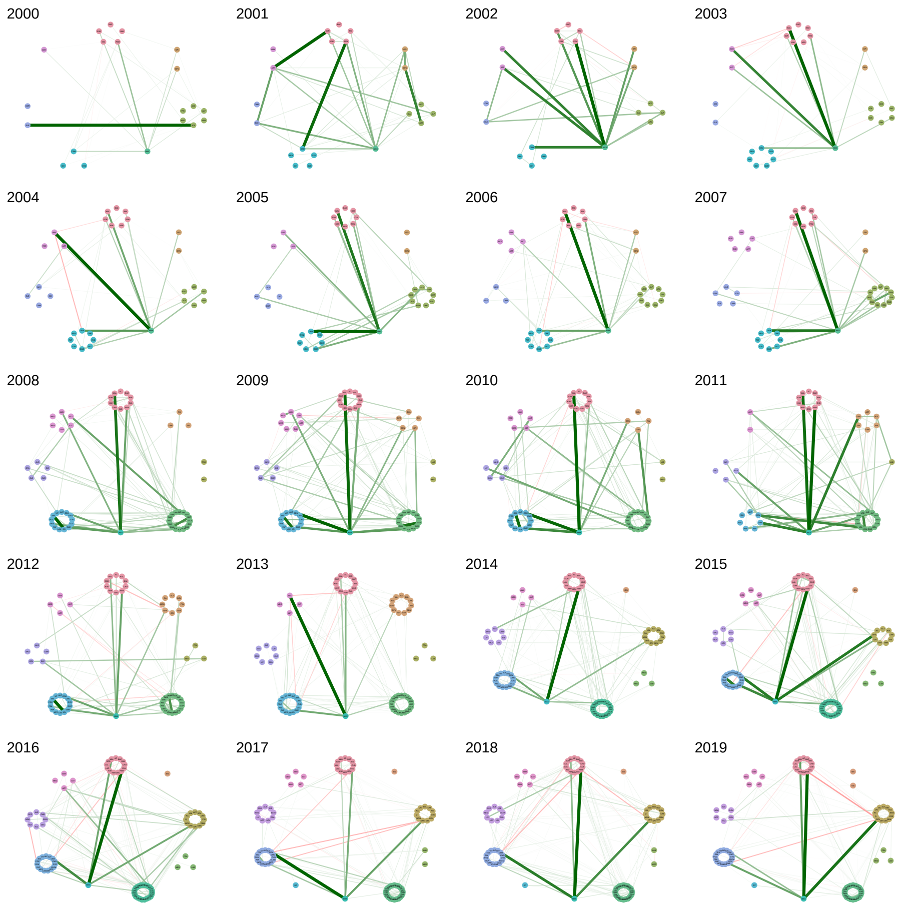


Figure 3: Graphs associated to partial correlation matrices by year in the period 2000-2019. For a given edge the green color (resp. red color ) represents a positive (resp. negative) relationship. Edge's width represents strength of correlation. A list of partial correlations in different ranks can be found in Appendix A. Vertexes are grouped according to its industrial sector.

In the tables from Appendix B we see the most "persistent" relationships between stocks for which partial correlations in absolute value were in a given interval for nine or more years. Quite notoriously they are rare and almost always involves the index

IPC. In Figure 4 we see a panel of barplots for degree-centralities separated into different ranges for all stocks in their respective period. As we already mentioned for the red lines in Figure 6, links with negative values are few in quantity and magnitude as more precisely illustrated in Figure 4a. In Figure 4b we see a quite homogenous distribution in the range $[0.01, 0.1]$. An analogous situation is appreciated in Figure 4c in the interval $[0.1, 0.5]$. Only in the range $[0.5, 1]$ we see in Figure 4d a more heterogenous situation with some dominating stocks.

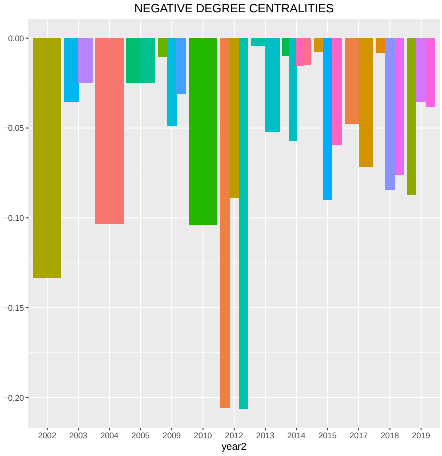


(a) Only negative values are plotted.

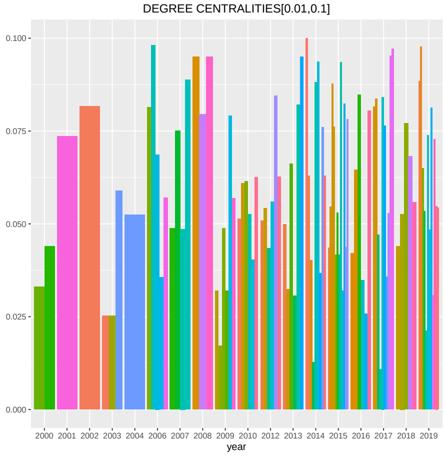


(b) Values in the range $[0.01, 0.1]$ are plotted.

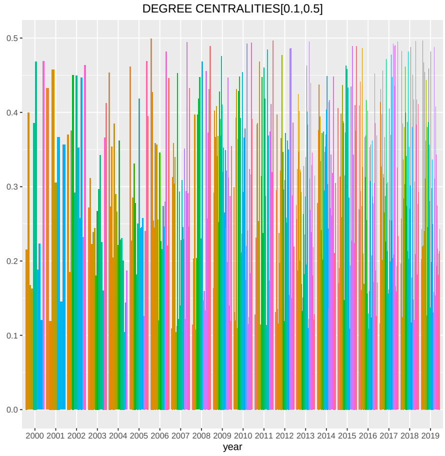


(c) Values in the range $[0.1, 0.5]$ are plotted.

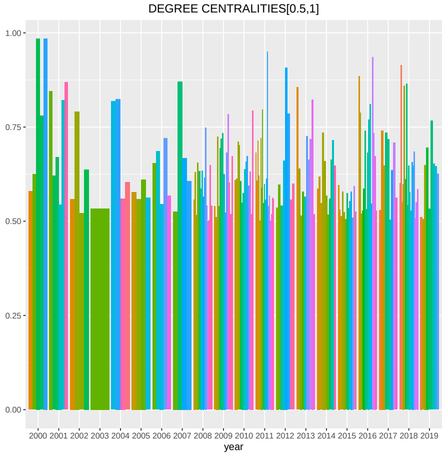


(d) Values in the range $[0.5, 1]$ are plotted.

Figure 4: A comparison of degree centralities by year at different ranges.

Before we continue with a discussion of results in this section, we estimate metrics (centralities) from network theory to see a possible effect of the global financial crisis; see Section 4.3 below.

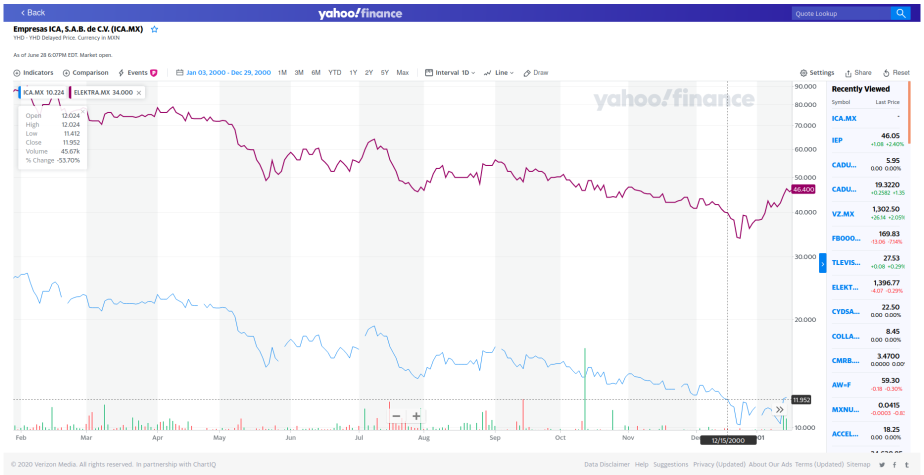


Figure 5: In purple the time series for ELEKTRA and in blue the time series for ICA. Prices in logarithmic scale for the year 2000. Source: Yahoo.Finance.

# 4 Centralities from partial correlations

Centrality is a measure conceptually designed in such a way that a vertex with high centrality can be considered highly influential. The first concept of centrality we use is the *degree-centrality* which for a vertex in a weighted network is just the sum of all connecting edge's weights. For our graphs of partial correlations, the degree centrality gives information of the pattern of a shock's transmission. The idea is that an influential (i.e., with high centrality) stock in the financial network having a bad day, is accompanied with many other stocks in the same situation. *Note that there is no causality claimed here*. The second measure of centrality that we estimate is the *eigen-centrality*. This is a global measure in that scores for each node are assigned by a contrast of the quality of its links. For example a node with just one link to another influential node could have a highest eigen-centrality than a node with two or more links. The computation of eigencentralities transfers to a spectral analysis of the adjacency matrix and in crucial steps is substantiated by Perron-Frobenius theory (see e.g., [54, Chapter 17]). The third concept that we estimate is *betweenness-centrality*. For each vertex, it gives the proportion of shortest paths passing through it.

## 4.1 Shock transmissions

Let us explain with more detail about eigencentrality and at the same time also clarify about shocks transmission. Let $V = \{1, \ldots, n\}$ denote our set of stocks and recall the matrix $\mathbf{P}$ defined in (4). The eigencentrality is a function $f : V \to \mathbb{R}$ satisfying

$$f(v) = r \sum_{w \in N(v)} \mathbf{P}_{v,w} f(w), v \in V, \tag{5}$$

where $r$ is a non negative constant and $N(v)$ denotes the neighbors of $v$. Note that

$$f(v) = r \sum_{w \in V} \mathbf{P}_{v,w} f(w),$$

since by definition $w \in N(v)$ if and only if $\mathbf{P}_{v,w} \neq 0$. Now this can be written in matricial notation as

$$f(V) = r\mathbf{P}f(V)^T,$$

where $f(V) = (f(1), \ldots, f(n))$. Hence, $f(V)$ is an eigenvector of $\mathbf{P}$ attached to $r$ as its eigenvalue.

To continue we follow the discussion in [4], recall the coefficients $\beta_{i,j}$ in equation (3). The matrix of coefficients $\mathbf{B} = (\beta_{i,j})$ with $\beta_{ii} = 0$ is then connected to the adjacency matrix as $\mathbf{B} = diag(J)^{-\frac{1}{2}}\mathbf{P}diag(J)^{\frac{1}{2}}$. We can write the linear regression in a compact matricial notation as

$$R - \mu = \mathbf{B}(R - \mu) + \epsilon = diag(J)^{-\frac{1}{2}}\mathbf{P}diag(J)^{\frac{1}{2}}(R - \mu) + \epsilon. \tag{6}$$

Let $\tilde{R}(i) := R(i) - \mu(i)$ and $\tilde{R} = (R(1) - \mu(1), \ldots, R(n) - \mu(n))$. Then,

$$diag(J)^{\frac{1}{2}}\tilde{R} = \mathbf{P}diag(J)^{\frac{1}{2}}(R - \mu) + diag(J)^{\frac{1}{2}}\epsilon.$$

Hence the vector $X := diag(J)^{\frac{1}{2}}\tilde{R}$ satisfies

$$X = \mathbf{P}X + \eta$$

where $\eta := diag(J)^{\frac{1}{2}}\epsilon$.

Now assume that between times $t_0$ and $t_1$ there is a shock $\Delta = (0, \ldots, 0, \delta, 0, \ldots, 0)$ affecting $X(i)$. Then, $X$ at time $t_1$ is given by $\mathbf{P}(X + \Delta) + \eta$ and the change is then $\mathbf{P}\Delta$. Note that $\mathbf{P}\Delta$ does not need to be a scalar of $\Delta$, meaning that the shock affecting originally to $X(i)$ is also affecting to other components indicating that the shock propagates.

The spectral decomposition of $\mathbf{P}$ helps on assessing the reach of propagation and rationalizes the definition of eigencentrality. Let $W_1, \ldots, W_n$ be the set of eigenvectors of $\mathbf{P}$ and $\Lambda = \{\lambda_1, \ldots, \lambda_n\}$ the corresponding set of eigenvalues, which we assume is decreasingly ordered with respect to its modulus. Here is a common assumption: there is a unique eigenvalue attaining the spectral radius. This means $|\lambda_1| > |\lambda_2| \geq |\lambda_2| \ldots \geq |\lambda_n|$. If the matrix $\mathbf{P}$ has only nonnegative components, Perron-Frobenious theory guarantees we are in this situation and even more properties; see e.g., [54, Chapter 17]. Represent $\Delta$ by $\Delta = \sum_i \alpha_i W_i$. Then, for $k \in \mathbf{N}$

$$\mathbf{P}^k\Delta = \lambda_1^k \left\{ \alpha_1 W_1 + \sum_{i=2}^{n} \left( \frac{\lambda_i}{\lambda_1} \right)^k W_i \right\}.$$

Hence

$$\lim_{k \to \infty} \frac{1}{\lambda_1^k} \mathbf{P}^k \Delta = \alpha_1 W_1.$$

Then, as time runs the leading term indicating the effect of the initial shock $\Delta$ takes the form $\lambda_1^k \alpha_1 W_1$.

## 4.2 Results of estimation

In Figure 6 we see estimated centralities for our networks. The blue line is the largest modulus per year of eigenvalues. The green line represents the maximum degree-centrality for each year. Very unsurprising this maximum is always attained by the index IPC. The red (resp. red and dashed) line represents the average of each node's degree-centrality (resp. average of each node's absolute value degree-centrality). The gray line represents the maximum betweenness-centrality which has been computed with the R package igraph.

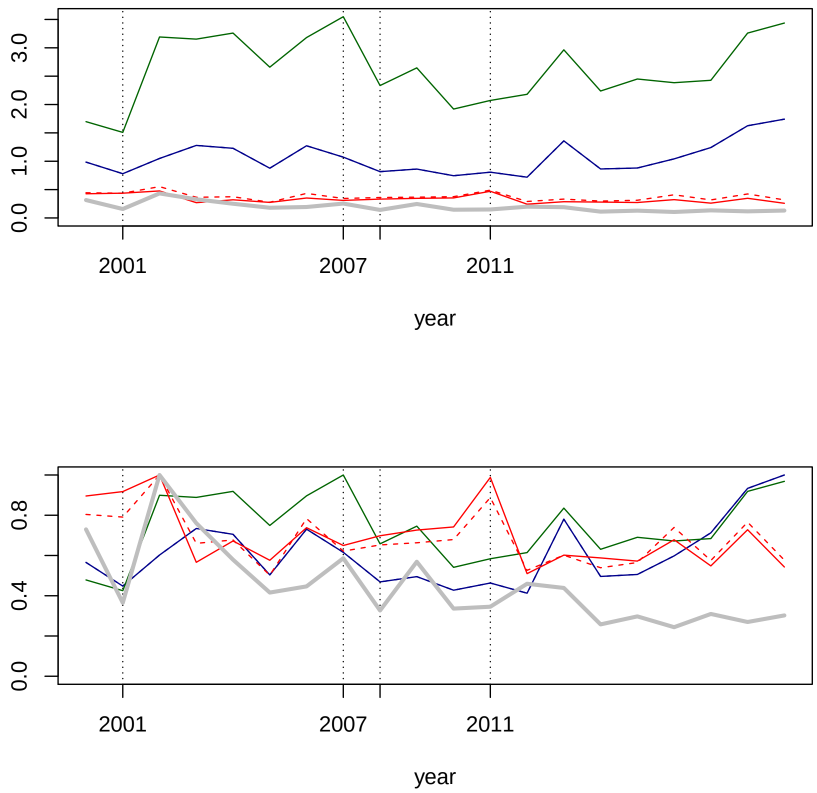


Figure 6: Network centralities from partial correlations. The blue line is the largest eigenvalue. The green line is the maximum degree-centrality by year, always attained by the index IPC. The red lines are averages of degree-centralities, resp. absolute value of degree-centralities. The gray line represents the maximum betweenness-centrality. In the upper panel, time series are shown in their original scale, while in the lower panel time series have been rescaled by its maximum.

These are the facts we observe from Figure 6:

- the spectral radius is approximately bounded by two, which coincides with the range documented for other markets; see e.g., [36].
- The red line and the red dashed lined are almost indistinguishable. This happens as a consequence to the fact that almost all partial-correlations are non negative. We also observe the stability on the metric represented by this line.
- The patterns of the green line and blue line are similar. As we mentioned, the green line is attained by the IPC. So it could be expected that also the blue line is related to this index. Although we do not go into this claim, assuming it is correct, in order to capture effects beyond the IPC it might be necessary in this case to complement with the second eigenvalue together with its eigenvector for centrality and the analysis for a shock contagion. Indeed, Figure 7 shows that in many cases the dominant eigenvalue has multiplicity two or more, and in other cases that the second eigenvalue turns out to be close to the first. Certainly, the idea of considering beyond the dominant eigenvector for eigencentrality is not new; see e.g., [40]. Analysis for the Mexican case will be addressed elsewhere.
- There is indeed variability for centralities, but changes from one year to the other are indeed relatively small. Thus, changes are subtle. For example, for the subprime crisis period, we see a small upwards jump from 2005 to 2006 of around 0.4 and then from 2007 to 2008 a downwards jump of around 0.25. Small jumps are also observed for max degree-centrality in the green line.
- Continuing with the previous point. We see an abrupt upwards movement for the green line which is reasonable to associate with the dot.com bubble's crisis: From the year 2001 to 2002.

### 4.3 Discussion

The financial networks of partial correlations illustrated in Figure 3 present low degree centrality and are sparse. In Section 6 we construct financial networks based on Pearson correlation matrices, and we will also compute their centralities; see Figure 12. A comparison of Figures 6 and 12 yield evidence that partial-correlations generate sparser networks, understood as the fact that comparing year by year, centralities for partial correlations are significantly lower than for Pearson correlations. This was expected and agrees with the findings in [36] and [4] who also compare networks based on Pearson and partial-correlations. Interestingly, estimations of matrices are done by different methods and different data. Thus, providing evidence that sparsity of partial-correlation based networks are robust with respect to statistical procedures and data. Another paper that also compare networks based on Pearson and partial correlations is [62]. They conclude that networks based on Pearson correlations present different structure in regard to partial correlation matrices, in particular presenting different clustering structure. However, they construct Minimum Spanning Trees and it is not clear how to compare if one network is more sparse relative to the other. They also compute

betweenness-centrality. Although they report differences, these are not as marked as the ones presented here. We will elaborate on betweenness-centrality further in Section 5.1.

The index IPC has been found to be a vertex where edges consistently present their highest weight (partial-correlation). Indeed, the maximum of degree- and eigen-centralities always attained at this vertex. If the analysis of the mean variance portfolio of [43] holds true also for partial correlations, then, this would say that in such portfolio, the IPC seen as an asset by its own would receive a lower weight. Thus, although the IPC is indeed informative, as any index should be, any tracking ETF does not diversify investments from the point of view of the classical Markowitz portfolio theory and receive a lower proportion of portfolio value. Whether the negative relationship found in [43] also holds true for partial correlations can be the subject of future research, we find that the index IPC has also high degree- and eigen- centralities for networks based on Tail-dependence and Pearson correlation matrices; see Sections 5 and 6 respectively.

In the period 2000:2019 there are three important financial episodes: The dot.com bubble, the subprime crisis and the European sovereign debt crisis. The time series of centralities in Figure 6 exhibit several local maximum that one may attach with those episodes. Now here is a trade-off. Partial correlations and the lasso estimation of the GGm indeed result in a stringent sieve in which only most significant and "clear" relationships pass through. Out of this reason the centrality time series are quite stable. However, the afore mentioned financial episodes are captured by centralities of partial-correlations and show moderate increases. We will see in Section 5 that Tail-dependence networks exhibit a more sensible topology to those market conditions. This is reasonable due to the symmetric nature of distributions in GGm while on Tail-dependence networks emphasis is on lower tails.

# 5 Tail-dependence networks

For two random variables $X$ and $Y$ the Tail-dependence coefficient ([31]) is the limit

$$\lambda_L = \lim_{q \to 0} \mathbb{P}\left(X \leq F_X^{-1}(q) \mid Y \leq F_Y^{-1}(q)\right)$$

where $F_X$ represents the distribution of $X$, and similarly for $F_Y$. It is clear that $\lambda_L$ quantifies the relationship of lower tails between $X$ and $Y$. In this section we focus on networks based on matrices whose every component is the coefficient $\lambda_L$ for pairs of stock time series. We estimate the Tail-dependence coefficient through the non-parametric estimator in [47] which is implemented in the R package FRAPO (Financial Risk Modelling and Portfolio Optimisation).

In Figures 8 and 9 the Tail-dependence networks for years in the period 2006:2009 are illustrated. As before, the complete list is provided in the electronic supplementary material. In Figure 8 vertex size is a function of betweenness-centrality while on Figure 9 it is a function of eigen-centrality. The illustrated structure repeats for the other years in the period 2000:2019. The time series of centralities are illustrated in Figure 10. This is a list of stylized facts:

1. The index IPC is also important with respect to eigen-centrality, just as it was for partial-correlation networks.
2. For betweenness-centrality the 95% quantile is quite dynamic, concentrated in a few stocks, and consistently, does not include the index IPC.
3. The 95% quantile is more distributed for eigen-centrality than for betweenness-centrality.
4. Figure 10 clearly shows that time series of centralities experience an increase in activity associated to the main crisis of the subprime crisis and the European sovereign debt crisis.

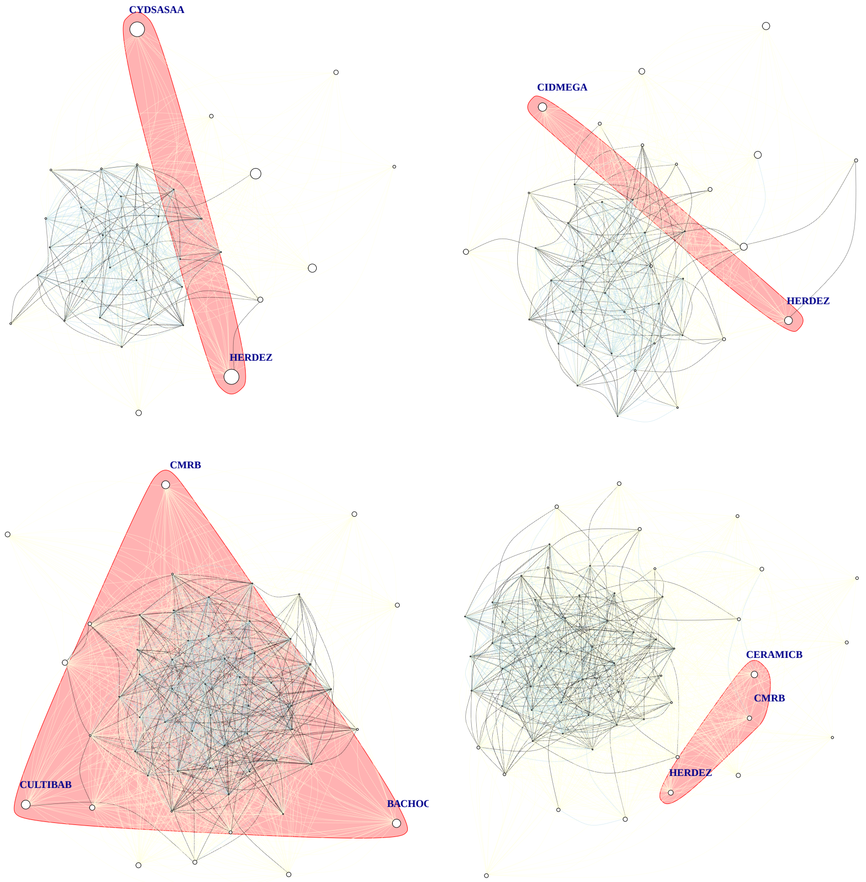


Figure 8: Tail-dependence networks for the years 2006-2009. Vertex size as function of **betweenness-centrality**. Pink shaded area shows highest 95% quantile. Edges colors are black for weights in the interval $[0.2, 0.3]$, and light blue in the interval $(.3, 1]$. Otherwise the color is light yellow.

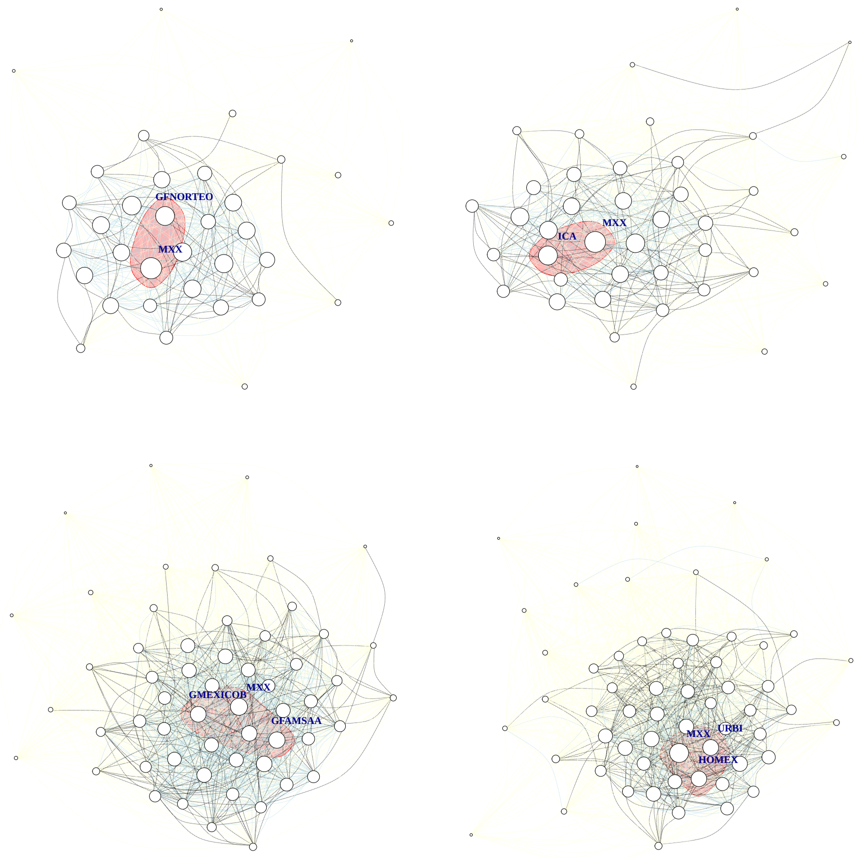


Figure 9: Tail-dependence networks for the years 2006-2009. Vertex size as function of **eigen-centrality**. Pink shaded area shows highest 95% quantile. Edges colors are black for weights in the interval $[0.2, 0.3]$, and light blue in the interval $(.3, 1]$. Otherwise the color is light yellow.

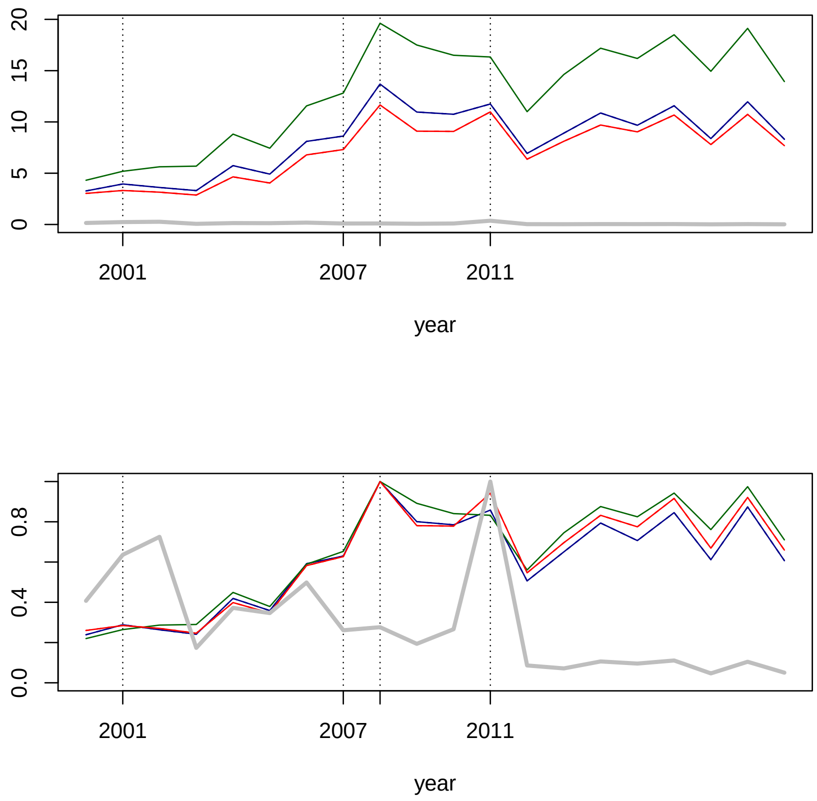


Figure 10: Network centralities from Tail-dependence networks. The blue line is the largest eigenvalue. The green line is the maximum degree-centrality by year. The red line is an average of degree-centrality. The gray line represents the maximum betweenness-centrality. In the upper panel, time series are shown in their original scale, while in the lower panel time series have been rescaled by its maximum.

## 5.1 Discussion

In Figure 10 we see an illustration of financial network's interdependency-evolution for the Mexican stock exchange. The first fact to note are the peaks in the years 2008 and 2011. It is reasonable to associate such increments in interconnectedness (as measured by degree- and eigen-centrality) to the subprime financial crisis and the European sovereign debt crisis. This is coherent with the findings in the literature. It is worth emphasizing that on this literature, other data have been analyzed with quite different methods. For comparison, let us recall a few papers in this regard. A financial network from 100 selected stocks of financial institutions in the US is studied in [21]. Emphasis

is on tail events from the point of view of systemic risk. They find that the banking sector is at the core of systemic risk between 2008 and 2010. In contrast, the insurance companies are less relevant for systemic risk. Their empirical results exhibit growing interconnectedness during the period of a financial crisis. A financial network based on Tail-distributions is studied by [5]. Data consists in a collection of 51 large European banks and 17 sovereigns bonds during the period from 2006 through 2013. Their empirical results show that network densities vary with the intensity of the (subprime) financial crisis. More precisely, network densities increases from 2006 up to its (local) maximum around the peak of the global financial crisis and then decreases. A different approach based on entropy is studied by [51]. The empirical result reports that "node strengths peak in times of crisis".

Hence, there exists an empirical fact manifest along a variety of methods applied to different data: estimated network-interconnectedness increase at some point in the development of a crisis, it might indeed affect one sector more than other, yet it is going to be globally observable. We evidence this empirical fact for the Mexican stock exchange in Figure 10. This is already interesting, and going deeper into the details, we mention two subtleties about timing and the different centralities. We have observed in partial-correlation networks that degree- and eigen-centrality show the same pattern. This similarity in patterns happens also for the Tail-dependence networks analyzed in this section; compare Figures 6 and 10. It will be observed again for networks based on (filtered) Pearson correlations 12. However, betweenness-centrality exhibits a different pattern, more so for Tail-dependence networks. A relevant difference to be emphasized is about the timing of local extrema. If this difference is indeed robust with respect to data and statistical procedures certainly is an interesting question for future research. Difference in patterns, appreciated in the time series, is also visualized from the networks in Figures 8 and 9. In the former, in which node size is a function of betweenness-centrality, the most influential nodes are linked exclusively through yellow edges. These nodes have a lot of links although all of them have small weights. On the later graph, where node size is a function of eigen-centrality, the most influential nodes are connected among them through blue links. This means weights in the interval (.3, 1].

# 6 Network theory: Community detection

## 6.1 DCC Multivariate Garch model

Let $y := \{y_t\}_{t=1}^N$ denote a one dimensional time series with $N$ observations. A GARCH specification for its volatility usually starts with a flux of information determined by a filtration $\{\mathcal{F}_t\}_{t=1}^N$ in which $\mathcal{F}_t$ is a $\sigma$-algebra representing information at time $t$ and $y$ follows the dynamic

$$y_t = E[y_t \mid \mathcal{F}_{t-1}] + \epsilon_t(\theta).$$

Here $\theta$ is a parameter vector whose specification specializes the model, $\mu(\theta)$ is the conditional mean of the time series at time $t$, usually modeled through an ARMA time series. For example an ARMA(1,1) (as we will consider here) is specified by

$$\mu_t = \mu + \varepsilon_t + \phi\mu_{t-1} + \psi\varepsilon_{t-1}, \tag{7}$$

where $\phi$, $\psi$ are parameters to be estimated and $\varepsilon$ is white noise, i.e. an uncorrelated centered time series. The residual $\epsilon(\theta)$ captures the conditional volatility of $y$:

$$var(y_t \mid \mathcal{F}_t) = E[(y_t - \mu_t(\theta))^2 \mid \mathcal{F}_t] = E[(\epsilon_t(\theta))^2 \mid \mathcal{F}_t] = var(\epsilon_t(\theta)).$$

Its specification is the essence of a GARCH model. We will consider the standard GARCH(1,1) model:

$$\epsilon_t = \sigma_t z_t \tag{8}$$

$$\sigma_t^2 = \alpha_0 + \alpha_1 \epsilon_{t-1}^2 + \beta_1 \sigma_{t-1}^2, \tag{9}$$

where $\{z_t\}_{t=1}^N$ is white noise.

Now consider a set of univariate time series $y(1), \ldots, y(n)$. A class of models in the *multivariate GARCH* literature known as Dynamic Conditional Correlation (DCC) was introduced by [14] and [59]. The DCC class builds up on univariate GARCH models and then specifies the dynamic of time varying conditional covariance matrix of the time series $y(1), \ldots, y(n)$. It has a general dynamics

$$H_t = D_t R_t D_t.$$

Here $D_t$ is a diagonal matrix of time varying standard deviations from univariate GARCH models and $R_t$ is a time varying correlation matrix. For estimation, the matrix $R_t$ is decomposed as

$$R_t = (Q_t^*)^{-1} Q_t (Q_t^*)^{-1}$$

where $Q$ is specified in [13, Equation (2)].

## 6.2 Means for the years 2006 and 2008

In Table 2 we report the coefficient $\mu$ in the specification (7) for each stock in the year 2006, analogously for Table 3 in the year 2008. The estimation of these coefficients provides further support to the claim reported in Section 2.2 after the visual evidence of Figure 1.

## 6.3 Modularity

Assume we are given an undirected and unweighted graph $G$ with vertexes $V = \{1, \ldots, n\}$ and edges $E$. Community structure in the graph means that there exists a partition of $V$ in groups of vertexes in such a way that within groups vertexes are highly connected and more edges exists among them, while at the same time, edges between groups are less observed; see [15] for a survey of methods in community detection. The afore description presents a general idea and to make it operative, it is necessary to give a more quantitative formulation. A popular approach is through the famous concept of modularity as introduced by [39] and further developed in [38]. Following the notation of [38] we introduce the following objects. Let $A$ be the adjacency matrix of $G$ and let $m = \frac{1}{2}\sum_i k_i$ where $k_i$ denotes the degree of vertex $i$ so $k_i = \sum_j A_{i,j}$. Further

Table 2: The coefficient $\mu$ for the year 2006.

| | Stock | mu value | Stock | mu value |
|---|---|---|---|---|
| 1 | ALFAA | 0.0006 | GISSAA | 0.0011 |
| 2 | ALSEA | 0.0026 | GMD | 0.0036 |
| 3 | AMXA | 0.0017 | GMEXICOB | 0.0021 |
| 4 | ARA | 0.0027 | HERDEZ | 0.0016 |
| 5 | AXTELCPO | 0.0013 | HOMEX | 0.0027 |
| 6 | AZTECACPO | 0.0006 | ICA | 0.0026 |
| 7 | BACHOCOB | 0.0012 | ICHB | 0.0036 |
| 8 | BIMBOA | 0.0018 | KIMBERA | 0.0011 |
| 9 | CEMEXCPO | 0.0013 | MXX | 0.0021 |
| 10 | CMOCTEZ | 0.0016 | PAPPEL | 0.0018 |
| 11 | CMRB | 0.0013 | PASAB | -0.0015 |
| 12 | CYDSASAA | 0.0016 | PE&OLES | 0.0027 |
| 13 | ELEKTRA | 0.0017 | PINFRA | 0.0065 |
| 14 | FEMSAUBD | 0.0024 | RCENTROA | 0.0039 |
| 15 | GCC | 0.0022 | SORIANAB | 0.0021 |
| 16 | GFINBURO | 0.0007 | URBI | 0.0018 |
| 17 | GFNORTEO | 0.0033 | WALMEX | 0.0023 |

denote by $\mathbf{s} \in \{1, \ldots, n\}^n$ a vector having the same dimension of $A$, and representing an allocation of vertexes to communities. Thus, $\mathbf{s}_i$ represents the community assigned to vertex $i$. Now the idea is to compare the graph $G$ with a graph $G'$ having no community structure. A group $V_k = \{i \in V \mid \mathbf{s}_i = k\}$ possess an accumulated weight of $\sum_{i,j \in V_k} A_{i,j}$. Now for $G'$, assuming it is a random instance of an Erdős-Rényi graph, the set $V_k$ should have an accumulated weight of $\sum_{i,j \in V_k} \frac{k_i k_j}{2m}$. Hence, the difference $\sum_{i,j \in V_k} A_{i,j} - \frac{k_i k_j}{2m}$ quantifies how distant is the immersion of community $V_k$ in the graph $G$ from $G'$. The modularity function is defined as the sum of these differences over all communities:

$$Q(\mathbf{s}) := \sum_k \sum_{i,j \in V_k} \left( A_{i,j} - \frac{k_i k_j}{2m} \right) = \sum_{i,j \in V} \left( A_{i,j} - \frac{k_i k_j}{2m} \right) \delta(\mathbf{s}_i, \mathbf{s}_j),$$

where $\delta(\mathbf{s}_i, \mathbf{s}_j) = 0$ unless $\mathbf{s}_i = \mathbf{s}_j$ in which case $\delta(\mathbf{s}_i, \mathbf{s}_j) = 1$.

As such, the modularity function $Q(\cdot)$ is defined for unweighted undirected graphs. In particular, for graphs obtained from a correlation matrix, which indeed is weighted, the modularity function $Q(\cdot)$ requires to be adjusted. Moreover, the null model (the graph $G'$) is critical for the well-functioning of modularity; see e.g., the discussion in [15]. Hence, to couple with this problem, we choose to work with the formulation of [33] where correlation matrix is filtered and modularity is adjusted for the right "null model" $G'$. The analysis is again based on a spectral analysis as we now explain. Let $C$ be a correlation matrix and consider the set of eigenvalues $\lambda_1, \ldots, \lambda_n$ which we assume are displayed in increasing order. Let $v_1, \ldots, v_n$ be the corresponding eigenvectors.

Table 3: The coefficient $\mu$ for 2008 year.

| | Stock | mu value | Stock | mu value | Stock | mu value |
|---|---|---|---|---|---|---|
| 1 | AC | -0.0013 | ELEKTRA | 0.0006 | IDEALB-1 | -0.0008 |
| 2 | ALFAA | -0.0019 | FEMSAUBD | 0.0019 | KIMBERA | 0.0002 |
| 3 | ALSEA | -0.0013 | FINDEP | -0.0036 | LAMOSA | -0.0016 |
| 4 | AMXA | -0.0023 | FRAGUAB | 0.0004 | MAXCOMA | -0.0028 |
| 5 | ARA | -0.0018 | GAPB | -0.0017 | MEDICAB | -0.0001 |
| 6 | ASURB | -0.0014 | GCARSOA1 | -0.0003 | MEGACPO | -0.0028 |
| 7 | AUTLANB | 0.0037 | GCC | -0.0032 | MXX | -0.0010 |
| 8 | AXTELCPO | -0.0053 | GFAMSAA | -0.0023 | OMAB | -0.0024 |
| 9 | AZTECACPO | 0.0000 | GFINBURO | 0.0008 | PAPPEL | -0.0047 |
| 10 | BACHOCOB | -0.0020 | GFNORTEO | -0.0003 | PASAB | -0.0030 |
| 11 | BIMBOA | 0.0002 | GIGANTE | -0.0026 | PE&OLES | -0.0010 |
| 12 | CABLECPO | 0.0000 | GISSAA | -0.0009 | PINFRA | -0.0016 |
| 13 | CEMEXCPO | -0.0030 | GMD | -0.0053 | POCHTECB | -0.0048 |
| 14 | CIEB | -0.0023 | GMEXICOB | -0.0032 | SAREB | -0.0033 |
| 15 | CMOCTEZ | -0.0005 | GRUMAB | -0.0001 | SIMECB | 0.0007 |
| 16 | CMRB | -0.0005 | HOMEX | 0.0007 | SORIANAB | 0.0010 |
| 17 | CULTIBAB | -0.0001 | ICA | -0.0006 | TMMA | -0.0040 |
| 18 | CYDSASAA | -0.0029 | ICHB | 0.0010 | URBI | -0.0018 |

Moreover, let $T$ be the number of observations and the critical values

$$\lambda_- := \left(1 - \sqrt{\frac{n}{T}}\right)^2, \qquad \lambda_+ := \left(1 + \sqrt{\frac{n}{T}}\right)^2.$$

The values $\lambda_-, \lambda_+$ are parameters for Marcenko-Pastur distribution in random matrix theory which is given by $\rho(\lambda) = \frac{T}{n}\frac{\sqrt{(\lambda_+-\lambda)(\lambda-\lambda_+)}}{2\pi\lambda}$. Define the matrices

$$C^r := \sum_{\lambda_i \leq \lambda_+} \lambda_i v_i^{\mathbf{tr}} \cdot v_i \tag{10}$$

$$C^g := \sum_{\lambda_+ < \lambda_i < \lambda_n} \lambda_i v_i^{\mathbf{tr}} \cdot v_i \tag{11}$$

$$C^m := \lambda_n v_n^{\mathbf{tr}} \cdot v_n. \tag{12}$$

We have a decomposition of the correlation matrix $C$ given by

$$C = C^m + C^g + C^r. \tag{13}$$

From the ordering of the eigenvalues, the matrix $C^r$ represents random noise, $C^m$ a global signal which in our financial context is attached to the market as a whole and $C^g$ represents information in a mesoscopic scale just between $C^r$ and $C^m$. Next, we explain how the modularity function $Q(\cdot)$ is adjusted. Accordingly, focusing in the matrix $C^g$, and taking into account the decomposition (13), the null model is $C^r + C^m$ and the

modularity functions takes the form

$$Q_3(\mathbf{s}) := \frac{1}{C_{norm}} \sum_{i,j} [C_{i,j} - C^r_{i,j} - C^m_{i,j}] \delta(\mathbf{s}_i, \mathbf{s}_j)$$
$$= \frac{1}{C_{norm}} \sum_{i,j} C^g_{i,j} \delta(\mathbf{s}_i, \mathbf{s}_j) \tag{14}$$

for $C_{norm} = \sum_{i,j} C_{i,j}$ a normalizing constant. However, the set of eigenvalues $\lambda_i$ satisfying $\lambda_+ < \lambda_i < \lambda_n$ could be empty (as we will find for some years in our sample). In this case the matrix $C^g$ will be undefined and makes no sense to consider it. For those cases we will consider a decomposition $C = C^s + C^r$ with $C^s := \sum_{\lambda_+ < \lambda_i} \lambda_i v_i^{\mathbf{tr}} \cdot v_i$ and then the modularity is defined by

$$Q_2(\mathbf{s}) := \frac{1}{C_{norm}} \sum_{i,j} [C_{i,j} - C^r_{i,j}] \delta(\mathbf{s}_i, \mathbf{s}_j)$$
$$= \frac{1}{C_{norm}} \sum_{i,j} C^s_{i,j} \delta(\mathbf{s}_i, \mathbf{s}_j). \tag{15}$$

Hence, in this section we maximize the modularity functions $Q_2$ and $Q_3$ in order to define communities and report on them. It is known that the maximization of modularity functions is a *NP-hard* problem; see [8]. Hence, the optimization is approached through several heuristic algorithms. We implement the popular Louvian algorithm, adjusted as described by [33] accordingly to the modularity functions $Q_2$ and $Q_3$.

## 6.4 Modularity function $Q_2$

In Figure 11 we see the resulting communities obtained with the Louvian algorithm applied to the modularity function $Q_2$ defined in (15). In all of the years of the period there are two communities. The first community is a “giant component” and the other community consist of a small number of isolated vertexes. Hence, at this scale our procedure does not detect a complex community structure. This is what we expected, since $Q_2$ is based on the matrix $C^s$ which includes the “market mode”. Note however the stylized fact:

- The turmoil at the subprime financial crisis and the European sovereign debt crisis periods are captured by a visually evident increase on interconnectedness. This can also be observed from the time series of centralities in Figure 12.

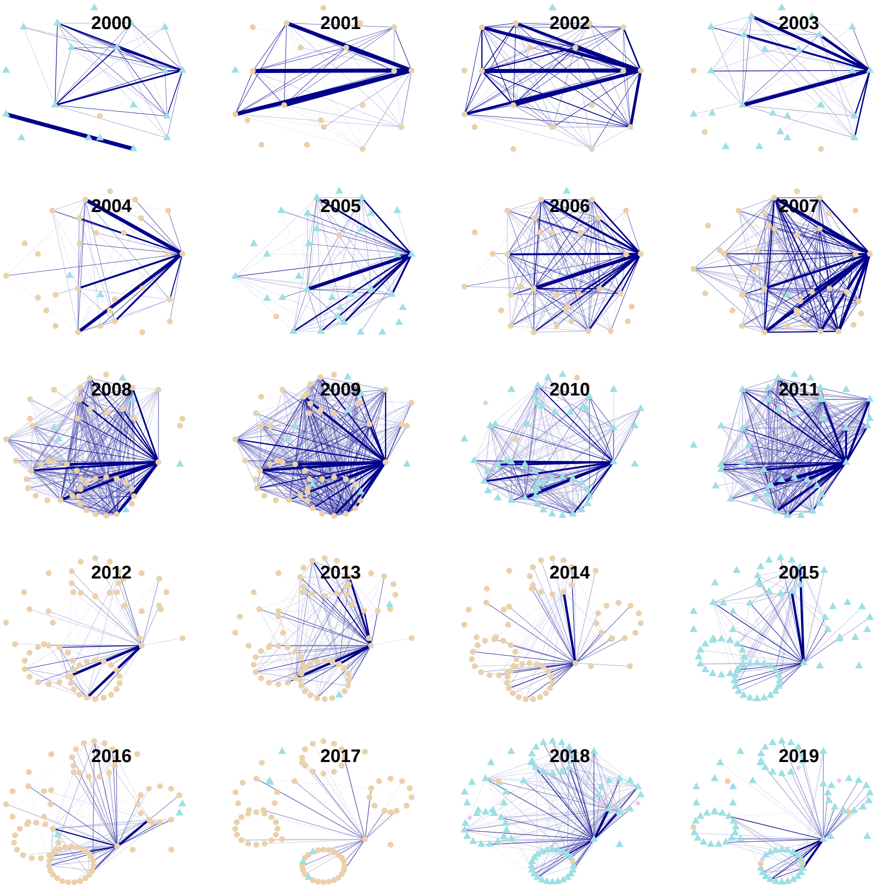


Figure 11: Communities obtained from modularity function $Q_2$. The color of the nodes represent community, which is equivalently represented by vertex' shape. For visuality only edges with weights in absolute value in the interval $[.3, \infty)$ are shown. Only weights above 0.5 in absolute value are distinguished in the represented edge's width.

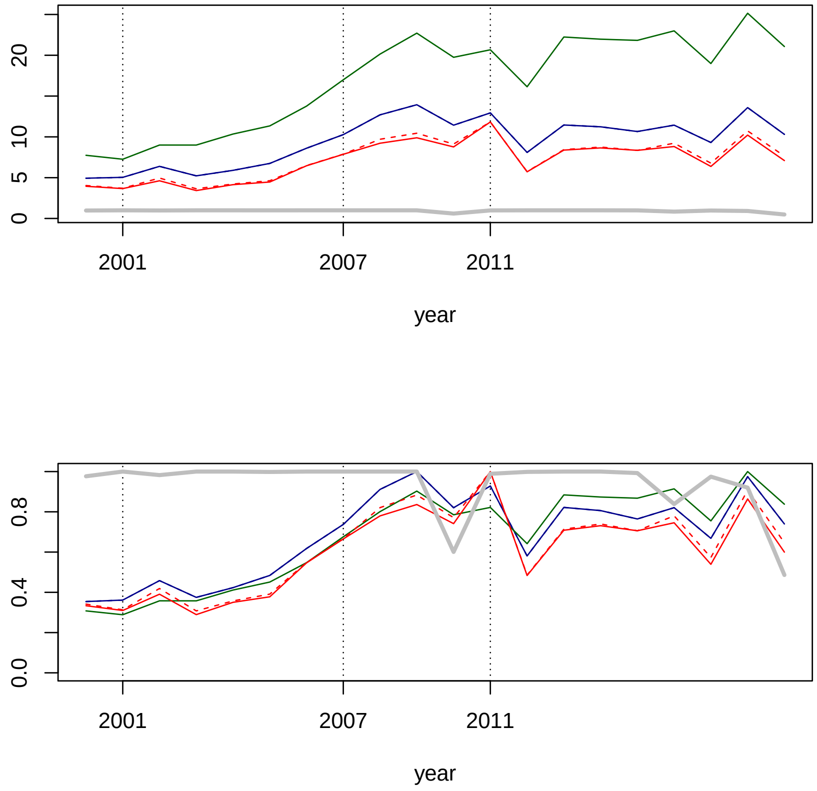


Figure 12: Network centralities from filtered Pearson networks based on the matrix $C^s$. The blue line is the largest eigenvalue. The green line is the maximum degree-centrality by year. The red line is an average of degree-centrality. The gray line represents the maximum betweenness-centrality. In the upper panel, time series are shown in their original scale, while in the lower panel time series have been rescaled by its maximum.

### 6.5 Modularity function $Q_3$

For the definition of the modularity function $Q_3$ the matrix $C^g$ is necessary and should not be a null matrix. For our data, this is the case for only a few years: 2000, 2010, 2016, 2018 and 2019. For them, a representation of communities can be seen from Figure 13. This is what we observe:

- First of all, in each year, there are only two communities as can be seen from the color of the vertexes, or equivalently from their shape. Interestingly, there is no clear larger community.

- Second, and also interesting, for our data, industrial sector is non determinant for the community assignment. More clearly, each industrial sector has vertexes in each community. This fact should be compared with the finding of Section 3.4 based on partial correlations where there also existed intersectorial links.

- This is our explanation of the years in which there existed a non trivial matrix $C^g$. First of all recall that this matrix represents structure just between the scales of individual stocks and the market as a whole, while in crisis periods this last structure is what prevails since stocks tend to be highly correlated at those times. In the year 2000 the peak of the dot.com bubble is located and for the years 2001 and 2002 bearish markets prevailed. What we see from Figure 11 for the network constructed from the matrices $C^s$ is an increase in interconnectedness while in Figure 13 we see that in the period 2000:2002 there existed a "mesoscopic" structure for the year 2000 in which there is a "local minimum" for graphs interconnectedness. Analogously for the year 2010 in Figure 13 which coincides with a local minimum in Figure 11 for the "extended" subprime crisis period 2007-2010.

- Now we compare the years 2016, 2018 and 2019 in Figures 11 and 13. Those are years in which global events occurred, to mention some of them: The Brexit (starting from its referendum in june 2016), US elections for the period 2017-2020, China-US trade conflict starting from july 2018. However, none of these seems to be comparable to the levels of the dot.com bubble and the subprime crisis. In particular for the Mexican stock market they didn't have such an impact as to hide the effects of a mesoscopic structure and inducing all stocks as moving according to a common factor.

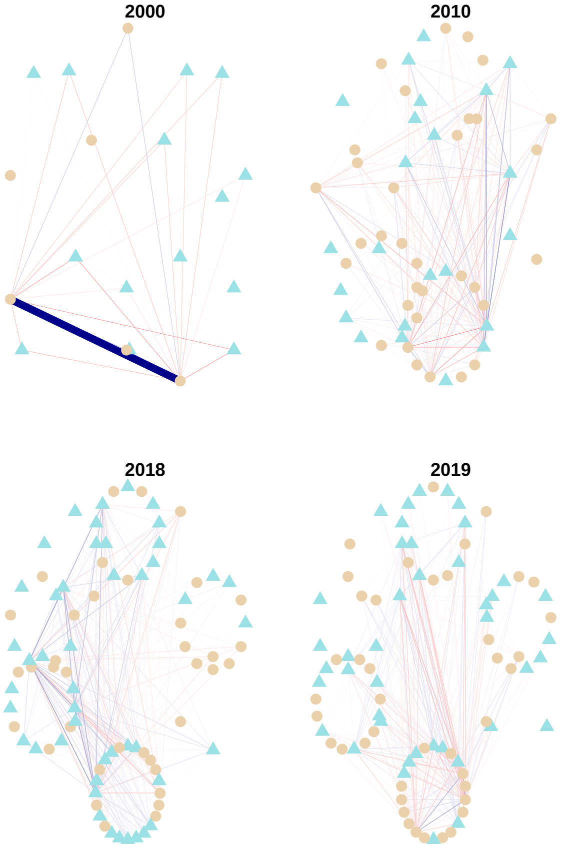


Figure 13: Communities from modularity function $Q_3$. The color of nodes identifies membership to the same community and equivalently for the vertex' shape. For visibility only edges with weights in absolute value in the intervals $[.05, \infty)$ are shown. Only weights above 0.5 in absolute value are distinguished in the represented edge's width.

## 6.6 Discussion

The methodology of [33] is what we adopt in this section. The authors of that paper obtain the result that for stocks in the index S&P500 the maximization of modularity without any sort of adjustment results in a unique community. This is uninteresting and also happens in our case with data from the Mexican stock exchange under the modularity function $Q_2$. It is based on the Pearson correlation matrix after noise has been filtered out according to Random Matrix Theory. This is the matrix $C^s$ which includes as we mentioned before the "market mode" from which the result of a unique community is unsurprising. In the paper [33] communities under $Q_3$ are also determined. They obtain five communities for stocks in the index S&P500. In our case there are

only two communities, but it is also true that year by year we are considering on average less than one-fifth on the number of stocks. In this sense, magnitude in the number of communities seems to be coherent. The configuration of community-structure on both cases shares two properties: (a) communities are multisectorial and (b) negative links joining nodes belonging to different communities are mostly negative. This is already interesting in the objective of understanding the interdependency structure in a single trustable snapshot. It also provides information for applications. For example, the fact that inter-communities links are negative is a useful taxonomy on the tasks of portfolio-allocation and hedging.

# 7 Conclusion

In global crisis periods, price levels of stocks in the Mexican stock exchange indeed present obvious changes which are visually evident and confirmed by econometric models as we have shown here and is also documented by other authors. However, the interdependency structure is a more complex phenomenon and much less studied. Our findings show that as long as partial-correlations are concerned, the interdependency structure is quite stable and centrality metrics from network theory have the sensibility to quantify small variations. Degree- and eigen-centralities indeed present variations, an upwards jump at the peak of the crisis and then a downwards jump when the shock of the crisis has been absorbed in the market. Another interesting finding of studying interdependency structure from partial-correlations is that only a small number of negative partial correlations which are also in magnitude small are present. We argue this is an indicator of a positive synergy of an integrated market. Reinforcing this claim, we find that industrial sectors are strongly interconnected even at the level of partial correlations, which is a less established property. In general and in particular for the Mexican case.
Estimation of networks based on different matrices indeed captures different aspects of interdependency. Tail-dependence networks and their centralities maxima gave the most precise timing for the crisis's heights (for the subprime and European debt crises). Interdependency from the point of view of ("absolute") correlations confirms findings from partial correlations. It also provides evidence of an integrated market for the Mexican case. Indeed, this is what we learned from the estimation of modularities which determined community structure without separating industrial sectors. From filtered matrices with noise filtered out (the matrices $C^s$) a single giant component emerged. Moreover, here the effect of global episodes for interdependency structure was quite clear even for visual appreciation. This is what we learned in Figure 11 and is perfect as evidence for the modeling strength. Indeed, correlations are more sensible to trading activity than partial-correlations and capture relationships among stocks due to such activity which is even more pronounced at crisis periods. We also studied community structure from the matrices $C^g$ which are the correlation matrices after noise and the global market mode have been filtered out. At this scale it happens that only a few observed years present a mesoscopic structure. For the years 2000 and 2010 in which mesoscopic structure is present, we observe a "local minimum" for interconnectedness in Figure 11. For the years 2016, 2018, 2019 we note also a turmoil of stress

periods (e.g., the Brexit, China-US trade conflict, etc.) which nevertheless are not to be compared in severity with the episodes of the dot.com bubble and the subprime crisis. Hence they are not able to blur the presence of structure at the mesoscopic level.

# A Partial correlations

In Table 4 we display partial correlations above the threshold 0.3 in absolute value.

Table 4: Links in the rank (0.3, 1] for the period 2000-2009.

| tick1 | tick2 | weight | year | tick1 | tick2 | weight | year |
|---|---|---|---|---|---|---|---|
| ELEKTRA | ICA | 0.98 | 2000 | ICHB | SIMECB | 0.44 | 2012 |
| FEMSAUBD | MXX | 0.4 | 2000 | AMXA | MXX | 0.83 | 2013 |
| AZTECACPO | SORIANAB | 0.31 | 2001 | FEMSAUBD | MXX | 0.42 | 2013 |
| AMXA | MXX | 0.37 | 2002 | GMEXICOB | MXX | 0.48 | 2013 |
| AZTECACPO | MXX | 0.38 | 2002 | ICHB | SIMECB | 0.34 | 2013 |
| CEMEXCPO | MXX | 0.38 | 2002 | MXX | WALMEX | 0.31 | 2013 |
| FEMSAUBD | MXX | 0.45 | 2002 | CEMEXCPO | MXX | 0.33 | 2014 |
| MXX | SORIANAB | 0.32 | 2002 | FEMSAUBD | MXX | 0.56 | 2014 |
| AMXA | MXX | 0.59 | 2003 | ASURB | GAPB | 0.36 | 2015 |
| AZTECACPO | MXX | 0.31 | 2003 | CEMEXCPO | MXX | 0.36 | 2015 |
| CEMEXCPO | MXX | 0.36 | 2003 | FEMSAUBD | MXX | 0.42 | 2015 |
| FEMSAUBD | MXX | 0.35 | 2003 | GFNORTEO | MXX | 0.37 | 2015 |
| MXX | WALMEX | 0.73 | 2003 | GMEXICOB | MXX | 0.33 | 2015 |
| AMXA | MXX | 0.72 | 2004 | ICHB | SIMECB | 0.36 | 2015 |
| CEMEXCPO | MXX | 0.49 | 2004 | CEMEXCPO | MXX | 0.45 | 2016 |
| MXX | WALMEX | 0.43 | 2004 | FEMSAUBD | MXX | 0.59 | 2016 |
| CEMEXCPO | MXX | 0.44 | 2005 | GFNORTEO | MXX | 0.34 | 2016 |
| MXX | WALMEX | 0.4 | 2005 | MXX | WALMEX | 0.35 | 2016 |
| CEMEXCPO | MXX | 0.49 | 2006 | ASURB | GAPB | 0.33 | 2017 |
| FEMSAUBD | MXX | 0.41 | 2006 | CEMEXCPO | MXX | 0.75 | 2017 |
| GMEXICOB | MXX | 0.34 | 2006 | FEMSAUBD | MXX | 0.42 | 2017 |
| MXX | WALMEX | 0.77 | 2006 | GEOB | HOMEX | 0.36 | 2017 |
| CEMEXCPO | MXX | 0.5 | 2007 | GFNORTEO | MXX | 0.47 | 2017 |
| GAPB | OMAB | 0.35 | 2007 | GMEXICOB | MXX | 0.3 | 2017 |
| GMEXICOB | MXX | 0.31 | 2007 | ICHB | SIMECB | 0.37 | 2017 |
| MXX | WALMEX | 0.56 | 2007 | ASURB | GAPB | 0.32 | 2018 |
| GAPB | OMAB | 0.31 | 2008 | CEMEXCPO | MXX | 0.74 | 2018 |
| ICHB | SIMECB | 0.41 | 2008 | FEMSAUBD | MXX | 0.87 | 2018 |
| MXX | WALMEX | 0.37 | 2008 | GFNORTEO | MXX | 0.65 | 2018 |
| CEMEXCPO | MXX | 0.37 | 2009 | GIGANTE | LIVEPOL1 | 0.32 | 2018 |
| GAPB | OMAB | 0.34 | 2009 | MXX | WALMEX | 0.41 | 2018 |
| ICHB | SIMECB | 0.34 | 2009 | ASURB | GAPB | 0.31 | 2019 |
| MXX | WALMEX | 0.35 | 2009 | CEMEXCPO | MXX | 0.39 | 2019 |
| CEMEXCPO | MXX | 0.35 | 2010 | FEMSAUBD | GFNORTEO | -0.36 | 2019 |
| ICHB | SIMECB | 0.34 | 2010 | FEMSAUBD | MXX | 0.89 | 2019 |
| MXX | WALMEX | 0.36 | 2010 | GAPB | OMAB | 0.33 | 2019 |
| FEMSAUBD | MXX | 0.35 | 2011 | GFNORTEO | MXX | 0.81 | 2019 |
| MXX | WALMEX | 0.33 | 2011 | GMEXICOB | MXX | 0.55 | 2019 |
| HOMEX | URBI | 0.32 | 2012 | MXX | WALMEX | 0.56 | 2019 |

Partial correlations in absolute value in the interval $(0.2, 0.3]$ are displayed in Table 5.

Table 5: Links in the rank $(0.2, 0.3]$ for the period 2000-2009.

| tick1 | tick2 | weight | year | tick1 | tick2 | weight | year |
|---|---|---|---|---|---|---|---|
| CEMEXCPO | MXX | 0.27 | 2000 | ASURB | GAPB | 0.24 | 2010 |
| GFINBURO | MXX | 0.21 | 2000 | AXTELCPO | GFAMSAA | 0.21 | 2010 |
| GFNORTEO | MXX | 0.23 | 2000 | GCARSOA1 | GFINBURO | 0.26 | 2010 |
| MXX | SORIANAB | 0.26 | 2000 | GFNORTEO | MXX | 0.21 | 2010 |
| ARA | GFNORTEO | 0.26 | 2001 | GMEXICOB | MXX | 0.29 | 2010 |
| AZTECACPO | ELEKTRA | 0.2 | 2001 | KUOA | LIVEPOL1 | 0.22 | 2010 |
| CEMEXCPO | FEMSAUBD | 0.3 | 2001 | ALFAA | MXX | 0.22 | 2011 |
| ALFAA | MXX | 0.2 | 2002 | CEMEXCPO | ICA | 0.28 | 2011 |
| AZTECACPO | ELEKTRA | 0.22 | 2002 | CEMEXCPO | MXX | 0.21 | 2011 |
| GFINBURO | MXX | 0.29 | 2002 | ELEKTRA | MXX | 0.22 | 2011 |
| GFNORTEO | MXX | 0.26 | 2002 | GFNORTEO | MXX | 0.3 | 2011 |
| ARA | MXX | 0.22 | 2003 | GMEXICOB | MXX | 0.29 | 2011 |
| GFINBURO | MXX | 0.21 | 2003 | HOMEX | URBI | 0.28 | 2011 |
| MXX | SORIANAB | 0.26 | 2003 | CEMEXCPO | MXX | 0.27 | 2012 |
| ALFAA | MXX | 0.29 | 2004 | FEMSAUBD | MXX | 0.28 | 2012 |
| AMXA | CEMEXCPO | -0.23 | 2004 | GMEXICOB | MXX | 0.26 | 2012 |
| AZTECACPO | MXX | 0.2 | 2004 | MXX | WALMEX | 0.22 | 2012 |
| BIMBOA | MXX | 0.2 | 2004 | ALFAA | MXX | 0.29 | 2013 |
| GFINBURO | MXX | 0.23 | 2004 | AMXA | FEMSAUBD | -0.22 | 2013 |
| GMEXICOB | MXX | 0.26 | 2004 | CEMEXCPO | MXX | 0.22 | 2013 |
| MXX | SORIANAB | 0.24 | 2004 | GFNORTEO | MXX | 0.26 | 2013 |
| ALFAA | MXX | 0.23 | 2005 | GMEXICOB | PE&OLES | 0.22 | 2013 |
| AMXA | MXX | 0.23 | 2005 | HOMEX | SAREB | 0.22 | 2013 |
| ARA | URBI | 0.21 | 2005 | ALFAA | MXX | 0.24 | 2014 |
| FEMSAUBD | MXX | 0.22 | 2005 | ALSEA | CULTIBAB | 0.24 | 2014 |
| GMEXICOB | MXX | 0.25 | 2005 | ASURB | GAPB | 0.2 | 2014 |
| KIMBERA | MXX | 0.21 | 2005 | GFNORTEO | MXX | 0.26 | 2014 |
| ALFAA | MXX | 0.22 | 2006 | GMEXICOB | MXX | 0.29 | 2014 |
| AMXA | MXX | 0.25 | 2006 | MFRISCOA-1 | PE&OLES | 0.27 | 2014 |
| GFINBURO | MXX | 0.28 | 2006 | ALFAA | MXX | 0.22 | 2015 |
| GFNORTEO | MXX | 0.2 | 2006 | GFINBURO | MXX | 0.22 | 2015 |
| MXX | PINFRA | 0.21 | 2006 | AC | BIMBOA | 0.2 | 2016 |
| BIMBOA | MXX | 0.23 | 2007 | ALFAA | AZTECACPO | 0.27 | 2016 |
| GFNORTEO | MXX | 0.23 | 2007 | ALFAA | GFINBURO | 0.22 | 2016 |
| HOMEX | MXX | 0.2 | 2007 | ASURB | GAPB | 0.21 | 2016 |
| ICA | MXX | 0.24 | 2007 | GENTERA | PINFRA | 0.23 | 2016 |
| MXX | URBI | 0.25 | 2007 | GMEXICOB | MXX | 0.21 | 2016 |
| ALFAA | ARA | 0.3 | 2008 | MFRISCOA-1 | PE&OLES | 0.27 | 2016 |
| ALFAA | AXTELCPO | 0.26 | 2008 | MXX | ORBIA | 0.2 | 2016 |
| AMXA | MXX | 0.23 | 2008 | ALFAA | ALPEKA | 0.24 | 2017 |
| CEMEXCPO | MXX | 0.26 | 2008 | CEMEXCPO | GFNORTEO | -0.24 | 2017 |
| FEMSAUBD | MXX | 0.26 | 2008 | GAPB | OMAB | 0.2 | 2017 |
| GCARSOA1 | MXX | 0.21 | 2008 | GFNORTEO | GMEXICOB | -0.22 | 2017 |
| GMEXICOB | MXX | 0.28 | 2008 | HOMEX | URBI | 0.28 | 2017 |
| HOMEX | MXX | 0.2 | 2008 | MXX | WALMEX | 0.23 | 2017 |
| MXX | PE&OLES | 0.25 | 2008 | CEMEXCPO | FEMSAUBD | -0.27 | 2018 |
| AMXA | MXX | 0.21 | 2009 | FEMSAUBD | GFNORTEO | -0.26 | 2018 |
| AMXA | RCENTROA | 0.21 | 2009 | GAPB | OMAB | 0.22 | 2018 |
| BIMBOA | MXX | 0.22 | 2009 | GMEXICOB | MXX | 0.26 | 2018 |
| FEMSAUBD | MXX | 0.21 | 2009 | ICHB | SIMECB | 0.22 | 2018 |
| GCARSOA1 | MXX | 0.22 | 2009 | GFNORTEO | GMEXICOB | -0.26 | 2019 |
| GMEXICOB | MXX | 0.26 | 2009 | HCITY | UNIFINA | 0.2 | 2019 |
| HOMEX | MXX | 0.25 | 2009 | | | | |

## B Persistent links from partial correlations

Table 6: Persistent links in the rank $(0.1, 1]$ for the period 2000-2009 (1/2).

| | 2000 | 2001 | 2002 | 2003 | 2004 | 2005 | 2006 | 2007 | 2008 | 2009 |
|---|---|---|---|---|---|---|---|---|---|---|
| ALFAA-MXX | 0.17 | | 0.20 | | 0.29 | 0.23 | 0.22 | | | |
| AMXA-MXX | 0.16 | | 0.37 | 0.59 | 0.72 | 0.23 | 0.25 | | 0.23 | 0.21 |
| CEMEXCPO-MXX | 0.27 | 0.14 | 0.38 | 0.36 | 0.49 | 0.44 | 0.49 | 0.50 | 0.26 | 0.37 |
| FEMSAUBD-MXX | 0.40 | 0.18 | 0.45 | 0.35 | 0.19 | 0.22 | 0.41 | 0.19 | 0.26 | 0.21 |
| GFINBURO-MXX | 0.21 | 0.16 | 0.29 | 0.21 | 0.23 | | 0.28 | | | 0.17 |
| GFNORTEO-MXX | 0.23 | | 0.26 | | | | 0.20 | 0.23 | | 0.20 |
| BIMBOA-MXX | | 0.11 | 0.17 | | 0.20 | 0.17 | | 0.23 | 0.14 | 0.22 |
| MXX-WALMEX | | | | 0.73 | 0.43 | 0.40 | 0.77 | 0.56 | 0.37 | 0.35 |
| GMEXICOB-MXX | | | | | 0.26 | 0.25 | 0.34 | 0.31 | 0.28 | 0.26 |
| ASURB-GAPB | | | | | | | | | 0.17 | 0.13 |
| ICHB-SIMECB | | | | | | | | | 0.41 | 0.34 |

Table 7: Persistent links in the rank $(0.1, 1]$ for the period 2010-2019 (2/2).

| | 2010 | 2011 | 2012 | 2013 | 2014 | 2015 | 2016 | 2017 | 2018 | 2019 |
|---|---|---|---|---|---|---|---|---|---|---|
| ALFAA-MXX | | 0.22 | 0.20 | 0.29 | 0.24 | 0.22 | | | | |
| AMXA-MXX | | | 0.12 | 0.83 | | | | | | |
| CEMEXCPO-MXX | 0.35 | 0.21 | 0.27 | 0.22 | 0.33 | 0.36 | 0.45 | 0.75 | 0.74 | 0.39 |
| FEMSAUBD-MXX | | 0.35 | 0.28 | 0.42 | 0.56 | 0.42 | 0.59 | 0.42 | 0.87 | 0.89 |
| GFINBURO-MXX | 0.14 | | | | | 0.22 | | | | 0.13 |
| GFNORTEO-MXX | 0.21 | 0.30 | 0.19 | 0.26 | 0.26 | 0.37 | 0.34 | 0.47 | 0.65 | 0.81 |
| BIMBOA-MXX | 0.18 | | | | 0.16 | 0.18 | 0.15 | 0.13 | 0.18 | |
| MXX-WALMEX | 0.36 | 0.33 | 0.22 | 0.31 | 0.20 | 0.19 | 0.35 | 0.23 | 0.41 | 0.56 |
| GMEXICOB-MXX | 0.29 | 0.29 | 0.26 | 0.48 | 0.29 | 0.33 | 0.21 | 0.30 | 0.26 | 0.55 |
| ASURB-GAPB | 0.24 | 0.17 | 0.16 | 0.16 | 0.20 | 0.36 | 0.21 | 0.33 | 0.32 | 0.31 |
| ICHB-SIMECB | 0.34 | | 0.44 | 0.34 | 0.11 | 0.36 | | 0.37 | 0.22 | |

# References


[1] Orazio Angelini Alexander Denev, Adrien Papaioannou, *A probabilistic graphical models approach to model interconnectedness*, International Journal of Risk Assessment and Management (2019).

[2] A. Almog, F. Besamusca, M. MacMahon, and D. Garlaschelli, *Mesoscopic community structure of financial markets revealed by price and sign fluctuations*, PloS One **10** (2015), no. 7.

[3] H.H. Andersen, M.H. Ojetre, D. Sorensen, and P.S. Eriksen, *Linear and graphical models for the multivariate complex normal distribution*, Lecture notes in statistics, Springer, 1995.

[4] Mikhail Anufriev and Valentyn Panchenko, *Connecting the dots: Econometric methods for uncovering networks with an application to the australian financial institutions*, Journal of Banking & Finance **61** (2015), S241 – S255.

[5] Frank Betz, Nikolaus Hautsch, Tuomas A. Peltonen, and Melanie Schienle, *Systemic risk spillovers in the european banking and sovereign network*, Journal of Financial Stability **25** (2016), 206–224.

[6] Vladimir Boginski, Sergiy Butenko, and Panos M. Pardalos, *Statistical analysis of financial networks*, Computational Statistics & Data Analysis **48** (2005), no. 2, 431 – 443.

[7] G. Bonanno, F. Lillo, and R.N. Mantegna, *High-frequency cross-correlation in a set of stocks*, Quantitative Finance **1** (2001), no. 1, 96–104.

[8] Ulrik Brandes, Daniel Delling, Marco Gaertler, Robert Görke, Martin Hoefer, Zoran Nikoloski, and D. Wagner, *Maximizing modularity is hard*, 2006.

[9] G.P. Clemente, R. Grassi, and A. Hitaj, *Asset allocation: new evidence through network approaches*, Annals of Operations Research (2019).

[10] A. P. Dempster, *Covariance selection*, Biometrics **28** (1972), no. 1, 157–175.

[11] Ginanjar Dewandaru, Syed Aun R. Rizvi, Rumi Masih, Mansur Masih, and Syed Othman Alhabshi, *Stock market co-movements: Islamic versus conventional equity indices with multi-timescales analysis*, Economic Systems **38** (2014), no. 4, 553 – 571.

[12] F. Emmert-Streib, A. Musa, K. Baltakys, J. Kanniainen, S. Tripathi, O. Yli-Harja, H. Jodlbauer, and M. Dehmer, *Computational analysis of structural properties of economic & financial networks*, Journal of Network Theory in Finance (2018), 1–32.

[13] R. Engle and K. Sheppard, *Theoretical and empirical properties of Dynamic Conditional Correlation Multivariate GARCH*, NBER Working Paper 8554 (2001).

[14] Robert Engle, *Dynamic conditional correlation*, Journal of Business & Economic Statistics **20** (2002), no. 3, 339–350.

[15] Santo Fortunato and Darko Hric, *Community detection in networks: A user guide*, Physics Reports **659** (2016), 1 – 44, Community detection in networks: A user guide.

[16] Jerome Friedman, Trevor Hastie, and Robert Tibshirani, *Sparse inverse covariance estimation with the graphical lasso*, Biostatistics **9** (2007), no. 3, 432–441.

[17] P. Giudici, P. Sarlin, and A. Spelta, *The interconnected nature of financial systems: Direct and common exposures*, Journal of Banking & Finance **112** (2020).

[18] P. Giudici and A. Spelta, *Graphical network models for international financial flows*, Journal of Business & Economic Statistics **34** (2016), no. 1, 128–138.

[19] Geoffrey Grimmett, *Probability on graphs: Random processes on graphs and lattices*, Institute of Mathematical Statistics Textbooks, Cambridge University Press, 2010.

[20] Xue Guo, Hu Zhang, and Tianhai Tian, *Development of stock correlation networks using mutual information and financial big data*, PLOS ONE **13** (2018), no. 4, 1–16.

[21] Wolfgang Karl Härdle, Weining Wang, and Lining Yu, *Tenet: Tail-event driven network risk*, Journal of Econometrics **192** (2016), no. 2, 499 – 513, Innovations in Multiple Time Series Analysis.

[22] Takashi Isogai, *Clustering of Japanese stock returns by recursive modularity optimization for efficient portfolio diversification**, Journal of Complex Networks **2** (2014), no. 4, 557–584.

[23] Aurora Jaramillo Olivares and Marcela Jaramillo Jaramillo, *Crisis financiera del 2008: efecto en las empresas listadas en la Bolsa Mexicana de Valores*, Revista mexicana de economía y finanzas **11** (2016), 161 – 177 (es).

[24] Zhi-Qiang Jiang and Wei-Xing Zhou, *Complex stock trading network among investors*, Physica A: Statistical Mechanics and its Applications **389** (2010), no. 21, 4929 – 4941.

[25] Dror Y. Kenett, Michele Tumminello, Asaf Madi, Gitit Gur-Gershgoren, Rosario N. Mantegna, and Eshel Ben-Jacob, *Dominating clasp of the financial sector revealed by partial correlation analysis of the stock market*, PLOS ONE **5** (2010), no. 12, 1–14.

[26] Bong-Han Kim, Hyeongwoo Kim, and Bong-Soo Lee, *Spillover effects of the u.s. financial crisis on financial markets in emerging asian countries*, International Review of Economics & Finance **39** (2015), 192 – 210.

[27] A. Lahrech and Kevin Sylwester, *U.S. and Latin American stock market linkages*, Journal of International Money and Finance **30** (2011), 1341–1357.

[28] Laurent Laloux, Pierre Cizeau, Jean-Philippe Bouchaud, and Marc Potters, *Noise dressing of financial correlation matrices*, Physical Review Letters **83** (1999), 1467–1470.

[29] S. L. Lauritzen, *Graphical models*, Oxford Science Publications, 1996.

[30] Bentian Li and Dechang Pi, *Analysis of global stock index data during crisis period via complex network approach*, PLOS ONE **13** (2018), no. 7, 1–16.

[31] Sibuya M., *Bivariate extreme statistics, i*, Annals of the Institute of Statistical Mathematics (1960), 195–210.

[32] M. (Ed.) Maathuis, M. (Ed.) Drton, S. (Ed.) Lauritzen, and M. (Ed.) Wainwright, *Handbook of graphical models*, Boca Raton: CRC Press, 2019.

[33] Mel MacMahon and Diego Garlaschelli, *Community detection for correlation matrices*, Phys. Rev. X **5** (2015).

[34] Rosario N. Mantegna, *Hierarchical structure in financial markets*, The European Physical Journal B - Condensed Matter and Complex Systems **11** (1999), 193–197.

[35] N. Meinshausen and P. Bühlmann, *High-dimensional graphs and variable selection with the lasso*, Ann. Statist. **34** (2006), no. 3, 1436–1462.

[36] T. Millington and M. Niranjan, *Partial correlation financial networks*, Appl Netw Sci **5** (2020), no. 11.

[37] Seema Narayan and Mobeen [Ur Rehman], *Diversification opportunities between emerging and frontier asian (efa) and developed stock markets*, Finance Research Letters **23** (2017), 223 – 232.

[38] M. E. J. Newman, *Modularity and community structure in networks*, Proceedings of the National Academy of Sciences **103** (2006), no. 23, 8577–8582.

[39] M. E. J. Newman and M. Girvan, *Finding and evaluating community structure in networks*, Phys. Rev. E **69** (2004), 026113.

[40] M.E.J. Newman, *Networks: An introduction*, Oxford University Press, 2010.

[41] J-P Onnela, Anirban Chakraborti, Kimmo Kaski, János Kertész, and Antti J. Kanto, *Dynamics of market correlations: taxonomy and portfolio analysis.*, Physical review. E, Statistical, nonlinear, and soft matter physics **68 5 Pt 2** (2003), 056110.

[42] Guillermo Benavides Perales, *Central Bank Exchange Rate Interventions and Market Expectations: The Case of México During the Financial Crisis 2008-2009*, Revista Mexicana de Economía y Finanzas Nueva Época REMEF **6** (2017), no. 1.

[43] Gustavo Peralta and Abalfazl Zareei, *A network approach to portfolio selection*, Journal of Empirical Finance **38** (2016), 157 – 180.

[44] C. Piccardi, L. Calatroni, and F. Bertoni, *Clustering financial time series by network community analysis*, International Journal of Modern Physics C **22** (2011), no. 01, 35–50.

[45] Vasiliki Plerou, P. Gopikrishnan, Bernd Rosenow, Luis A. Nunes Amaral, Thomas Guhr, and Harry Eugene Stanley, *Random matrix approach to cross correlations in financial data.*, Physical review. E, Statistical, nonlinear, and soft matter physics **65 6 Pt 2** (2002), 066126.

[46] F. Pozzi, T. Di Matteo, and T. Aste, *Spread of risk across financial markets: better to invest in the peripheries*, Scientific Reports (2013).

[47] Rafael Schmidt and Ulrich Stadtmüller, *Non-parametric estimation of tail dependence*, Scandinavian Journal of Statistics **33** (2006), no. 2, 307–335.

[48] Luis Ignacio Roman de la Sancha, Federico Hernandez Alvarez, and Gabriel Rodriguez Garcia, *Co-movimientos entre los Índices Accionarios y los Ciclos Económicos de Estados Unidos y México*, Revista mexicana de economía y finanzas **14** (2019), 693 – 714.

[49] Roberto J. Santillán Salgado, *Is the Mexican Stock Market Becoming More Efficient?*, Revista Mexicana de Economía y Finanzas Nueva Época REMEF **6** (2017), no. 1.

[50] Leonidas Sandoval and Italo De Paula Franca, *Correlation of financial markets in times of crisis*, Physica A: Statistical Mechanics and its Applications **391** (2012), no. 1, 187 – 208.

[51] L. Sandoval Junior, *Networks of log returns and volatilities of international stock market indexes*, Journal of Network Theory in Finance (2017), 41–82.

[52] Frank Schweitzer, Giorgio Fagiolo, Didier Sornette, Fernando Vega-Redondo, Alessandro Vespignani, and Douglas R. White, *Economic networks: The new challenges*, Science **325** (2009), no. 5939, 422–425.

[53] Syed Jawad Hussain Shahzad, Jose Areola Hernandez, Mobeen Ur Rehman, Khamis Hamed Al-Yahyaee, and Muhammad Zakaria, *A global network topology of stock markets: Transmitters and receivers of spillover effects*, Physica A: Statistical Mechanics and its Applications **492** (2018), 2136 – 2153.

[54] Helene Shapiro, *Linear algebra and matrices*, AMS, 2015.

[55] Fa-Bin Shi, Xiao-Qian Sun, Hua-Wei Shen, and Xue-Qi Cheng, *Detect colluded stock manipulation via clique in trading network*, Physica A: Statistical Mechanics and its Applications **513** (2019), 565 – 571.

[56] J. Sidaoui, M. Ramos-Francia, and G. Cuadra, *The Global Financial Crisis and Policy Response in Mexico, Mexico City: Bank of Mexico. Basilea, Bank for International Settlement*, BIS papers no. 54 (2010).

[57] Robert Tibshirani, *Regression shrinkage and selection via the lasso*, Journal of the Royal Statistical Society. Series B (Methodological) **58** (1996), no. 1, 267–288.

[58] Chi K. Tse, Jing Liu, and Francis C.M. Lau, *A network perspective of the stock market*, Journal of Empirical Finance **17** (2010), no. 4, 659 – 667.

[59] Y. K Tse and Albert K. C Tsui, *A multivariate generalized autoregressive conditional heteroscedasticity model with time-varying correlations*, Journal of Business & Economic Statistics **20** (2002), no. 3, 351–362.

[60] Irena Vodenska, Hideaki Aoyama, Yoshi Fujiwara, Hiroshi Iyetomi, and Yuta Arai, *Interdependencies and causalities in coupled financial networks*, PLOS ONE **11** (2016), no. 3, 1–32.

[61] M.J. Wainwright and M.I. Jordan, *Graphical models, exponential families, and variational inference*, Found. Trends Mach. Learn. **1** (2008), no. 1-2, 1–305.

[62] G. Wang, C. Xie, and H.E. Stanley, *Correlation structure and evolution of world stock markets: Evidence from pearson and partial correlation-based networks*, Computational Economics **51** (2018), no. 3, 607–635.

[63] Gang-Jin Wang and Chi Xie, *Tail dependence structure of the foreign exchange market: A network view*, Expert Systems with Applications **46** (2016), 164 – 179.

[64] Fenghua Wen, Xin Yang, and Wei-Xing Zhou, *Tail dependence networks of global stock markets*, International Journal of Finance & Economics **24** (2019), no. 1, 558–567.

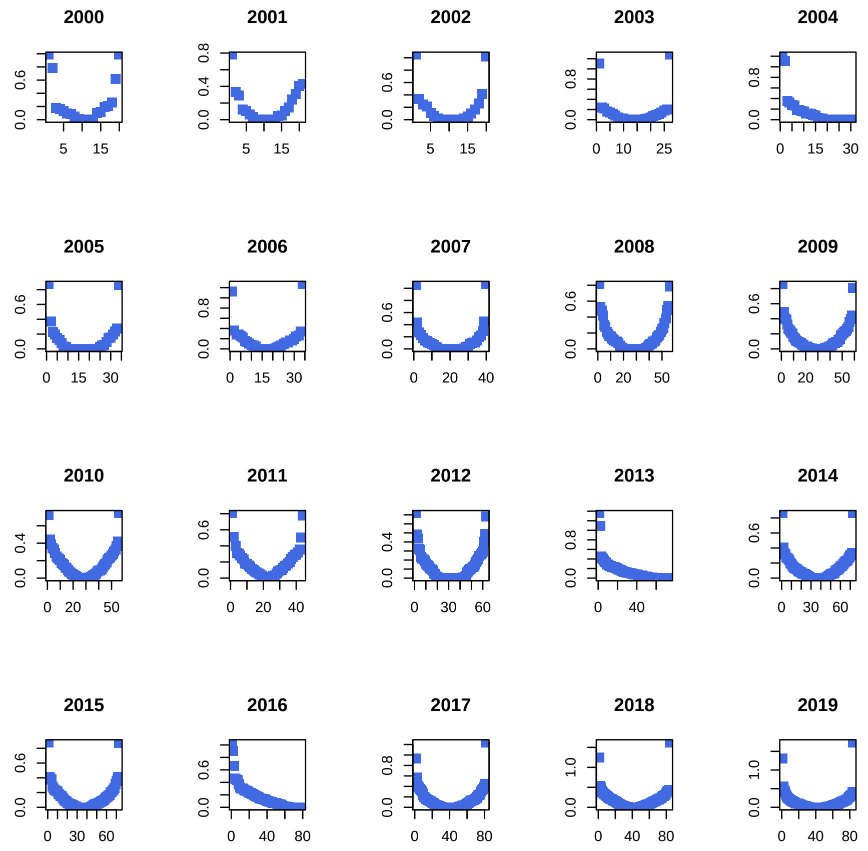


Figure 7: Eigenvalue’s modulus per year.
